# Nonsteroidal anti-inflammatory drug choice and adverse outcomes in clopidogrel users: A retrospective cohort study

**DOI:** 10.1371/journal.pone.0193800

**Published:** 2018-03-14

**Authors:** Young Hee Nam, Colleen M. Brensinger, Warren B. Bilker, Charles E. Leonard, Scott E. Kasner, Tilo Grosser, Xuanwen Li, Sean Hennessy

**Affiliations:** 1 Center for Pharmacoepidemiology Research and Training, Department of Biostatistics, Epidemiology and Informatics, Perelman School of Medicine, University of Pennsylvania, Philadelphia, PA, United States of America; 2 Center for Clinical Epidemiology and Biostatistics, Department of Biostatistics, Epidemiology and Informatics, Perelman School of Medicine, University of Pennsylvania, Philadelphia, PA, United States of America; 3 Department of Neurology, Perelman School of Medicine, University of Pennsylvania, Philadelphia, PA, United States of America; 4 Department of Systems Pharmacology and Translational Therapeutics, Perelman School of Medicine, University of Pennsylvania, Philadelphia, PA, United States of America; University of Manitoba, CANADA

## Abstract

**Objective:**

To examine the comparative safety of individual NSAIDs when given concomitantly with clopidogrel.

**Methods:**

We conducted a retrospective cohort study using Medicaid claims from five US states during 1999–2010, supplemented with Medicare claims for dual-enrollees. The exposure of interest was the first concomitant use of clopidogrel and one of the 10 selected NSAIDs after a 1-year baseline period. The outcomes were: all-cause mortality; acute myocardial infarction (AMI)/ischemic stroke; and gastrointestinal bleeding (GIB)/intracranial hemorrhage (ICH). We calculated the hazard ratio of each NSAID for each outcome, with ibuprofen as the reference drug, using high-dimensional propensity score-adjusted proportional-hazards regression models.

**Results:**

Of 1,060,412 clopidogrel users, 268,114 concomitant NSAID users met inclusion/exclusion criteria, contributing 48,483 person-years. We observed 2,463 deaths, 2,822 AMI/ischemic stroke outcomes, and 2,620 GIB/ICH outcomes, for unadjusted incidence rates of 50.8, 58.6, and 54.3 per 1,000 person-years, respectively. Compared with ibuprofen and controlling for potential confounders, rofecoxib (hazard ratio [HR] = 1.22; 95% confidence interval [CI]: 1.04, 1.43) and valdecoxib (HR = 0.66; 95% CI: 0.48, 0.92) showed higher and lower hazards of mortality, respectively. Indomethacin showed an increased AMI/ischemic stroke hazard (HR = 1.38; 95% CI: 1.09, 1.74). For GIB/ICH, indomethacin (HR = 2.18; 95% CI: 1.74, 2.73), diclofenac (HR = 1.65; 95% CI: 1.39, 1.97), naproxen (HR = 1.47; 95% CI: 1.28, 1.70), and rofecoxib (HR = 1.26; 95% CI: 1.08, 1.48) showed higher hazards, and valdecoxib (HR = 0.73; 95% CI: 0.55, 0.98) showed a lower hazard.

**Conclusion:**

The bleeding risks of individual NSAIDs varied more markedly than thrombotic risks when used concomitantly with clopidogrel. Moreover, bleeding risk and thrombotic risk among individual NSAIDs did not appear to be inversely related to each other in the presence of clopidogrel. Further studies are needed to elucidate underlying biological mechanisms and help clinical decision-making for a better NSAID choice in clopidogrel users.

## Introduction

Nonsteroidal anti-inflammatory drugs (NSAIDs) are commonly used to treat pain and inflammation. In 2010, more than 29 million adults in the United States used an NSAID at least three times a week for at least three months [1]. Recent studies have suggested that individual NSAIDs may differ substantially with regard to the risk of acute myocardial infarction (AMI) [2–12], stroke [2–3,7–9,13–14], bleeding [8–9,15], and cardiovascular and all-cause death [2–3,6–9]. Since some NSAIDs may reduce the antiplatelet benefits of aspirin by competing directly for the same binding site on platelet cyclooxygenase (COX)-1 [16], other antiplatelet agents such as clopidogrel have been proposed for patients requiring both platelet inhibition and an NSAID [16–17]. Clopidogrel is widely used to reduce the risk of atherosclerotic events in patients who have had a recent AMI or stroke, and those with peripheral artery disease or acute coronary syndrome [18–19]. Clopidogrel needs to be converted to an active metabolite through a multistep process mediated by multiple cytochrome P450 (CYP) isozymes [20]. Although this need for activation has been hypothesized to result in drug-drug interactions with other CYP enzyme-metabolized drugs (including some NSAIDs), the clinical impact of such interactions is uncertain. In addition to potential CYP-based mechanisms, clopidogrel and NSAIDs might also interact through effects on the function of platelets [21], which is important to both atherothrombosis and hemostasis [22]. In particular, NSAIDs can either promote or suppress platelet aggregation through various mechanisms, and therefore might either increase or reduce the risk of thrombotic and/or hemorrhagic events. For example, NSAIDs can reduce platelet aggregation by inhibiting thromboxane [21] or reducing inflammation [23]. On the other hand, NSAIDs can promote platelet aggregation by suppressing the synthesis of prostacyclin, a potent endogenous platelet inhibitor [24]. Clopidogrel’s antiplatelet activity may also increase the risk of bleeding complications [25–26]. How these multiple mechanisms interact with each other is unknown, and the current lack of knowledge about the comparative safety of NSAIDs in those who take clopidogrel can complicate treatment decisions for many patients with cardiovascular conditions who need to manage pain and/or inflammation. Our study therefore aimed to examine the comparative safety of NSAIDs with regard to all-cause mortality, AMI/ischemic stroke (as a composite endpoint), and gastrointestinal bleeding (GIB)/intracranial hemorrhage (ICH, which includes hemorrhagic stroke) (as a composite endpoint) among users of clopidogrel, using large-scale real-world data.

## Methods

### Study design, population, and data

We conducted a retrospective cohort study using Medicaid claims data from five US states (California, Florida, New York, Ohio, and Pennsylvania) from 1999–2010 [27]. Medicaid is a health insurance program funded by the federal and state governments that provides health coverage to nearly 70 million Americans, including eligible people with low-income or disabilities [28]. Nationwide, approximately 43%, 34%, 14%, and 9% of the Medicaid enrollees account for children, adults (under age 65 years), people with disability (under age 65 years), and older people (age 65 years and older), respectively [29]. About 15% of the Medicaid enrollees are dually enrolled in Medicare [30], and among Medicaid-Medicare dual-enrollees, about 2.4% has end-stage renal disease [31]. Our five states comprise about 40% of the national Medicaid population [32]. The 12-year Medicaid data from these five states include about 65 million cumulative enrollees and 200 million person-years of records. For those also enrolled in Medicare, we also used Medicare claims data from 1999–2010, including Part D data from 2006–2010. Medicare is the federal program providing health insurance for about 58 million beneficiaries, including people who are age 65 years and older, certain younger people with disabilities, and people with end-stage renal disease [33–34]. Part D is an outpatient prescription drug coverage program of Medicare [35]. Our study population was defined as adults (18 ≤ age ≤ 100 years) with continuous enrollment in Medicaid during a one-year baseline period before the cohort entry date (explained below).

### Study cohort

The study cohort consisted of apparently new concomitant users of clopidogrel and one of the following NSAIDs, which were the ten most frequently prescribed NSAIDs in the study population: ibuprofen, celecoxib, naproxen, rofecoxib, meloxicam, diclofenac, indomethacin, valdecoxib, nabumetone, and etodolac (in descending order by the number of users); these ten NSAIDs accounted for about 98% of all NSAID users in our data. Although rofecoxib and valdecoxib were withdrawn from the US market during our study period (in 2004 and 2005, respectively), we included these drugs since their inclusion might contribute to mechanistic insights. The cohort entry date was defined as the first date of concomitant use of clopidogrel plus an NSAID, irrespective of the initiation order of clopidogrel and NSAID [36]. Dates of drug use were estimated by the dispensing date and the days’ supply field. The one year prior to the cohort entry date defined the baseline period. Therefore, the earliest possible cohort entry in our data was in 2000. Since we did not intend to study only incident events, we did not exclude persons with a prior AMI, ischemic stroke, or GIB/ICH. Procedures for the identification of study cohorts and the application of inclusion and exclusion criteria are presented in **S1 Fig.**

### Exposure and follow-up time

The exposure of interest was defined as the first concomitant use of clopidogrel plus one of the ten study NSAIDs dispensed as orally-administered, solid dosage forms, identified by the outpatient prescription drug claims. Prescriptions drug use was identified by using National drug Codes and days’ supply on prescription claims. We allowed a 15-day gap between contiguous prescriptions and at the end of the last prescription, to allow for potential incomplete adherence. Each NSAID defined a different exposure group. Ibuprofen was selected as the reference exposure because it was the most commonly used NSAID in the cohort. We excluded person-time during which more than one NSAID were used. Among the ten NSAIDs, over-the-counter formulations were available for lower strengths of ibuprofen and naproxen. Thus, it is possible that some exposures to these drugs were not captured. However, it seems that it would be uncommon for a clopidogrel user to take both a prescription and a nonprescription NSAID, particularly since prescription NSAIDs are paid for by Medicaid.

Follow-up time began on the cohort entry date and ended on the date of the first-occurring of the following events: 1) outcome of interest; 2) end of the days’ supply of clopidogrel or the exposure-defining NSAID (allowing for a 15-day grace period); 3) dispensing of an NSAID other than the exposure-defining NSAID (suggestive of switching to a different drug), for which the cohort end date was set to be the day before the non-exposure-defining NSAID prescription was dispensed; 4) disenrollment from Medicaid; and 5) end of the dataset, which was December 31, 2010. The follow-up time was independently determined for each of our three outcomes (i.e., all-cause mortality, AMI/ischemic stroke, or GIB/ICH), and each patient contributed person-time to only one NSAID. In a sensitivity analysis that restricted the follow-up period to the first 180 days after cohort entry, the 180th day from the cohort entry also served to censor follow-up time.

### Ascertainment of outcomes

The outcomes of interest were all-cause mortality and two composite outcomes: 1) AMI/ischemic stroke and 2) GIB/ICH. All-cause mortality was ascertained by linkage to the Social Security Administration Death Master File. The definition of the components of our composite outcomes and the performance measures of the ascertainment algorithms are presented in **Table 1**. Based on prior studies, the positive predictive values of the outcome-specific algorithms range from 81% to 98% [37–40].

**Table 1:** Operational definition of the components of composite outcomes.

Outcome	Discharge diagnosis position and claim type	ICD-9-CM^†^ discharge diagnosis code(s)	Performance metrics / validity measures of the algorithm
Acute myocardial infarction	Principal or non-principal diagnosis, inpatient claims	410.*1	PPV^‡^ ≈ 94% [37]
Ischemic stroke	Principal diagnosis, inpatient claims; excluding patients with intracranial injury diagnosis (ICD-9-CM codes, 800*-804*, 850*-854*) as secondary diagnosis on the same admission	433.*1, 434* (excluding 434.*0), or 436*	PPV ≈ 88% [38] Specificity ≈ 95% Sensitivity ≈ 74%
Gastrointestinal bleeding	Principal or non-principal diagnosis, inpatient claims	530.21, 531.0*, 531.2*, 531.4*, 531.6*, 532.0*, 532.2*, 532.4*, 532.6*, 533.0*, 533.2*, 533.4*, 533.6*, 534.0*, 534.2*, 534.4*, 534.6*, 535.01, 535.11, 535.21, 535.31, 535.41, 535.51, 535.61, 535.71, 537.83, 537.84, 562.02, 562.03, 562.12, 562.13, 569.85, 569.86, or 578.*	PPV ≈ 81% [39]
Intracranial hemorrhage (including hemorrhagic stroke)	Principal or non-principal diagnosis, inpatient or ED^§^ claims; excluding patients with intracranial injury diagnosis (ICD-9-CM codes, 800*-804*, 850*-854*) on the same admission	430 or 431	PPV ≈ 97% (ICH^‖^), 98% (SAH^#^) [40]

^†^ICD-9-CM: International Classification of Diseases 9th Revision Clinical Modification.

^‡^PPV: positive predictive value.

^§^ED: emergency department.

^‖^ICH: intracerebral hemorrhage.

^#^SAH: subarachnoid hemorrhage.

### Statistical analysis

We first calculated descriptive statistics, including the baseline characteristics of the study cohort and unadjusted incidence rates and hazard ratios of the outcomes by NSAID exposure group. To assess balance in measured baseline covariates between the different NSAID-exposure groups, we calculated the standardized difference and the weighted conditional standardized difference (WCSD), before and after calculating propensity scores, respectively. The standardized difference represents the mean difference of a variable between the two groups in units of the estimated common standard deviation of that variable in the two groups [41]. After calculating propensity scores, we used the WCSD to assess whether two comparison groups had similar distributions of measured baseline covariates conditional on the propensity score [41]. Literature suggests that standardized difference and WCSD values exceeding 0.1 may indicate potentially meaningful imbalance between groups [41].

Next, we used a high-dimensional propensity score (hdPS)-adjusted [42–46] proportional-hazards models to calculate the hazard ratio of each NSAID for each outcome, with ibuprofen as the reference drug. For the propensity score calculation, we used a multinomial logistic regression model that included pre-specified covariates (N = 135), as well as covariates identified empirically by the hdPS method (N = 596). The pre-specified covariates were chosen based on potential association with both exposure and outcomes of interest, including: a) demographic factors (e.g., age, sex, race/ethnicity, state of residence, etc.); b) healthcare services utilization intensity (e.g., number of circulatory system hospitalizations, number of circulatory system emergency department visits, number of unique outpatient diagnosis codes, number of outpatient ICD-9 procedure codes, number of prescription dispensing, etc.); c) diseases (e.g., hypertension, diabetes mellitus, cancer, conduction disorders, lipid metabolism disorder, osteoarthritis, rheumatoid arthritis, etc.); and d) prescription drugs (e.g., anticoagulants, aspirin, statins, fibrates, etc.) (**S1 Table**). These covariates were measured as binary variables during the baseline period, except for age at cohort entry, a continuous variable. The specifications used in the hdPS method are presented in **S2 Table**, and the hdPS-identified covariates are presented in **S3 Table**. As shown in **S2 Table**, we specified 9 dimensions of data (inpatient ICD-9 diagnoses; inpatient ICD-9 procedures; inpatient CPT/HCPCS procedures; outpatient ICD-9 diagnoses; outpatient ICD-9 procedures; outpatient CPT/HCPCS procedures; other setting ICD-9 diagnoses; other setting ICD-9 procedures; and outpatient medication active ingredients), and identified 200 covariates with the highest frequencies in the claims data for each dimension (N = 1,800), and selected top 500 covariates with the largest likelihood of confounding (N = 500). We performed this procedure for each of the 9 pairs of NSAID of interest vs. ibuprofen, separately (N = 4,500). Of these, we excluded overlapping (identical) covariates (N = 3,856), covariates with the number of persons exposed less than 10 (N = 42), and covariates overlapping with pre-specified covariates (N = 6). Thus, 596 empirically identified covariates, along with 135 pre-specified covariates, were used in the multinomial logistic regression model to calculate propensity scores. In the proportional hazards models, we included the propensity scores as continuous variables, as well as covariates with the WCSD greater than 0.1 (presented in **S1** and **S4 Tables**). We tested the proportional-hazard assumption of the each model, and based on the results, we included a time-by-NSAID interaction term. We also performed subgroup analysis as secondary analysis, stratified by: a) age (18 ≤ age < 65 years; 65 ≤ age ≤ 100 years); b) sex (male; female); and c) concomitancy-triggering drug (NSAID-triggered group; clopidogrel-triggered group and combination-triggered group), separately [36]. When an NSAID was added to the clopidogrel therapy, it is called NSAID-triggered concomitancy; when clopidogrel was added to an NSAID, it is called clopidogrel-triggered concomitancy; and when clopidogrel and an NSAID started on the same date, it is called combination-triggered concomitancy [36].

### Sensitivity analysis

In addition, we conducted two sensitivity analyses. In the first sensitivity analysis, we censored the follow-up time at 180 days after the cohort entry date. In the second sensitivity analysis, we excluded patients who had potentially incomplete data, such as those in managed care plans.

Data were analyzed using SAS version 9.4 (SAS Institute Inc., Cary, North Carolina). This study was approved by the institutional review board of the University of Pennsylvania, which waived the requirement for obtaining informed consent.

## Results

### Cohort characteristics and unadjusted incidence rates and hazard ratios of outcomes

The numbers of unique users of clopidogrel and any NSAIDs were 1,060,412 and 11,825,916, respectively (**S1 Fig**). Of these, 403,833 patients had overlapping prescriptions of clopidogrel and any NSAID. After applying inclusion and exclusion criteria, the study cohort consisted of 268,114 patients who contributed 48,483 person-years of concomitant exposure to clopidogrel plus one of the ten study NSAIDs. Selected baseline characteristics of the study cohort are presented in **Table 2**. **S4 Table** shows a complete table of the pre-specified baseline characteristics, including demographic characteristics, healthcare utilization factors, diseases, and prescription drugs, in addition to the outcomes of interest during the baseline period. The median age at cohort entry was about 66.2–73.4 years, depending on the NSAID exposure group, with 53.7–76.9% being 65 years of age or older.

**Table 2:** Baseline characteristics of clopidogrel users by NSAID exposure group.

		**ibuprofen**	**celecoxib**			**diclofenac**			**etodolac**			**indomethacin**			**meloxicam**		
		**(N = 69,779)**	**(N = 66,317)**			**(N = 18,593)**			**(N = 2,807)**			**(N = 7,651)**			**(N = 25,459)**		
**Characteristic**	**Group**	**%**	**%**	**S.Diff**^**†**^	**WCSD**^**‡**^	**%**	**S.Diff**	**WCSD**	**%**	**S.Diff**	**WCSD**	**%**	**S.Diff**	**WCSD**	**%**	**S.Diff**	**WCSD**
Age at cohort entry (continuous; years)	Median (Q3-Q1)	Age = 66.7 (56.1–75.4)	73.4 (65.9–80.3)	0.54	0.25	71.0 (62.2–78.2)	0.31	0.17	66.2 (55.6–74.7)	0.03	0.10	70.1 (60.1–77.9)	0.23	0.19	72.3 (64.2–79.4)	0.43	0.25
Age group at cohort entry	18 to <35	1.1	0.2	0.11	0.03	0.4	0.09	0.02	0.9	0.02	0.03	0.6	0.06	0.02	0.2	0.11	0.02
	35 to <50	12.3	4.0	0.31	0.07	6.6	0.19	0.05	12.8	0.02	0.05	8.1	0.14	0.07	4.7	0.27	0.04
	50 to <65	32.0	18.8	0.31	0.17	23.5	0.19	0.12	32.6	0.01	0.08	26.7	0.12	0.13	21.5	0.24	0.09
	65 to <80	40.2	50.9	0.22	0.14	49.6	0.19	0.14	39.5	0.01	0.07	45.4	0.11	0.09	50.2	0.20	0.13
	80 to ≤100	14.4	26.0	0.29	0.13	19.9	0.15	0.06	14.2	0.01	0.11	19.3	0.13	0.11	23.4	0.23	0.13
Sex	Female	56.3	67.2	0.23	0.08	64.3	0.16	0.06	63.8	0.15	0.11	45.5	0.22	0.24	65.9	0.20	0.07
Race/ethnicity	White	40.4	42.6	0.05	0.10	42.2	0.04	0.09	57.5	0.35	0.10	46.4	0.12	0.14	41.7	0.03	0.12
	Black	16.6	10.9	0.17	0.09	10.5	0.18	0.12	14.5	0.06	0.08	17.9	0.03	0.07	10.0	0.20	0.08
	Hispanic/Latino	21.2	14.6	0.17	0.15	25.8	0.11	0.29	11.6	0.26	0.14	10.1	0.31	0.25	15.0	0.16	0.14
	Other/Unknown	21.7	31.8	0.23	0.13	21.6	0.00	0.17	16.3	0.14	0.12	25.6	0.09	0.14	33.3	0.26	0.11
		**ibuprofen**	**nabumetone**			**naproxen**			**rofecoxib**			**valdecoxib**					
		**(N = 69,779)**	**(N = 7,060)**			**(N = 36,577)**			**(N = 26,247)**			**(N = 7,624)**					
**Characteristic**	**Group**	**%**	**%**	**S.Diff**	**WCSD**	**%**	**S.Diff**	**WCSD**	**%**	**S.Diff**	**WCSD**	**%**	**S.Diff**	**WCSD**			
Age at cohort entry (continuous; years)	Median (Q3-Q1)	Age = 66.7 (56.1–75.4)	69.3 (59.3–77.4)	0.19	0.13	67.2 (56.9–75.9)	0.05	0.03	72.7 (64.1–79.8)	0.44	0.46	72.1 (64.1–79.2)	0.41	0.52			
Age group at cohort entry	18 to <35	1.1	0.5	0.07	0.03	0.9	0.02	0.01	0.4	0.09	0.07	0.3	0.10	0.08			
	35 to <50	12.3	8.9	0.11	0.05	10.9	0.04	0.01	5.3	0.25	0.22	5.0	0.26	0.22			
	50 to <65	32.0	27.5	0.10	0.07	31.8	0.01	0.02	21.2	0.25	0.44	21.9	0.23	0.46			
	65 to <80	40.2	44.9	0.10	0.09	41.2	0.02	0.03	48.6	0.17	0.20	50.4	0.21	0.22			
	80 to ≤100	14.4	18.2	0.10	0.06	15.2	0.02	0.02	24.5	0.26	0.20	22.4	0.21	0.25			
Sex	Female	56.3	67.5	0.23	0.08	60.6	0.09	0.05	69.0	0.26	0.12	70.8	0.31	0.13			
Race/ethnicity	White	40.4	48.2	0.16	0.08	42.2	0.04	0.06	48.7	0.17	0.11	50.4	0.20	0.07			
	Black	16.6	12.6	0.11	0.07	16.3	0.01	0.04	11.8	0.14	0.13	10.8	0.17	0.12			
	Hispanic/Latino	21.2	17.9	0.08	0.12	18.7	0.06	0.06	12.5	0.24	0.21	12.9	0.22	0.20			
	Other/Unknown	21.7	21.2	0.01	0.10	22.8	0.02	0.05	27.0	0.12	0.25	25.9	0.10	0.13			

The full table of the characteristics of clopidogrel users by NSAID exposure group is presented in **S4 Table**.

^†^S.Diff: standardized difference vs. ibuprofen.

^‡^WCSD: weighted conditional standardized difference vs. ibuprofen.

In the primary analysis (**Table 3**), we identified 2,463 deaths, 2,822 AMI/ischemic stroke events (AMI: 1,812; ischemic stroke: 1,030; both: 19), and 2,620 GIB/ICH events (GIB: 2,441; ICH: 182; both: 3); the proportion of the number of ICH events was relatively small (about 7% of the composite outcome). The median follow-up time was 46 days for each of the three outcomes. **Table 3** shows the unadjusted incidence rates and hazard ratios of each outcome by NSAID exposure group. In the primary analysis, the overall incidence rates (in events per 1,000 person-years) for all-cause mortality, AMI/ischemic stroke, and GIB/ICH were about 50.8 (95% confidence interval [CI]: 48.8, 52.9), 58.6 (95% CI: 56.4, 60.8), and 54.3 (95% CI: 52.3, 56.5), respectively. The unadjusted incidence rates in the sensitivity analysis are presented in **S5 Table**.

**Table 3:** Primary analysis: Unadjusted incidence rates and hazard ratios of outcomes by NSAID exposure group.

Outcome	NSAID	Number of users	Number of events	Person-years	Incidence rate (per 1,000 p-ys*)	95% CI^†^ of incidence rate	Hazard ratio	95% CI of hazard ratio
**All-cause mortality**	Overall	268,114	2,463	48,483	50.8	48.8–52.9		
	celecoxib	66,317	901	15,930	56.6	52.9–60.4	1.28	1.14–1.44
	diclofenac	18,593	138	3,379	40.8	34.3–48.3	0.90	0.74–1.09
	etodolac	2,807	20	536	37.3	22.8–57.7	0.83	0.53–1.30
	ibuprofen	69,779	414	8,667	47.8	43.3–52.6	*Reference drug*	
	indomethacin	7,651	57	834	68.3	51.8–88.5	1.40	1.06–1.85
	meloxicam	25,459	191	5,277	36.2	31.2–41.7	0.81	0.68–0.96
	nabumetone	7,060	54	1,392	38.8	29.1–50.6	0.86	0.65–1.15
	naproxen	36,577	245	5,695	43.0	37.8–48.8	0.93	0.79–1.09
	rofecoxib	26,247	398	5,303	75.0	67.9–82.8	1.66	1.45–1.91
	valdecoxib	7,624	45	1,470	30.6	22.3–41.0	0.67	0.50–0.92
**AMI**^**‡**^**/** **Ischemic stroke**	Overall	268,113	2,822	48,176	58.6	56.4–60.8		
	celecoxib	66,316	888	15,807	56.2	52.5–60.0	1.07	0.96–1.20
	diclofenac	18,593	183	3,360	54.5	46.9–63.0	0.97	0.82–1.14
	etodolac	2,807	33	531	62.2	42.8–87.3	1.13	0.79–1.60
	ibuprofen	69,779	538	8,623	62.4	57.2–67.9	*Reference drug*	
	indomethacin	7,651	93	828	112.4	90.7–137.6	1.72	1.38–2.15
	meloxicam	25,459	225	5,245	42.9	37.5–48.9	0.78	0.67–0.92
	nabumetone	7,060	59	1,386	42.6	32.4–54.9	0.78	0.59–1.02
	naproxen	36,577	311	5,670	54.9	48.9–61.3	0.94	0.81–1.08
	rofecoxib	26,247	417	5,264	79.2	71.8–87.2	1.44	1.27–1.64
	valdecoxib	7,624	75	1,463	51.3	40.3–64.3	0.91	0.72–1.16
**GIB/ICH**^**§**^	Overall	268,087	2,620	48,225	54.3	52.3–56.5		
	celecoxib	66,310	736	15,846	46.4	43.2–49.9	1.11	0.98–1.26
	diclofenac	18,592	207	3,354	61.7	53.6–70.7	1.39	1.17–1.64
	etodolac	2,806	29	533	54.5	36.5–78.2	1.25	0.86–1.83
	ibuprofen	69,775	416	8,635	48.2	43.7–53.0	*Reference drug*	
	indomethacin	7,651	108	829	130.3	106.9–157.4	2.62	2.12–3.23
	meloxicam	25,455	246	5,252	46.8	41.2–53.1	1.08	0.92–1.27
	nabumetone	7,060	45	1,385	32.5	23.7–43.5	0.75	0.55–1.02
	naproxen	36,576	365	5,668	64.4	58.0–71.4	1.41	1.22–1.62
	rofecoxib	26,240	411	5,259	78.1	70.8–86.1	1.79	1.56–2.06
	valdecoxib	7,622	57	1,463	39.0	29.5–50.5	0.88	0.67–1.16

*p-ys: person-years.

^†^CI: confidence interval.

^**‡**^AMI: acute myocardial infarction.

^§^GIB/ICH: gastrointestinal bleeding/intracranial hemorrhage.

### Adjusted hazard ratios of outcomes

**Fig 1:**
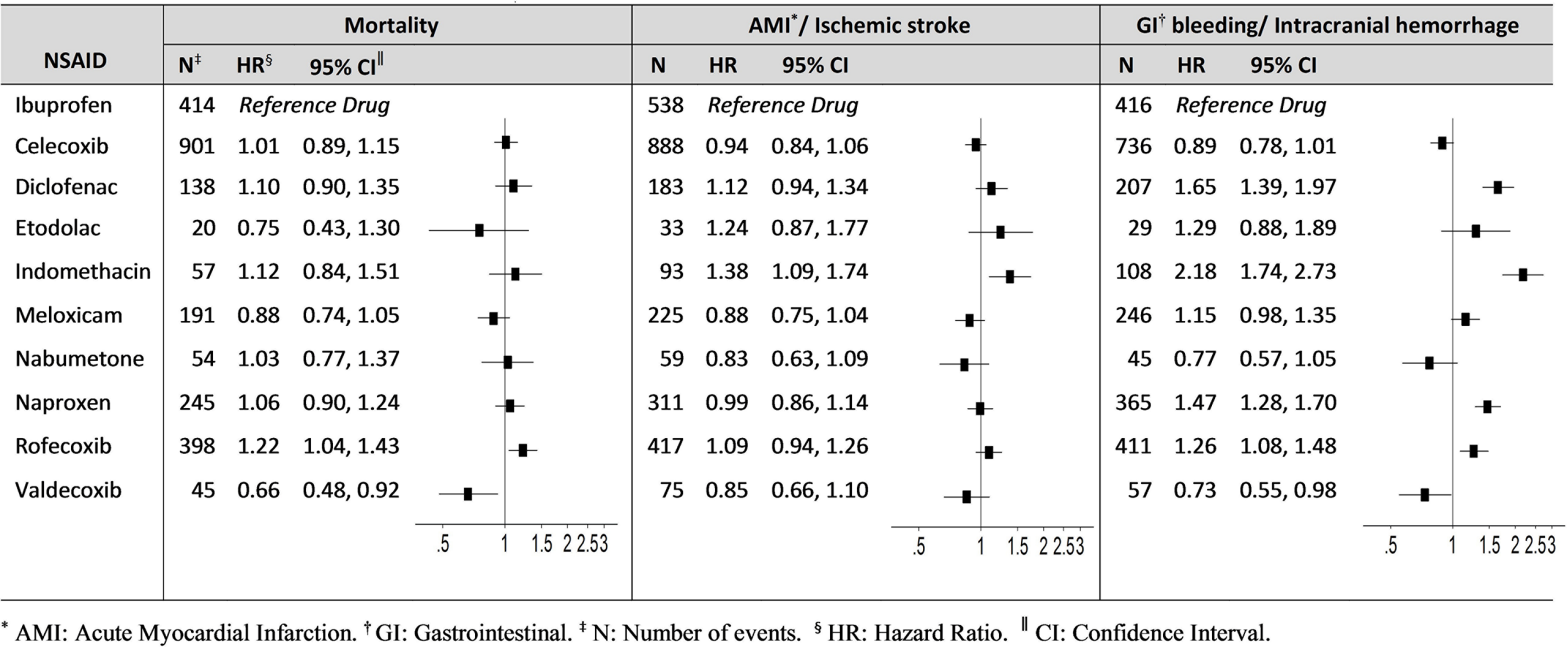
Primary analysis: Adjusted hazard ratios of outcomes by NSAID exposure group.

**Fig 1** presents the propensity score-adjusted hazard ratios (HRs) of each NSAID vs. ibuprofen in the primary analysis. Compared with ibuprofen, rofecoxib (HR = 1.22; 95% CI: 1.04, 1.43) and valdecoxib (HR = 0.66; 95% CI: 0.48, 0.92) were associated with increased and reduced hazard for all-cause mortality, respectively. Indomethacin (HR = 1.38; 95% CI: 1.09, 1.74) was associated with an increased hazard for AMI/ischemic stroke. For GIB/ICH, indomethacin (HR = 2.18; 95% CI: 1.74, 2.73), diclofenac (HR = 1.65; 95% CI: 1.39, 1.97), naproxen (HR = 1.47; 95% CI: 1.28, 1.70), and rofecoxib (HR = 1.26; 95% CI: 1.08, 1.48) were associated with increased hazards, and valdecoxib (HR = 0.73; 95% CI: 0.55, 0.98) was associated with a reduced hazard. The results of the secondary analysis stratified by a) age (detailed results presented in **S6 Table and S2 Fig**), b) sex (**S6 Table and S3 Fig**), and c) concomitancy triggering drug (**S6 Table and S4 Fig**) were not substantially different. The propensity score-adjusted hazard ratios of each NSAID from the sensitivity analyses are presented in **S5 Fig**. The results were similar to the primary analysis results.

**Fig 2:**
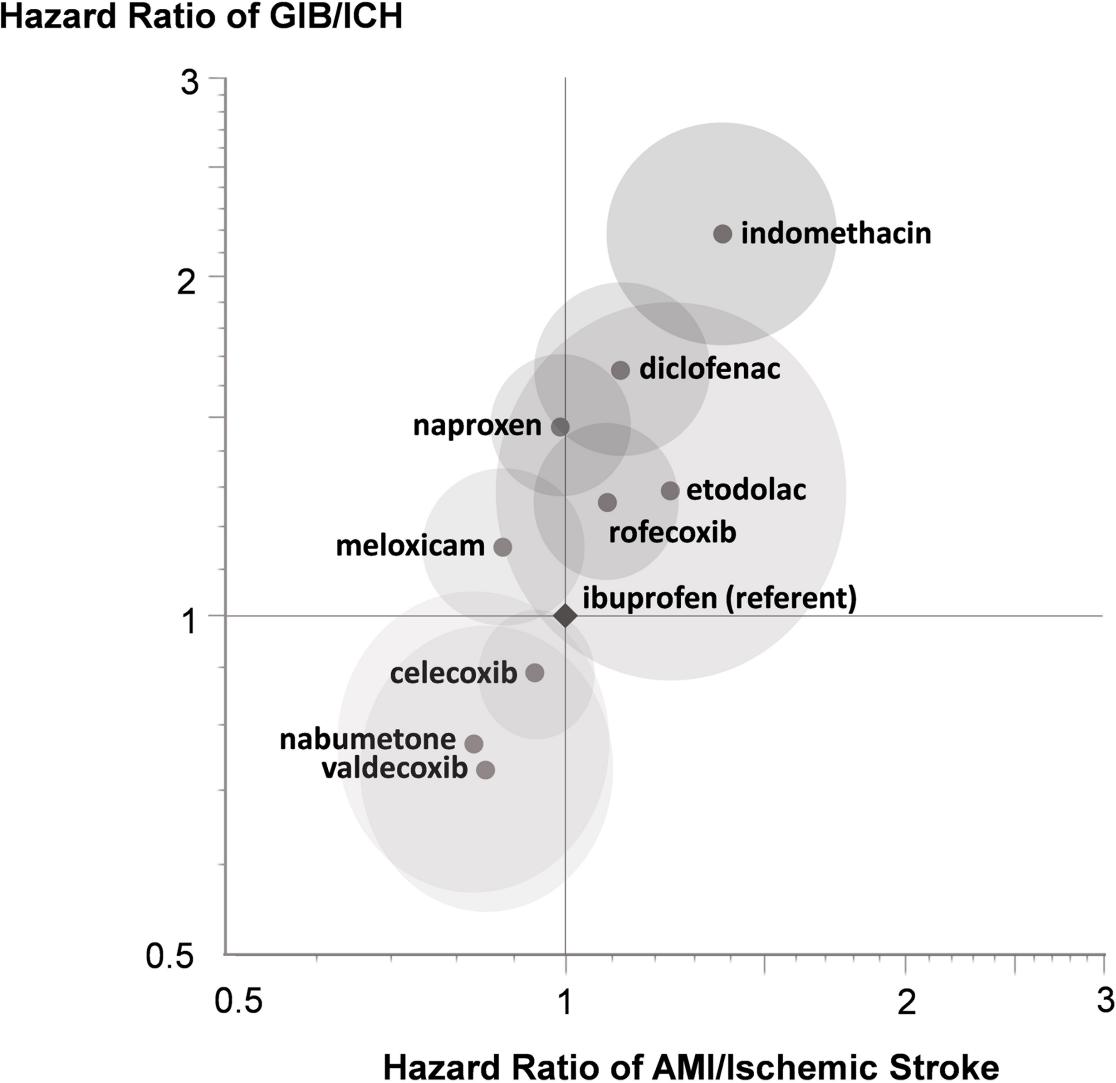
Primary analysis: Adjusted hazard ratios (with 95% confidence intervals) of AMI/Ischemic stroke and GIB/ICH of NSAIDs when concomitantly used with clopidogrel. AMI: acute myocardial infarction. GIB: gastrointestinal bleeding. ICH: intracranial hemorrhage. The natural logarithmic scale was used on the horizontal and the vertical axes. The center of an ellipse represents the point estimate of hazard ratio. The major axis and the minor axis of the ellipse represent 95% confidence intervals.

**Fig 2** displays the hazard ratios and 95% confidence intervals of GIB/ICH vs. AMI/ischemic stroke of NSAIDs when concomitantly used with clopidogrel. Celecoxib, nabumetone, and valdecoxib were associated with reduced hazards for both composite outcomes than ibuprofen, and rofecoxib, etodolac, diclofenac, and indomethacin were associated with increased hazards for both outcomes. NSAIDs that had higher thrombotic risks than ibuprofen also showed a tendency of having higher hemorrhagic risks.

## Discussion

This study examined the comparative safety of individual NSAIDs relative to ibuprofen in patients receiving clopidogrel. We found that in the presence of clopidogrel, the differences in the bleeding risk, measured as a composite outcome of GIB and ICH, among individual NSAIDs varied more markedly than in the thrombotic risk, measured as a composite outcome of AMI and ischemic stroke. Indomethacin, diclofenac, naproxen, and rofecoxib showed a statistically significantly higher bleeding risk than ibuprofen, while valdecoxib showed a significantly reduced bleeding risk. In contrast, no statistically significant differences in the thrombotic risk were found, except for indomethacin that showed significantly increased risk. In all-cause mortality, rofecoxib and valdecoxib were associated with a significantly increased and reduced risk, respectively. In addition, unlike what one might anticipate from the results of prior studies in patients not receiving clopidogrel [47–48], our results suggest that the thrombotic risks might not be inversely related to the bleeding risks of NSAIDs in patients also using clopidogrel. In particular, the combined results of randomized controlled trials in the absence of clopidogrel have suggested that the cardiovascular risk and GI risk have an inverse relationship, which is thought to be related to the degree of COX-2 or COX-1 selectivity [47–48]. Our results, however, suggest the absence of an inverse relationship in persons taking clopidogrel, as illustrated in **Fig 2**.

The underlying mechanisms for these results have remained to be elucidated. Yet, this observational study provides epidemiological insights suggesting that the known safety profiles of individual NSAIDs might differ in the presence of clopidogrel. For example, the molecular mechanisms that underlie differences in the safety profiles of distinct NSAIDs (related to factors such as COX-1 or COX-2 selectivity, inhibition of CYP enzymes or platelet aggregation) or their potencies might be different when NSAIDs are given concomitantly with clopidogrel. Drug-drug interactions might exist at the functional level (such as reduction of the cardiovascular risk by clopidogrel) and potentially also at the molecular level (such as alterations of the metabolism of some, but not all, NSAIDs due to CYP inhibition). Further studies are needed to shed light on the underlying mechanisms related to potential interactions between individual NSAIDs and clopidogrel. In addition, the risk of the individual components of our composite outcomes will need further investigation.

A direct comparison of our findings with the results of prior observational studies in patients receiving clopidogrel plus NSAIDs is difficult because of differences in the data, study population, definitions of exposure and outcomes, and methods, among others. For instance, a recent cohort study that analyzed the Danish nationwide administrative data reported increased risks of a composite cardiovascular outcomes (cardiovascular death, nonfatal recurrent MI, and stroke) and bleeding associated with the concomitant use of clopidogrel and NSAIDs (rofecoxib, celecoxib, diclofenac, ibuprofen, and naproxen) after MI, vs. non-NSAID-exposure as the referent [49]. They found that the risks of serious bleeding and cardiovascular events were higher with the use of any NSAID compared to non-use of NSAIDs among clopidogrel users. In that Danish study, the serious bleeding risk was higher for diclofenac, naproxen, and celecoxib than for ibuprofen, and the cardiovascular risk was lower for rofecoxib, celecoxib, diclofenac, and naproxen than for ibuprofen. The findings in the Danish study that the highest cardiovascular risk was with ibuprofen, and that bleeding risk was higher for celecoxib than for ibuprofen are not consistent with our results. Potentially important differences between two studies include that the prior study was restricted to the patients who were admitted with first-time MI and alive 30 days after discharge; the definitions of outcomes of interest differed; and that our study had a much larger number of users of clopidogrel and NSAIDs.

Our study has several important strengths. First, we studied a large, vulnerable population. Medicaid covers nearly 70 million people nationwide, or 1 in 5 Americans. This large, vulnerable population is therefore important to study in its own right. Second, this study compared individual NSAIDs, which helps reduce the potential for confounding by indication that can arise in the comparison of NSAID users vs. non-users. Third, the algorithms we used to ascertain outcomes of interest have been found to perform well. Lastly, this study reflects safety profile of NSAIDs in a real world setting, unlike clinical trials that are conducted under strictly regulated protocols generally among highly selected populations at specialized centers and much smaller numbers of patients.

This study also has limitations. First, data on genetic factors related to the CYP enzymes responsible for clopidogrel metabolism were unavailable in our dataset. Second, since we used administrative claims data, complete information on drug ingestion (including P.R.N., i.e., administration as needed, or other intermittent use of drug), lifestyle, or health behaviors (including smoking) was unavailable. It is notable, however, randomized clinical trials in ambulatory settings often have incomplete or unreliable drug adherence data [50] as well. We controlled for tobacco use as a pre-specified covariate (**S4 Table**), as a prior study documented that ICD-9 codes for tobacco use had a specificity of 100%, a sensitivity of 32%, and showed little evidence of documentation bias [51]. Third, this study does not provide information on the effect of dose on risk or risk of NSAID use vs. non-use. Future research that examines dose-response relationship will enable comparison of dose-dependent risk. Also, because our study compared the outcomes of interest among NSAIDs, the estimated risks in this study does not represent risks of NSAID users compared to NSAID non-users, in the presence of clopidogrel. Fourth, although we controlled for many potential confounders, residual confounding may remain, as is the case with observational pharmacoepidemiologic studies in general. For example, we controlled for prescription aspirin use in the propensity score model, but we may not have captured all aspirin use because aspirin is available over-the-counter. The proportion of the subjects in this study with a recorded prescription for aspirin dispensed in the 60-day period before cohort entry was 13–23%, depending on NSAID. Given that aspirin use may influence comparative risk of NSAIDs, it will be important to control for over-the-counter aspirin use in future research provided that the data are available. Also, selection bias or channeling effect may be part of residual confounding. This study employed statistical methods to control for these potential confounders as much as possible, but we cannot rule out the possibility of residual confounding. Fifth, our results were obtained from the Medicaid enrollees, who tend to be vulnerable. Therefore, magnitude of the associations identified here may not be generalizable to other populations.

## Conclusions

In users of clopidogrel, the differences in the bleeding risk (GIB/ICH) among individual NSAIDs relative to ibuprofen were more pronounced than in the thrombotic risk (AMI/ischemic stroke). Bleeding risks and thrombotic risks among individual NSAIDs did not appear to be inversely related to each other, unlike the results from prior studies conducted in the absence of clopidogrel. Although these findings are not definitive, bleeding risk might need a relatively greater attention in the NSAID therapy among clopidogrel users, weighing anticipated benefits and risks. Further studies are needed to better understand the comparative safety of NSAIDs in the concomitant use of clopidogrel and underlying mechanisms to help clinical decision-making for a better NSAID choice, and thereby proactively reduce preventable serious adverse outcomes.

## Supporting information

S1 Fig Identification of study cohorts and application of inclusion and exclusion criteria. (TIF)

S2 Fig Secondary analysis stratified by age: Adjusted hazard ratios (with 95% confidence intervals) of AMI/Ischemic Stroke and GIB/ICH of NSAIDs when concomitantly used with clopidogrel. A. 18 ≤ Age < 65 years.
B. 65 ≤ Age ≤ 100 years.
(TIF)

S3 Fig Secondary analysis stratified by sex: Adjusted hazard ratios (with 95% confidence intervals) of AMI/Ischemic Stroke and GIB/ICH of NSAIDs when concomitantly used with clopidogrel. A. Male.
B. Female.
(TIF)

S4 Fig Secondary analysis stratified by concomitancy triggering drug: Adjusted hazard ratios (with 95% confidence intervals) of AMI/Ischemic Stroke and GIB/ICH of NSAIDs when concomitantly used with clopidogrel. A. NSAID-triggered group.
B. Clopidogrel-triggered and combination-triggered group.
(TIF)

S5 Fig Sensitivity analysis: Adjusted hazard ratios of outcomes by NSAID exposure group. A. Whole cohort with the follow-up time up to 180 days after cohort entry date.
B. Excluding potential incomplete-data patients, without a restriction on the follow-up time.
(TIF)

S1 Table Pre-specified covariates included in the propensity score model. (DOCX)

S2 Table Specifications used in the high-dimensional propensity score method. (DOCX)

S3 Table Covariates empirically identified by the high-dimensional propensity score method. (DOCX)

S4 Table Characteristics of clopidogrel users by NSAID exposure group. (DOCX)

S5 Table Sensitivity analysis: Unadjusted incidence rates of outcomes by NSAID exposure group. A. Whole cohort with the follow-up time up to 180 days after cohort entry date.
B. Excluding potential incomplete-data patients, without a restriction on the follow-up time.
(DOCX)

S6 Table Secondary analysis stratified by age, sex, and concomitancy triggering drug: Adjusted hazard ratios of outcomes by NSAID exposure group. (DOCX)
