# Correction: Nonsteroidal anti-inflammatory drug choice and adverse outcomes in clopidogrel users: A retrospective cohort study

**DOI:** 10.1371/journal.pone.0200982

**Published:** 2018-07-16

**Authors:** Young Hee Nam, Colleen M. Brensinger, Warren B. Bilker, Charles E. Leonard, Scott E. Kasner, Tilo Grosser, Xuanwen Li, Sean Hennessy

## Notice of republication

Incorrect versions of Supporting Information files S1 Fig, S4 Table, and S6 Table were published in error. This article was republished on July 06, 2018 to correct for these errors. Please download this article again to view the correct version.

## References

[1] Nam YH, Brensinger CM, Bilker WB, Leonard CE, Kasner SE, Grosser T, et al (2018) Nonsteroidal anti-inflammatory drug choice and adverse outcomes in clopidogrel users: A retrospective cohort study. PLoS ONE 13(3): e0193800 10.1371/journal.pone.0193800 29538453 PMC5851628

