# Supplementary figures and images for: Nonsteroidal anti-inflammatory drug choice and adverse outcomes in clopidogrel users: A retrospective cohort study
Source: PLoS One. 2018 Mar 14;13(3):e0193800. doi: 10.1371/journal.pone.0193800 (PMC5851628; doi:10.1371/journal.pone.0193800)

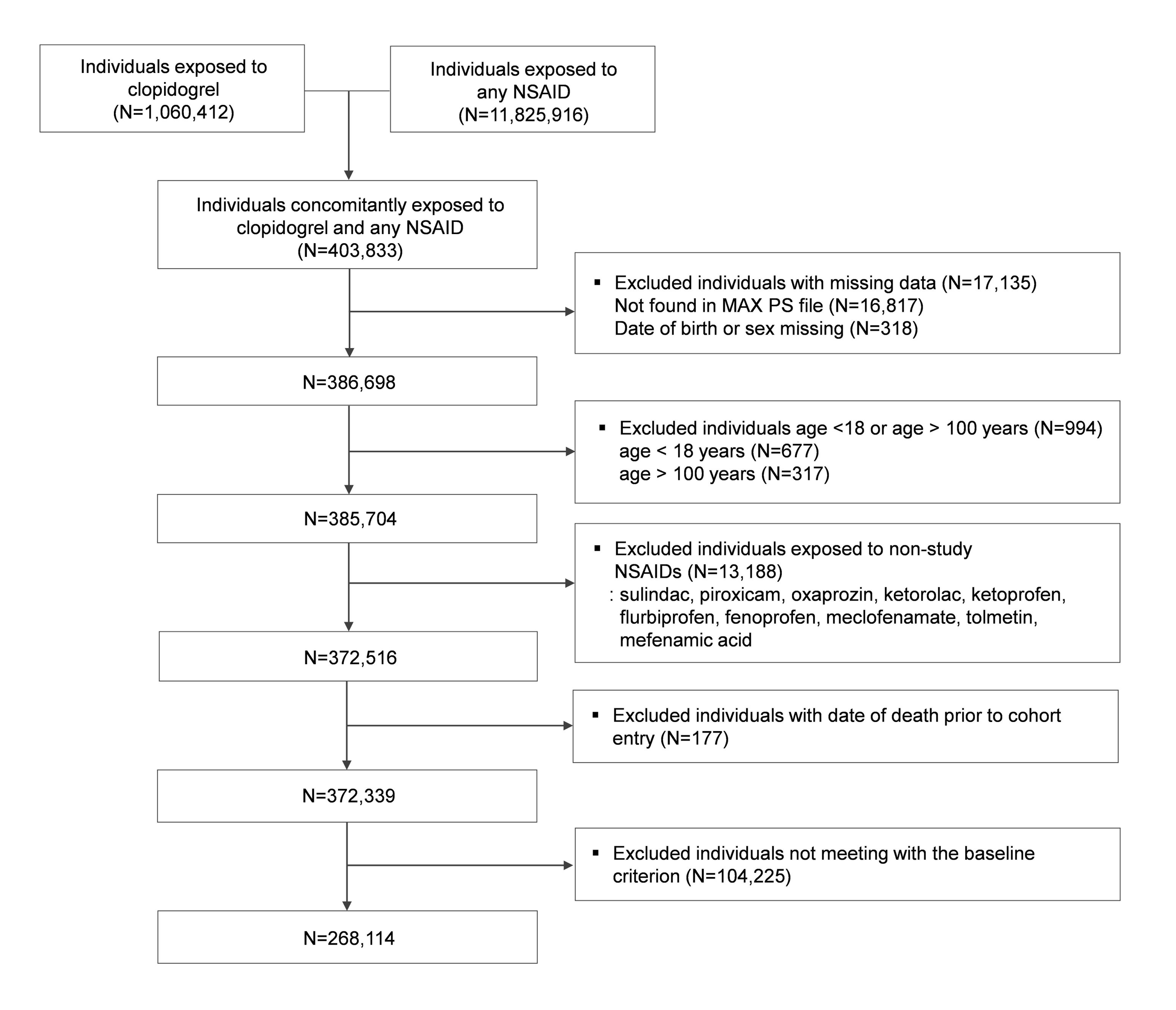

Supplement: S1 Fig — (TIF) [file pone.0193800.s001.tif]
