# Supplementary material for: Nonsteroidal anti-inflammatory drug choice and adverse outcomes in clopidogrel users: A retrospective cohort study
Source: PLoS One. 2018 Mar 14;13(3):e0193800. doi: 10.1371/journal.pone.0193800 (PMC5851628; doi:10.1371/journal.pone.0193800)
Supplement: S1 Table — (DOCX) [file pone.0193800.s006.docx]

**S1 Table. Pre-specified covariates included in the propensity score model**

| **Category** | **Covariate** | **N < 10*** | **WCSD > 0.1** |
| --- | --- | --- | --- |
| Demographic factors | Age at cohort entry |  | Yes |
|  | Sex |  | Yes |
|  | Race |  | Yes |
|  | Nursing home residence ever during the baseline time |  | Yes |
|  | Medicaid-Medicare dual-eligible |  | Yes |
|  | State of residence |  | Yes |
| Healthcare utilization factors during the one-year baseline time | Number of circulatory system ED visits |  | Yes |
|  | Number of non- circulatory system ED visits |  | Yes |
|  | Number of circulatory system hospitalizations |  | Yes |
|  | Number of non-circulatory system hospitalizations |  | Yes |
|  | Number of unique prescriptions |  | Yes |
|  | Number of circulatory system outpatient visits |  | Yes |
|  | Number of non-circulatory system outpatient visits |  | Yes |
|  | Number of prescriptions |  | Yes |
|  | Number of unique prescriptions |  | Yes |
|  | Number of inpatient ICD-9 diagnosis codes |  | Yes |
|  | Number of unique inpatient ICD-9 diagnosis |  | Yes |
|  | Number of inpatient ICD-9 procedure codes |  | Yes |
|  | Number of unique inpatient ICD-9 procedure codes |  | Yes |
|  | Number of inpatient CPT-4/HCPCS procedure codes |  | Yes |
|  | Number of unique inpatient CPT-4/HCPCS procedure codes |  | Yes |
|  | Number of outpatient ICD-9 diagnosis codes |  | Yes |
|  | Number of unique outpatient ICD-9 diagnoses |  | Yes |
|  | Number of outpatient ICD-9 procedure codes |  | Yes |
|  | Number of unique outpatient ICD-9 procedure codes |  | Yes |
|  | Number of outpatient CPT-4/HCPCS procedure codes |  | Yes |
|  | Number of unique outpatient CPT-4/HCPCS procedure codes |  | Yes |
|  | Number of other ICD-9 diagnosis codes |  | Yes |
|  | Number of unique other ICD-9 diagnosis codes |  | Yes |
|  | Number of other ICD-9 procedure codes |  |  |
|  | Number of unique other ICD-9 procedure codes |  |  |
| Diseases during the one-year baseline time | Acute respiratory infection |  |  |
|  | Fever |  |  |
|  | Gingival and periodontal disease | Yes |  |
|  | Infectious and parasitic diseases: herpes simplex | Yes |  |
|  | Infectious and parasitic diseases: Helicobacter pylori | Yes |  |
|  | Infection: other serious |  | Yes |
|  | Pneumonia |  |  |
|  | Urinary tract infection |  | Yes |
|  | Infectious and parasitic diseases: cytomegaloviral | Yes |  |
|  | Infectious and parasitic diseases: other |  | Yes |
|  | Alcohol abuse |  |  |
|  | Angina pectoris |  | Yes |
|  | Artery disease |  | Yes |
|  | Asthma/COPD/emphysema |  | Yes |
|  | Atrial fibrillation |  | Yes |
|  | Cerebrovascular disease: ischemic stroke |  | Yes |
|  | Cerebrovascular disease: transient cerebral ischemia |  | Yes |
|  | Cerebrovascular disease: hemorrhage |  |  |
|  | Cerebrovascular disease: other |  | Yes |
|  | Cancer |  | Yes |
|  | Cardiovascular system symptoms |  | Yes |
|  | Circulatory system disease: other |  | Yes |
|  | Conduction disorders |  |  |
|  | Congenital anomalies of heart |  |  |
|  | Diabetes mellitus |  | Yes |
|  | Heart failure |  | Yes |
|  | HIV/AIDS |  |  |
|  | Hypertension |  | Yes |
|  | Ischemic heart disease |  | Yes |
|  | Lipoid metabolism disorder |  | Yes |
|  | Mental disorder: depression |  | Yes |
|  | Mental disorder: other |  | Yes |
|  | Myocardial infarction: acute |  |  |
|  | Myocardial infarction: old |  | Yes |
|  | Nervous system disease: disorders of the eye and adnexa |  | Yes |
|  | Nervous system disease (central): hereditary and degenerative |  | Yes |
|  | Nervous system disease (central): inflammatory disease | Yes |  |
|  | Nervous system disease (central): other |  | Yes |
|  | Nervous system disease (peripheral) |  | Yes |
|  | Obesity |  | Yes |
|  | Pulmonary congestion and hypostasis |  | Yes |
|  | Pacemaker/ICD |  |  |
|  | Renal failure: acute |  | Yes |
|  | Renal disease: chronic |  | Yes |
|  | Renal disease: other |  | Yes |
|  | Stent placement |  |  |
|  | Substance abuse |  |  |
|  | Tobacco use |  | Yes |
|  | Cardiac dysrhythmias |  | Yes |
|  | Hypothyroidism |  | Yes |
|  | Liver diseases |  | Yes |
|  | Osteoarthritis |  | Yes |
|  | Rheumatoid arthritis |  | Yes |
|  | AMI |  |  |
|  | GI bleeding |  |  |
|  | Ischemic stroke |  |  |
|  | Non-traumatic intracranial hemorrhage |  |  |
| Drugs during the one-year baseline time | CYP1A2 inhibitors |  | Yes |
|  | CYP2B6 inhibitors | Yes |  |
|  | CYP2C19 inhibitors |  | Yes |
|  | CYP2C9 inhibitors |  | Yes |
|  | CYP3A45 inhibitors |  | Yes |
|  | CYP1A2 inducers |  | Yes |
|  | CYP2B6 inducers |  |  |
|  | CYP2C19 inducers |  |  |
|  | CYP2C9 inducers |  |  |
|  | CYP3A45 inducers |  |  |
|  | Antidepressants: other |  |  |
|  | H2-receptor antagonists |  | Yes |
|  | Potassium supplements |  | Yes |
|  | Antidepressants: monoamine oxidase inhibitors | Yes |  |
|  | Oral contraceptives |  |  |
|  | Proton pump inhibitors |  | Yes |
|  | Antidepressants: serotonin and norepinephrine reuptake inhibitors |  |  |
|  | Antidepressants: selective serotonin reuptake inhibitors |  | Yes |
|  | Thiazolidinediones |  |  |
|  | Aspirin |  | Yes |
|  | Benzisoxazoles |  |  |
|  | Dibenzazepines |  | Yes |
|  | Dipyridamole |  |  |
|  | Estrogens |  |  |
|  | Influenza vaccination |  | Yes |
|  | Non-study NSAIDs |  |  |
|  | Phenothiazines |  |  |
|  | Phenylbutylpiperidines |  |  |
|  | Quinolinones |  |  |
|  | Antidepressants: tetracyclic |  |  |
|  | Thiazides diuretics |  |  |
|  | Thioxanthines | Yes |  |
|  | Antidepressants: tricyclic |  |  |
|  | Warfarin |  | Yes |
|  | Anti-infectives |  | Yes |
|  | Anti-infectives in 7 days prior to cohort entry |  |  |
|  | Calcium channel blockers |  | Yes |
|  | Antiadrenergic agents |  | Yes |
|  | Antialcohol agents | Yes |  |
|  | Antiarrhythmic agents |  |  |
|  | Anticoagulants |  | Yes |
|  | Anticonvulsants |  |  |
|  | Antidepressants |  | Yes |
|  | Antidiabetic agents: insulin |  | Yes |
|  | Antidiabetic agents: non-insulin |  | Yes |
|  | Agents for migraine |  |  |
|  | Antiobesity agents | Yes |  |
|  | Antiplatelet agents excluding clopidogrel |  |  |
|  | Antipsychotics |  | Yes |
|  | Antiretroviral agents |  |  |
|  | Beta-adrenergic agents and alpha/beta-adrenergic blocking agents |  | Yes |
|  | Bronchodilators/inhaled corticosteroids |  | Yes |
|  | Diuretics: loop |  |  |
|  | Diuretics: other |  |  |
|  | Fibrates |  |  |
|  | Inotropic agents |  | Yes |
|  | Leukotriene formation inhibitors and leukotriene receptor antagonists |  |  |
|  | Ranolazine | Yes |  |
|  | Renin angiotensin system antagonists |  | Yes |
|  | Statins |  | Yes |
|  | Thyroid hormones |  |  |
|  | Varenicline | Yes |  |
|  | Vasodilators |  | Yes |

ED: emergency department. ICD-9: International Classification of Diseases 9th Revision. CPT-4: Current Procedural Terminology 4th Edition. HCPCS: Healthcare Common Procedure Coding System. ICD: Implantable Cardioverter Defibrillator. WCSD: weight conditional standardized difference.

* Infrequently-occurring covariates excluded from the propensity score model due to concerns of potential model instability.
