# Supplementary material for: Nonsteroidal anti-inflammatory drug choice and adverse outcomes in clopidogrel users: A retrospective cohort study
Source: PLoS One. 2018 Mar 14;13(3):e0193800. doi: 10.1371/journal.pone.0193800 (PMC5851628; doi:10.1371/journal.pone.0193800)
Supplement: S2 Table — (DOCX) [file pone.0193800.s007.docx]

**S2 Table. Specifications used in the high-dimensional propensity score method**

| Data dimensions (*p*) | Inpatient^a^ ICD-9 diagnoses |
| --- | --- |
|  | Inpatient^a^ ICD-9 procedures |
|  | Inpatient^a^ CPT/HCPCS procedures |
|  | Outpatient^b^ ICD-9 diagnoses |
|  | Outpatient^b^ ICD-9 procedures |
|  | Outpatient^b^ CPT/HCPCS procedures |
|  | Other Setting^c^ ICD-9 diagnoses |
|  | Other setting^c^ ICD-9 procedures |
|  | Outpatient* medication^d^ active ingredients |
| Granularity of *p* | 3 digits for ICD-9 diagnoses, 2 digits for ICD-9 procedures, 5 digits for CPT, 5 alphanumeric characters for HCPCS, and Cerner Multum Lexicon-defined active ingredient for drugs |
| Covariates empirically identified (*n*), per *p*, ranked in descending order by prevalence | *n* = 200 |
| Method of covariate prioritization | Bross bias formula^e^ |
| Covariates empirically selected (*k*), across *p*, subsequent to prioritization | *k* = 500, plus pre-specified covariates |
| Zero-cell correction screening | No, given adequate number of exposed outcomes |

ICD-9: International Classification of Diseases 9th Revision. CPT: Current Procedural Terminology. HCPCS: Healthcare Common Procedure Coding System.

^a^Inpatient: Claims arising from Medicaid Analytic Extract Inpatient file and Medicare Provider Analysis and Review file (limited to short stay and long stay hospitalization claims).

^b^Outpatient: Claims arising from Medicaid Analytic Extract Other Therapy file, Medicare Carrier file, and Medicare Outpatient Standard Analytic file.

^c^Other setting: Claims arising from Medicaid Analytic Extract Long Term Care file and Medicare Provider Analysis and Review file (limited to skilled nursing facility claims).

^d^Medication: Claims arising from Medicaid Analytic Extract Prescription file and Medicare Part D Event file.

^e^Bross bias formula: Bross ID. Spurious effects from an extraneous variable. *J Chronic Dis*. 1966;19(6):637-647.

*Inpatient medications are not available in the Centers for Medicare and Medicaid Services data.
