# Supplementary material for: Nonsteroidal anti-inflammatory drug choice and adverse outcomes in clopidogrel users: A retrospective cohort study
Source: PLoS One. 2018 Mar 14;13(3):e0193800. doi: 10.1371/journal.pone.0193800 (PMC5851628; doi:10.1371/journal.pone.0193800)
Supplement: S3 Table — (DOCX) [file pone.0193800.s008.docx]

**S3 Table. Covariates empirically identified by the high-dimensional propensity score method**

| **Data Dimension** | **Code** | **Code Description** | **N < 10*** |
| --- | --- | --- | --- |
| Drug | Drug-specific NDCs | acetaminophen |  |
| Drug | Drug-specific NDCs | albuterol |  |
| Drug | Drug-specific NDCs | albuterol sulfate |  |
| Drug | Drug-specific NDCs | alendronate (as alendronate sodium) |  |
| Drug | Drug-specific NDCs | allopurinol |  |
| Drug | Drug-specific NDCs | aluminum hydroxide |  |
| Drug | Drug-specific NDCs | amiodarone hydrochloride |  |
| Drug | Drug-specific NDCs | amlodipine besylate |  |
| Drug | Drug-specific NDCs | ammonium lactate |  |
| Drug | Drug-specific NDCs | amoxicillin (as trihydrate) |  |
| Drug | Drug-specific NDCs | amylase |  |
| Drug | Drug-specific NDCs | aspirin |  |
| Drug | Drug-specific NDCs | atorvastatin |  |
| Drug | Drug-specific NDCs | atropine sulfate |  |
| Drug | Drug-specific NDCs | bacitracin |  |
| Drug | Drug-specific NDCs | betamethasone dipropionate |  |
| Drug | Drug-specific NDCs | brimonidine tartrate |  |
| Drug | Drug-specific NDCs | budesonide |  |
| Drug | Drug-specific NDCs | bupropion hydrochloride |  |
| Drug | Drug-specific NDCs | butalbital |  |
| Drug | Drug-specific NDCs | caffeine |  |
| Drug | Drug-specific NDCs | calcium (as carbonate) |  |
| Drug | Drug-specific NDCs | calcium (as phosphate) | Yes |
| Drug | Drug-specific NDCs | carisoprodol |  |
| Drug | Drug-specific NDCs | carvedilol |  |
| Drug | Drug-specific NDCs | cephalexin (as monohydrate) |  |
| Drug | Drug-specific NDCs | cetirizine hydrochloride |  |
| Drug | Drug-specific NDCs | clarithromycin |  |
| Drug | Drug-specific NDCs | clobetasol propionate |  |
| Drug | Drug-specific NDCs | clonidine hydrochloride |  |
| Drug | Drug-specific NDCs | clotrimazole |  |
| Drug | Drug-specific NDCs | codeine phosphate |  |
| Drug | Drug-specific NDCs | colchicine |  |
| Drug | Drug-specific NDCs | conjugated estrogens |  |
| Drug | Drug-specific NDCs | cyclobenzaprine hydrochloride |  |
| Drug | Drug-specific NDCs | dexamethasone |  |
| Drug | Drug-specific NDCs | dicyclomine hydrochloride |  |
| Drug | Drug-specific NDCs | digoxin |  |
| Drug | Drug-specific NDCs | diltiazem hydrochloride |  |
| Drug | Drug-specific NDCs | diphenhydramine hydrochloride |  |
| Drug | Drug-specific NDCs | diphenoxylate hydrochloride |  |
| Drug | Drug-specific NDCs | docusate sodium |  |
| Drug | Drug-specific NDCs | donepezil hydrochloride |  |
| Drug | Drug-specific NDCs | doxazosin mesylate |  |
| Drug | Drug-specific NDCs | econazole nitrate |  |
| Drug | Drug-specific NDCs | enalapril maleate |  |
| Drug | Drug-specific NDCs | escitalopram oxalate |  |
| Drug | Drug-specific NDCs | esomeprazole (as magnesium trihydrate) |  |
| Drug | Drug-specific NDCs | ezetimibe |  |
| Drug | Drug-specific NDCs | famotidine |  |
| Drug | Drug-specific NDCs | fenofibrate |  |
| Drug | Drug-specific NDCs | ferrous sulfate |  |
| Drug | Drug-specific NDCs | fexofenadine hydrochloride |  |
| Drug | Drug-specific NDCs | fluconazole |  |
| Drug | Drug-specific NDCs | fluoxetine (as hydrochloride) |  |
| Drug | Drug-specific NDCs | folic acid |  |
| Drug | Drug-specific NDCs | furosemide |  |
| Drug | Drug-specific NDCs | gabapentin |  |
| Drug | Drug-specific NDCs | gatifloxacin |  |
| Drug | Drug-specific NDCs | gemfibrozil |  |
| Drug | Drug-specific NDCs | glimepiride |  |
| Drug | Drug-specific NDCs | guaifenesin |  |
| Drug | Drug-specific NDCs | hydralazine hydrochloride |  |
| Drug | Drug-specific NDCs | hydrochlorothiazide |  |
| Drug | Drug-specific NDCs | hydrocodone bitartrate |  |
| Drug | Drug-specific NDCs | hydrocortisone acetate |  |
| Drug | Drug-specific NDCs | hyoscyamine sulfate |  |
| Drug | Drug-specific NDCs | insulin aspart |  |
| Drug | Drug-specific NDCs | insulin glargine |  |
| Drug | Drug-specific NDCs | insulin nph human recombinant |  |
| Drug | Drug-specific NDCs | insulin regular human recombinant |  |
| Drug | Drug-specific NDCs | ipratropium bromide |  |
| Drug | Drug-specific NDCs | irbesartan |  |
| Drug | Drug-specific NDCs | isosorbide dinitrate |  |
| Drug | Drug-specific NDCs | isosorbide mononitrate |  |
| Drug | Drug-specific NDCs | lansoprazole |  |
| Drug | Drug-specific NDCs | levofloxacin |  |
| Drug | Drug-specific NDCs | levothyroxine sodium |  |
| Drug | Drug-specific NDCs | lidocaine |  |
| Drug | Drug-specific NDCs | lidocaine hydrochloride |  |
| Drug | Drug-specific NDCs | lipase |  |
| Drug | Drug-specific NDCs | lisinopril |  |
| Drug | Drug-specific NDCs | loratadine |  |
| Drug | Drug-specific NDCs | lorazepam |  |
| Drug | Drug-specific NDCs | lovastatin |  |
| Drug | Drug-specific NDCs | meclizine hydrochloride |  |
| Drug | Drug-specific NDCs | megestrol acetate |  |
| Drug | Drug-specific NDCs | metformin hydrochloride |  |
| Drug | Drug-specific NDCs | methylprednisolone |  |
| Drug | Drug-specific NDCs | metoclopramide hydrochloride |  |
| Drug | Drug-specific NDCs | metoprolol succinate |  |
| Drug | Drug-specific NDCs | metoprolol tartrate |  |
| Drug | Drug-specific NDCs | mirtazapine |  |
| Drug | Drug-specific NDCs | misoprostol |  |
| Drug | Drug-specific NDCs | mometasone furoate monohydrate |  |
| Drug | Drug-specific NDCs | moxifloxacin hydrochloride |  |
| Drug | Drug-specific NDCs | multiple vitamins |  |
| Drug | Drug-specific NDCs | mupirocin |  |
| Drug | Drug-specific NDCs | niacin |  |
| Drug | Drug-specific NDCs | nitroglycerin |  |
| Drug | Drug-specific NDCs | nystatin |  |
| Drug | Drug-specific NDCs | ocular lubricant drops, unspecified, pre |  |
| Drug | Drug-specific NDCs | olanzapine |  |
| Drug | Drug-specific NDCs | olmesartan medoxomil |  |
| Drug | Drug-specific NDCs | olopatadine hydrochloride |  |
| Drug | Drug-specific NDCs | omeprazole |  |
| Drug | Drug-specific NDCs | oxycodone hydrochloride |  |
| Drug | Drug-specific NDCs | pantoprazole (as pantoprazole sodium ses |  |
| Drug | Drug-specific NDCs | penicillin V potassium |  |
| Drug | Drug-specific NDCs | pentoxifylline |  |
| Drug | Drug-specific NDCs | phenazopyridine hydrochloride |  |
| Drug | Drug-specific NDCs | phenytoin sodium |  |
| Drug | Drug-specific NDCs | polyethylene glycol 3350 |  |
| Drug | Drug-specific NDCs | potassium chloride |  |
| Drug | Drug-specific NDCs | pravastatin sodium |  |
| Drug | Drug-specific NDCs | prednisone |  |
| Drug | Drug-specific NDCs | propoxyphene napsylate |  |
| Drug | Drug-specific NDCs | protease |  |
| Drug | Drug-specific NDCs | pseudoephedrine hydrochloride |  |
| Drug | Drug-specific NDCs | quetiapine fumarate |  |
| Drug | Drug-specific NDCs | quinapril hydrochloride |  |
| Drug | Drug-specific NDCs | quinine sulfate |  |
| Drug | Drug-specific NDCs | rabeprazole sodium |  |
| Drug | Drug-specific NDCs | raloxifene hydrochloride |  |
| Drug | Drug-specific NDCs | ranitidine hydrochloride |  |
| Drug | Drug-specific NDCs | risedronate sodium |  |
| Drug | Drug-specific NDCs | risperidone |  |
| Drug | Drug-specific NDCs | rosiglitazone maleate |  |
| Drug | Drug-specific NDCs | rosuvastatin calcium |  |
| Drug | Drug-specific NDCs | sertraline hydrochloride |  |
| Drug | Drug-specific NDCs | sildenafil citrate |  |
| Drug | Drug-specific NDCs | silver sulfadiazine |  |
| Drug | Drug-specific NDCs | simvastatin |  |
| Drug | Drug-specific NDCs | sodium chloride |  |
| Drug | Drug-specific NDCs | spironolactone |  |
| Drug | Drug-specific NDCs | sulfamethoxazole |  |
| Drug | Drug-specific NDCs | temazepam |  |
| Drug | Drug-specific NDCs | theophylline |  |
| Drug | Drug-specific NDCs | tiotropium |  |
| Drug | Drug-specific NDCs | tobramycin |  |
| Drug | Drug-specific NDCs | tramadol hydrochloride |  |
| Drug | Drug-specific NDCs | triamcinolone acetonide |  |
| Drug | Drug-specific NDCs | triamterene |  |
| Drug | Drug-specific NDCs | trimethoprim |  |
| Drug | Drug-specific NDCs | urea |  |
| Drug | Drug-specific NDCs | valsartan |  |
| Drug | Drug-specific NDCs | verapamil hydrochloride |  |
| Drug | Drug-specific NDCs | vitamin D |  |
| Drug | Drug-specific NDCs | warfarin sodium |  |
| Drug | Drug-specific NDCs | zolpidem tartrate |  |
| Inpatient ICD-9 Dx | 038 | septicemia |  |
| Inpatient ICD-9 Dx | 041 | bact inf in oth dis/nos |  |
| Inpatient ICD-9 Dx | 042 | human immuno virus dis | Yes |
| Inpatient ICD-9 Dx | 070 | viral hepatitis |  |
| Inpatient ICD-9 Dx | 112 | candidiasis |  |
| Inpatient ICD-9 Dx | 162 | mal neo trachea/lung | Yes |
| Inpatient ICD-9 Dx | 244 | acquired hypothyroidism |  |
| Inpatient ICD-9 Dx | 250 | diabetes mellitus |  |
| Inpatient ICD-9 Dx | 263 | prot-cal malnutr nec/nos |  |
| Inpatient ICD-9 Dx | 272 | dis of lipoid metabolism |  |
| Inpatient ICD-9 Dx | 274 | gout |  |
| Inpatient ICD-9 Dx | 275 | dis mineral metabolism |  |
| Inpatient ICD-9 Dx | 276 | fluid/electrolyte dis |  |
| Inpatient ICD-9 Dx | 280 | iron deficiency anemias |  |
| Inpatient ICD-9 Dx | 285 | anemia nec/nos |  |
| Inpatient ICD-9 Dx | 287 | purpura & oth hemor cond |  |
| Inpatient ICD-9 Dx | 290 | senile/presenile psychos |  |
| Inpatient ICD-9 Dx | 293 | transient org mental dis | Yes |
| Inpatient ICD-9 Dx | 294 | other organic psych cond |  |
| Inpatient ICD-9 Dx | 296 | affective psychoses |  |
| Inpatient ICD-9 Dx | 305 | nondependent drug abuse |  |
| Inpatient ICD-9 Dx | 311 | depressive disorder nec |  |
| Inpatient ICD-9 Dx | 331 | cerebral degeneration |  |
| Inpatient ICD-9 Dx | 332 | parkinson's disease |  |
| Inpatient ICD-9 Dx | 342 | hemiplegia |  |
| Inpatient ICD-9 Dx | 345 | epilepsy |  |
| Inpatient ICD-9 Dx | 348 | other brain conditions |  |
| Inpatient ICD-9 Dx | 357 | inflam/toxic neuropathy |  |
| Inpatient ICD-9 Dx | 362 | retinal disorders nec |  |
| Inpatient ICD-9 Dx | 386 | vertiginous syndromes |  |
| Inpatient ICD-9 Dx | 396 | mitral/aortic valve dis | Yes |
| Inpatient ICD-9 Dx | 397 | endocardial disease nec |  |
| Inpatient ICD-9 Dx | 398 | oth rheumatic heart dis | Yes |
| Inpatient ICD-9 Dx | 401 | essential hypertension |  |
| Inpatient ICD-9 Dx | 402 | hypertensive heart dis |  |
| Inpatient ICD-9 Dx | 403 | hypertensive renal dis |  |
| Inpatient ICD-9 Dx | 404 | hyperten heart/renal dis |  |
| Inpatient ICD-9 Dx | 410 | acute myocardial infarct |  |
| Inpatient ICD-9 Dx | 412 | old myocardial infarct |  |
| Inpatient ICD-9 Dx | 414 | oth chr ischemic hrt dis |  |
| Inpatient ICD-9 Dx | 416 | chr pulmonary heart dis |  |
| Inpatient ICD-9 Dx | 424 | oth endocardial disease |  |
| Inpatient ICD-9 Dx | 425 | cardiomyopathy |  |
| Inpatient ICD-9 Dx | 426 | conduction disorders |  |
| Inpatient ICD-9 Dx | 427 | cardiac dysrhythmias |  |
| Inpatient ICD-9 Dx | 428 | heart failure |  |
| Inpatient ICD-9 Dx | 434 | cerebral artery occlus |  |
| Inpatient ICD-9 Dx | 436 | cva |  |
| Inpatient ICD-9 Dx | 438 | late eff cerebrovasc dis |  |
| Inpatient ICD-9 Dx | 440 | atherosclerosis |  |
| Inpatient ICD-9 Dx | 443 | oth periph vascular dis |  |
| Inpatient ICD-9 Dx | 458 | hypotension |  |
| Inpatient ICD-9 Dx | 459 | oth circulatory disease |  |
| Inpatient ICD-9 Dx | 486 | pneumonia |  |
| Inpatient ICD-9 Dx | 491 | chronic bronchitis |  |
| Inpatient ICD-9 Dx | 496 | chr airway obstruct nec |  |
| Inpatient ICD-9 Dx | 507 | solid/liq pneumonitis |  |
| Inpatient ICD-9 Dx | 511 | pleurisy |  |
| Inpatient ICD-9 Dx | 518 | other lung diseases |  |
| Inpatient ICD-9 Dx | 530 | diseases of esophagus |  |
| Inpatient ICD-9 Dx | 531 | gastric ulcer |  |
| Inpatient ICD-9 Dx | 535 | gastritis and duodenitis |  |
| Inpatient ICD-9 Dx | 562 | diverticula of intestine |  |
| Inpatient ICD-9 Dx | 578 | gastrointestinal hemorr |  |
| Inpatient ICD-9 Dx | 583 | nephritis nos | Yes |
| Inpatient ICD-9 Dx | 584 | acute renal failure |  |
| Inpatient ICD-9 Dx | 585 | chronic renal failure |  |
| Inpatient ICD-9 Dx | 588 | impaired renal function | Yes |
| Inpatient ICD-9 Dx | 593 | oth renal & ureteral dis |  |
| Inpatient ICD-9 Dx | 599 | oth urinary tract disor |  |
| Inpatient ICD-9 Dx | 681 | cellulitis | Yes |
| Inpatient ICD-9 Dx | 682 | other cellulitis/abscess |  |
| Inpatient ICD-9 Dx | 707 | chronic ulcer of skin |  |
| Inpatient ICD-9 Dx | 714 | oth inflamm polyarthrop |  |
| Inpatient ICD-9 Dx | 715 | osteoarthrosis et al |  |
| Inpatient ICD-9 Dx | 730 | osteomyelitis | Yes |
| Inpatient ICD-9 Dx | 733 | oth bone & cartilage dis |  |
| Inpatient ICD-9 Dx | 780 | general symptoms |  |
| Inpatient ICD-9 Dx | 785 | cardiovascular sys symp |  |
| Inpatient ICD-9 Dx | 786 | resp sys/oth chest symp |  |
| Inpatient ICD-9 Dx | 787 | gi system symptoms |  |
| Inpatient ICD-9 Dx | 790 | abnormal blood findings |  |
| Inpatient ICD-9 Dx | 799 | oth ill-def morbid/mortl |  |
| Inpatient ICD-9 Dx | 820 | fracture neck of femur |  |
| Inpatient ICD-9 Dx | 995 | certain adverse eff nec |  |
| Inpatient ICD-9 Dx | 996 | replace & graft complic |  |
| Inpatient ICD-9 Dx | V10 | hx of malignant neoplasm |  |
| Inpatient ICD-9 Dx | V12 | hx of disease nec |  |
| Inpatient ICD-9 Dx | V15 | oth hx of health hazards |  |
| Inpatient ICD-9 Dx | V17 | fam hx-chr disabling dis |  |
| Inpatient ICD-9 Dx | V43 | organ replacement nec |  |
| Inpatient ICD-9 Dx | V44 | artificial opning status | Yes |
| Inpatient ICD-9 Dx | V45 | oth postsurgical states |  |
| Inpatient ICD-9 Dx | V49 | limb problem/problem nec |  |
| Inpatient ICD-9 Dx | V58 | encountr proc/aftrcr nec |  |
| Inpatient ICD-9 Px | 33 | other bronchial/lung ops |  |
| Inpatient ICD-9 Px | 34 | thorax ops except lung | Yes |
| Inpatient ICD-9 Px | 37 | other heart/pericard ops |  |
| Inpatient ICD-9 Px | 38 | vessel inc/excis/occlus |  |
| Inpatient ICD-9 Px | 39 | other ops on vessels |  |
| Inpatient ICD-9 Px | 43 | gastric incision/excis |  |
| Inpatient ICD-9 Px | 45 | intest incis/excis/anast |  |
| Inpatient ICD-9 Px | 79 | reduction fx/dislocation |  |
| Inpatient ICD-9 Px | 84 | other musculoskelet proc |  |
| Inpatient ICD-9 Px | 86 | skin & subq operations |  |
| Inpatient ICD-9 Px | 87 | diagnostic radiology |  |
| Inpatient ICD-9 Px | 88 | other dx radiology |  |
| Inpatient ICD-9 Px | 89 | interview/consult/exam |  |
| Inpatient ICD-9 Px | 93 | pt |  |
| Inpatient ICD-9 Px | 96 | non-op intubat & irrigat |  |
| Inpatient ICD-9 Px | 99 | other nonoperative proc |  |
| Inpatient CPT-4 Px | 62270 | spinal fluid tap, diagnostic | Yes |
| Outpatient ICD-9 Dx | 038 | septicemia |  |
| Outpatient ICD-9 Dx | 041 | bact inf in oth dis/nos |  |
| Outpatient ICD-9 Dx | 070 | viral hepatitis |  |
| Outpatient ICD-9 Dx | 110 | dermatophytosis |  |
| Outpatient ICD-9 Dx | 211 | oth benign neoplasm gi |  |
| Outpatient ICD-9 Dx | 239 | unspecified neoplasm |  |
| Outpatient ICD-9 Dx | 244 | acquired hypothyroidism |  |
| Outpatient ICD-9 Dx | 246 | oth disorders of thyroid |  |
| Outpatient ICD-9 Dx | 250 | diabetes mellitus |  |
| Outpatient ICD-9 Dx | 251 | oth pancreatic disorder |  |
| Outpatient ICD-9 Dx | 272 | dis of lipoid metabolism |  |
| Outpatient ICD-9 Dx | 274 | gout |  |
| Outpatient ICD-9 Dx | 275 | dis mineral metabolism |  |
| Outpatient ICD-9 Dx | 276 | fluid/electrolyte dis |  |
| Outpatient ICD-9 Dx | 280 | iron deficiency anemias |  |
| Outpatient ICD-9 Dx | 281 | other deficiency anemia |  |
| Outpatient ICD-9 Dx | 285 | anemia nec/nos |  |
| Outpatient ICD-9 Dx | 286 | coagulation defects |  |
| Outpatient ICD-9 Dx | 288 | wbc disorders |  |
| Outpatient ICD-9 Dx | 290 | senile/presenile psychos |  |
| Outpatient ICD-9 Dx | 294 | other organic psych cond |  |
| Outpatient ICD-9 Dx | 295 | schizophrenic disorders |  |
| Outpatient ICD-9 Dx | 296 | affective psychoses |  |
| Outpatient ICD-9 Dx | 298 | oth nonorganic psychoses |  |
| Outpatient ICD-9 Dx | 305 | nondependent drug abuse |  |
| Outpatient ICD-9 Dx | 311 | depressive disorder nec |  |
| Outpatient ICD-9 Dx | 331 | cerebral degeneration |  |
| Outpatient ICD-9 Dx | 342 | hemiplegia |  |
| Outpatient ICD-9 Dx | 345 | epilepsy |  |
| Outpatient ICD-9 Dx | 346 | migraine |  |
| Outpatient ICD-9 Dx | 348 | other brain conditions |  |
| Outpatient ICD-9 Dx | 354 | mononeuritis upper limb |  |
| Outpatient ICD-9 Dx | 355 | mononeuritis leg |  |
| Outpatient ICD-9 Dx | 362 | retinal disorders nec |  |
| Outpatient ICD-9 Dx | 365 | glaucoma |  |
| Outpatient ICD-9 Dx | 368 | visual disturbances |  |
| Outpatient ICD-9 Dx | 370 | keratitis |  |
| Outpatient ICD-9 Dx | 372 | disorders of conjunctiva |  |
| Outpatient ICD-9 Dx | 374 | disorders of eyelids nec |  |
| Outpatient ICD-9 Dx | 379 | eye disorders nec |  |
| Outpatient ICD-9 Dx | 380 | disorder of external ear |  |
| Outpatient ICD-9 Dx | 382 | otitis media |  |
| Outpatient ICD-9 Dx | 386 | vertiginous syndromes |  |
| Outpatient ICD-9 Dx | 389 | hearing loss |  |
| Outpatient ICD-9 Dx | 396 | mitral/aortic valve dis |  |
| Outpatient ICD-9 Dx | 401 | essential hypertension |  |
| Outpatient ICD-9 Dx | 402 | hypertensive heart dis |  |
| Outpatient ICD-9 Dx | 403 | hypertensive renal dis |  |
| Outpatient ICD-9 Dx | 410 | acute myocardial infarct |  |
| Outpatient ICD-9 Dx | 413 | angina pectoris |  |
| Outpatient ICD-9 Dx | 414 | oth chr ischemic hrt dis |  |
| Outpatient ICD-9 Dx | 424 | oth endocardial disease |  |
| Outpatient ICD-9 Dx | 425 | cardiomyopathy |  |
| Outpatient ICD-9 Dx | 426 | conduction disorders |  |
| Outpatient ICD-9 Dx | 427 | cardiac dysrhythmias |  |
| Outpatient ICD-9 Dx | 428 | heart failure |  |
| Outpatient ICD-9 Dx | 429 | ill-defined heart dis |  |
| Outpatient ICD-9 Dx | 433 | precerebral occlusion |  |
| Outpatient ICD-9 Dx | 434 | cerebral artery occlus |  |
| Outpatient ICD-9 Dx | 435 | transient cereb ischemia |  |
| Outpatient ICD-9 Dx | 436 | cva |  |
| Outpatient ICD-9 Dx | 437 | oth cerebrovasc disease |  |
| Outpatient ICD-9 Dx | 438 | late eff cerebrovasc dis |  |
| Outpatient ICD-9 Dx | 440 | atherosclerosis |  |
| Outpatient ICD-9 Dx | 443 | oth periph vascular dis |  |
| Outpatient ICD-9 Dx | 451 | thrombophlebitis |  |
| Outpatient ICD-9 Dx | 454 | varicose veins |  |
| Outpatient ICD-9 Dx | 455 | hemorrhoids |  |
| Outpatient ICD-9 Dx | 458 | hypotension |  |
| Outpatient ICD-9 Dx | 459 | oth circulatory disease |  |
| Outpatient ICD-9 Dx | 461 | acute sinusitis |  |
| Outpatient ICD-9 Dx | 477 | allergic rhinitis |  |
| Outpatient ICD-9 Dx | 486 | pneumonia |  |
| Outpatient ICD-9 Dx | 491 | chronic bronchitis |  |
| Outpatient ICD-9 Dx | 492 | emphysema |  |
| Outpatient ICD-9 Dx | 493 | asthma |  |
| Outpatient ICD-9 Dx | 496 | chr airway obstruct nec |  |
| Outpatient ICD-9 Dx | 511 | pleurisy |  |
| Outpatient ICD-9 Dx | 514 | pulm congest/hypostasis |  |
| Outpatient ICD-9 Dx | 518 | other lung diseases |  |
| Outpatient ICD-9 Dx | 530 | diseases of esophagus |  |
| Outpatient ICD-9 Dx | 533 | peptic ulcer |  |
| Outpatient ICD-9 Dx | 535 | gastritis and duodenitis |  |
| Outpatient ICD-9 Dx | 536 | stomach function disord |  |
| Outpatient ICD-9 Dx | 553 | other abdominal hernia |  |
| Outpatient ICD-9 Dx | 564 | funct digestive dis nec |  |
| Outpatient ICD-9 Dx | 573 | oth liver disorders |  |
| Outpatient ICD-9 Dx | 574 | cholelithiasis |  |
| Outpatient ICD-9 Dx | 578 | gastrointestinal hemorr |  |
| Outpatient ICD-9 Dx | 584 | acute renal failure |  |
| Outpatient ICD-9 Dx | 585 | chronic renal failure |  |
| Outpatient ICD-9 Dx | 586 | renal failure nos |  |
| Outpatient ICD-9 Dx | 588 | impaired renal function |  |
| Outpatient ICD-9 Dx | 593 | oth renal & ureteral dis |  |
| Outpatient ICD-9 Dx | 599 | oth urinary tract disor |  |
| Outpatient ICD-9 Dx | 600 | hyperplasia of prostate |  |
| Outpatient ICD-9 Dx | 611 | other breast disorders |  |
| Outpatient ICD-9 Dx | 627 | menopausal disorders |  |
| Outpatient ICD-9 Dx | 681 | cellulitis |  |
| Outpatient ICD-9 Dx | 682 | other cellulitis/abscess |  |
| Outpatient ICD-9 Dx | 686 | oth local skin infection |  |
| Outpatient ICD-9 Dx | 701 | oth skin hypertro/atroph |  |
| Outpatient ICD-9 Dx | 702 | other dermatoses |  |
| Outpatient ICD-9 Dx | 703 | diseases of nail |  |
| Outpatient ICD-9 Dx | 706 | sebaceous gland disease |  |
| Outpatient ICD-9 Dx | 707 | chronic ulcer of skin |  |
| Outpatient ICD-9 Dx | 709 | other skin disorders |  |
| Outpatient ICD-9 Dx | 714 | oth inflamm polyarthrop |  |
| Outpatient ICD-9 Dx | 715 | osteoarthrosis et al |  |
| Outpatient ICD-9 Dx | 716 | arthropathies nec/nos |  |
| Outpatient ICD-9 Dx | 719 | joint disorder nec & nos |  |
| Outpatient ICD-9 Dx | 721 | spondylosis et al |  |
| Outpatient ICD-9 Dx | 722 | intervertebral disc dis |  |
| Outpatient ICD-9 Dx | 723 | other cervical spine dis |  |
| Outpatient ICD-9 Dx | 724 | back disorder nec & nos |  |
| Outpatient ICD-9 Dx | 726 | periph enthesopathies |  |
| Outpatient ICD-9 Dx | 727 | oth dis synov/tend/bursa |  |
| Outpatient ICD-9 Dx | 728 | dis of muscle/lig/fascia |  |
| Outpatient ICD-9 Dx | 729 | other soft tissue dis |  |
| Outpatient ICD-9 Dx | 733 | oth bone & cartilage dis |  |
| Outpatient ICD-9 Dx | 735 | acq deformities of toe |  |
| Outpatient ICD-9 Dx | 739 | somatic dysfunction |  |
| Outpatient ICD-9 Dx | 746 | other congen heart anom |  |
| Outpatient ICD-9 Dx | 780 | general symptoms |  |
| Outpatient ICD-9 Dx | 781 | nerv/musculskel sys symp |  |
| Outpatient ICD-9 Dx | 782 | skin/oth integument symp |  |
| Outpatient ICD-9 Dx | 783 | nutrit/metab/devel symp |  |
| Outpatient ICD-9 Dx | 784 | symptoms invol head/neck |  |
| Outpatient ICD-9 Dx | 785 | cardiovascular sys symp |  |
| Outpatient ICD-9 Dx | 786 | resp sys/oth chest symp |  |
| Outpatient ICD-9 Dx | 787 | gi system symptoms |  |
| Outpatient ICD-9 Dx | 788 | urinary system symptoms |  |
| Outpatient ICD-9 Dx | 789 | oth abdomen/pelvis symp |  |
| Outpatient ICD-9 Dx | 790 | abnormal blood findings |  |
| Outpatient ICD-9 Dx | 793 | abn find-body struct nos |  |
| Outpatient ICD-9 Dx | 794 | abnormal function study |  |
| Outpatient ICD-9 Dx | 799 | oth ill-def morbid/mortl |  |
| Outpatient ICD-9 Dx | 840 | sprain shoulder & arm |  |
| Outpatient ICD-9 Dx | 847 | sprain of back nec/nos |  |
| Outpatient ICD-9 Dx | 924 | contusion leg & oth site |  |
| Outpatient ICD-9 Dx | 959 | injury nec/nos |  |
| Outpatient ICD-9 Dx | 995 | certain adverse eff nec |  |
| Outpatient ICD-9 Dx | 996 | replace & graft complic |  |
| Outpatient ICD-9 Dx | 998 | oth surgical compl nec |  |
| Outpatient ICD-9 Dx | E888 | fall nec & nos |  |
| Outpatient ICD-9 Dx | V03 | vaccin for bacterial dis |  |
| Outpatient ICD-9 Dx | V04 | vaccin for viral disease |  |
| Outpatient ICD-9 Dx | V12 | hx of disease nec |  |
| Outpatient ICD-9 Dx | V43 | organ replacement nec |  |
| Outpatient ICD-9 Dx | V45 | oth postsurgical states |  |
| Outpatient ICD-9 Dx | V58 | encountr proc/aftrcr nec |  |
| Outpatient ICD-9 Dx | V70 | general medical exam |  |
| Outpatient ICD-9 Dx | V71 | observation-suspect cond |  |
| Outpatient ICD-9 Dx | V72 | special examinations |  |
| Outpatient ICD-9 Dx | V76 | screening-malig neoplasm |  |
| Outpatient ICD-9 Px | 13 | operations on lens |  |
| Outpatient ICD-9 Px | 33 | other bronchial/lung ops | Yes |
| Outpatient ICD-9 Px | 37 | other heart/pericard ops |  |
| Outpatient ICD-9 Px | 38 | vessel inc/excis/occlus |  |
| Outpatient ICD-9 Px | 39 | other ops on vessels | Yes |
| Outpatient ICD-9 Px | 43 | gastric incision/excis | Yes |
| Outpatient ICD-9 Px | 45 | intest incis/excis/anast |  |
| Outpatient ICD-9 Px | 54 | other abdomen region ops | Yes |
| Outpatient ICD-9 Px | 57 | urinary bladder ops | Yes |
| Outpatient ICD-9 Px | 59 | other urinary tract ops | Yes |
| Outpatient ICD-9 Px | 83 | other mus/ten/fas/bur op | Yes |
| Outpatient ICD-9 Px | 86 | skin & subq operations |  |
| Outpatient ICD-9 Px | 87 | diagnostic radiology |  |
| Outpatient ICD-9 Px | 88 | other dx radiology |  |
| Outpatient ICD-9 Px | 89 | interview/consult/exam |  |
| Outpatient ICD-9 Px | 93 | pt |  |
| Outpatient ICD-9 Px | 95 | eye & ear dx/treatment | Yes |
| Outpatient ICD-9 Px | 96 | non-op intubat & irrigat | Yes |
| Outpatient ICD-9 Px | 97 | replace & remov devices | Yes |
| Outpatient ICD-9 Px | 99 | other nonoperative proc |  |
| Outpatient CPT-4 Px | 11720 | debride nail, 1-5 |  |
| Outpatient CPT-4 Px | 11721 | debride nail, 6 or more |  |
| Outpatient CPT-4 Px | 20610 | drain/inject, joint/bursa |  |
| Outpatient CPT-4 Px | 36415 | routine venipuncture |  |
| Outpatient CPT-4 Px | 43239 | upper gi endoscopy, biopsy |  |
| Outpatient CPT-4 Px | 70450 | ct head/brain w/o dye |  |
| Outpatient CPT-4 Px | 71010 | chest x-ray |  |
| Outpatient CPT-4 Px | 71020 | chest x-ray |  |
| Outpatient CPT-4 Px | 71260 | ct thorax w/dye |  |
| Outpatient CPT-4 Px | 72110 | x-ray lower spine |  |
| Outpatient CPT-4 Px | 72192 | ct pelvis w/o dye |  |
| Outpatient CPT-4 Px | 73510 | x-ray hip |  |
| Outpatient CPT-4 Px | 73560 | x-ray knee, 1 or 2 |  |
| Outpatient CPT-4 Px | 73630 | x-ray foot |  |
| Outpatient CPT-4 Px | 74000 | x-ray abdomen |  |
| Outpatient CPT-4 Px | 74150 | ct abdomen w/o dye |  |
| Outpatient CPT-4 Px | 74160 | ct abdomen w/dye |  |
| Outpatient CPT-4 Px | 76092 | 76092 |  |
| Outpatient CPT-4 Px | 76705 | echo exam abdomen |  |
| Outpatient CPT-4 Px | 76770 | us exam abdo back wall, comp |  |
| Outpatient CPT-4 Px | 76856 | us exam pelvic, complete |  |
| Outpatient CPT-4 Px | 78465 | heart image (3d), multiple |  |
| Outpatient CPT-4 Px | 78478 | heart wall motion add-on |  |
| Outpatient CPT-4 Px | 78480 | heart function add-on |  |
| Outpatient CPT-4 Px | 80048 | metabolic panel total ca |  |
| Outpatient CPT-4 Px | 80051 | electrolyte panel |  |
| Outpatient CPT-4 Px | 80053 | comprehen metabolic panel |  |
| Outpatient CPT-4 Px | 80061 | lipid panel |  |
| Outpatient CPT-4 Px | 80076 | hepatic function panel |  |
| Outpatient CPT-4 Px | 81000 | urinalysis, nonauto w/scope |  |
| Outpatient CPT-4 Px | 81001 | urinalysis, auto w/scope |  |
| Outpatient CPT-4 Px | 81002 | urinalysis nonauto w/o scope |  |
| Outpatient CPT-4 Px | 82040 | assay serum albumin |  |
| Outpatient CPT-4 Px | 82043 | microalbumin, quantitative |  |
| Outpatient CPT-4 Px | 82270 | occulture blood, feces |  |
| Outpatient CPT-4 Px | 82310 | assay calcium |  |
| Outpatient CPT-4 Px | 82465 | assay bld/serum cholesterol |  |
| Outpatient CPT-4 Px | 82565 | assay creatinine |  |
| Outpatient CPT-4 Px | 82570 | assay urine creatinine |  |
| Outpatient CPT-4 Px | 82607 | vitamin b-12 |  |
| Outpatient CPT-4 Px | 82728 | assay ferritin |  |
| Outpatient CPT-4 Px | 82746 | blood folic acid serum |  |
| Outpatient CPT-4 Px | 82947 | assay glucose, blood quant |  |
| Outpatient CPT-4 Px | 82962 | glucose blood test |  |
| Outpatient CPT-4 Px | 83540 | assay iron |  |
| Outpatient CPT-4 Px | 83550 | iron binding test |  |
| Outpatient CPT-4 Px | 83615 | lactate (ld) (ldh) enzyme |  |
| Outpatient CPT-4 Px | 83718 | assay lipoprotein |  |
| Outpatient CPT-4 Px | 83721 | assay blood lipoprotein |  |
| Outpatient CPT-4 Px | 83735 | assay magnesium |  |
| Outpatient CPT-4 Px | 83880 | natriuretic peptide |  |
| Outpatient CPT-4 Px | 84100 | assay phosphorus |  |
| Outpatient CPT-4 Px | 84132 | assay serum potassium |  |
| Outpatient CPT-4 Px | 84153 | assay psa, total |  |
| Outpatient CPT-4 Px | 84155 | assay protein, serum |  |
| Outpatient CPT-4 Px | 84436 | assay total thyroxine |  |
| Outpatient CPT-4 Px | 84439 | assay free thyroxine |  |
| Outpatient CPT-4 Px | 84443 | assay thyroid stim hormone |  |
| Outpatient CPT-4 Px | 84450 | transferase (ast) (sgot) |  |
| Outpatient CPT-4 Px | 84460 | alanine amino (alt) (sgpt) |  |
| Outpatient CPT-4 Px | 84478 | assay triglycerides |  |
| Outpatient CPT-4 Px | 84479 | assay thyroid (t3 or t4) |  |
| Outpatient CPT-4 Px | 84484 | assay troponin, quant |  |
| Outpatient CPT-4 Px | 84520 | assay urea nitrogen |  |
| Outpatient CPT-4 Px | 84550 | assay blood/uric acid |  |
| Outpatient CPT-4 Px | 85024 | 85024 |  |
| Outpatient CPT-4 Px | 85025 | complete cbc w/auto diff wbc |  |
| Outpatient CPT-4 Px | 85027 | complete cbc, automated |  |
| Outpatient CPT-4 Px | 85610 | prothrombin time |  |
| Outpatient CPT-4 Px | 85651 | rbc sed rate, nonautomated |  |
| Outpatient CPT-4 Px | 85730 | thromboplastin time, partial |  |
| Outpatient CPT-4 Px | 86038 | antinuclear antibodies |  |
| Outpatient CPT-4 Px | 86140 | c-reactive protein |  |
| Outpatient CPT-4 Px | 86430 | rheumatoid factor test |  |
| Outpatient CPT-4 Px | 86677 | helicobacter pylori |  |
| Outpatient CPT-4 Px | 86706 | hep b surface antibody |  |
| Outpatient CPT-4 Px | 86803 | hepatitis c ab test |  |
| Outpatient CPT-4 Px | 87040 | blood culture for bacteria |  |
| Outpatient CPT-4 Px | 87070 | culture, bacteria, other |  |
| Outpatient CPT-4 Px | 87077 | culture aerobic identify |  |
| Outpatient CPT-4 Px | 87086 | urine culture/colony count |  |
| Outpatient CPT-4 Px | 87088 | urine bacteria culture |  |
| Outpatient CPT-4 Px | 87186 | microbe susceptible, mic |  |
| Outpatient CPT-4 Px | 87340 | hepatitis b surface ag, eia |  |
| Outpatient CPT-4 Px | 88305 | tissue exam by pathologist |  |
| Outpatient CPT-4 Px | 90658 | flu vaccine, 3 yrs & >, im |  |
| Outpatient CPT-4 Px | 90659 | 90659 |  |
| Outpatient CPT-4 Px | 90801 | psych dx interview |  |
| Outpatient CPT-4 Px | 90862 | medication management |  |
| Outpatient CPT-4 Px | 92012 | eye exam established pat |  |
| Outpatient CPT-4 Px | 92020 | special eye evaluation |  |
| Outpatient CPT-4 Px | 92083 | visual field examination(s) |  |
| Outpatient CPT-4 Px | 92135 | ophth dx imaging post seg |  |
| Outpatient CPT-4 Px | 92225 | special eye exam initial |  |
| Outpatient CPT-4 Px | 92226 | special eye exam subsequent |  |
| Outpatient CPT-4 Px | 92340 | fitting of spectacles |  |
| Outpatient CPT-4 Px | 93000 | ecg complete |  |
| Outpatient CPT-4 Px | 93005 | ecg tracing |  |
| Outpatient CPT-4 Px | 93010 | ecg report |  |
| Outpatient CPT-4 Px | 93015 | cardiovascular stress test |  |
| Outpatient CPT-4 Px | 93017 | cardiovascular stress test |  |
| Outpatient CPT-4 Px | 93042 | rhythm ecg, report |  |
| Outpatient CPT-4 Px | 93306 | 93306 | Yes |
| Outpatient CPT-4 Px | 93307 | echo exam heart |  |
| Outpatient CPT-4 Px | 93320 | doppler echo exam heart |  |
| Outpatient CPT-4 Px | 93325 | doppler color flow add-on |  |
| Outpatient CPT-4 Px | 93556 | imaging, cardiac cath |  |
| Outpatient CPT-4 Px | 93965 | extremity study |  |
| Outpatient CPT-4 Px | 93970 | extremity study |  |
| Outpatient CPT-4 Px | 93971 | extremity study |  |
| Outpatient CPT-4 Px | 94760 | measure blood oxygen level |  |
| Outpatient CPT-4 Px | 95903 | motor nerve conduction test |  |
| Outpatient CPT-4 Px | 95904 | sense nerve conduction test |  |
| Outpatient CPT-4 Px | 97001 | pt evaluation |  |
| Outpatient CPT-4 Px | 97003 | ot evaluation |  |
| Outpatient CPT-4 Px | 97035 | ultrasound therapy |  |
| Outpatient CPT-4 Px | 97110 | therapeutic exercises |  |
| Outpatient CPT-4 Px | 97112 | neuromuscular reeducation |  |
| Outpatient CPT-4 Px | 97116 | gait training therapy |  |
| Outpatient CPT-4 Px | 97124 | massage therapy |  |
| Outpatient CPT-4 Px | 97140 | manual therapy |  |
| Outpatient CPT-4 Px | 97530 | therapeutic activities |  |
| Outpatient CPT-4 Px | 97535 | self care mngment training |  |
| Outpatient CPT-4 Px | 99203 | office/outpatient visit, new |  |
| Outpatient CPT-4 Px | 99204 | office/outpatient visit, new |  |
| Outpatient CPT-4 Px | 99211 | office/outpatient visit, est |  |
| Outpatient CPT-4 Px | 99212 | office/outpatient visit, est |  |
| Outpatient CPT-4 Px | 99213 | office/outpatient visit, est |  |
| Outpatient CPT-4 Px | 99214 | office/outpatient visit, est |  |
| Outpatient CPT-4 Px | 99222 | initial hospital care |  |
| Outpatient CPT-4 Px | 99223 | initial hospital care |  |
| Outpatient CPT-4 Px | 99231 | subsequent hospital care |  |
| Outpatient CPT-4 Px | 99232 | subsequent hospital care |  |
| Outpatient CPT-4 Px | 99233 | subsequent hospital care |  |
| Outpatient CPT-4 Px | 99238 | hospital discharge day |  |
| Outpatient CPT-4 Px | 99239 | hospital discharge day |  |
| Outpatient CPT-4 Px | 99252 | inpatient consultation |  |
| Outpatient CPT-4 Px | 99253 | inpatient consultation |  |
| Outpatient CPT-4 Px | 99254 | inpatient consultation |  |
| Outpatient CPT-4 Px | 99255 | inpatient consultation |  |
| Outpatient CPT-4 Px | 99282 | emergency dept visit |  |
| Outpatient CPT-4 Px | 99283 | emergency dept visit |  |
| Outpatient CPT-4 Px | 99284 | emergency dept visit |  |
| Outpatient CPT-4 Px | 99285 | emergency dept visit |  |
| Outpatient CPT-4 Px | 99291 | critical care, first hour |  |
| Outpatient CPT-4 Px | 99312 | 99312 |  |
| Outpatient CPT-4 Px | A0422 | A0422 |  |
| Outpatient CPT-4 Px | A0425 | A0425 |  |
| Outpatient CPT-4 Px | A0427 | A0427 |  |
| Outpatient CPT-4 Px | A0428 | A0428 |  |
| Outpatient CPT-4 Px | A0429 | A0429 |  |
| Outpatient CPT-4 Px | A9500 | A9500 |  |
| Outpatient CPT-4 Px | A9502 | A9502 |  |
| Outpatient CPT-4 Px | A9505 | A9505 |  |
| Outpatient CPT-4 Px | D0120 | D0120 |  |
| Outpatient CPT-4 Px | D0220 | D0220 |  |
| Outpatient CPT-4 Px | D0230 | D0230 |  |
| Outpatient CPT-4 Px | D1110 | D1110 |  |
| Outpatient CPT-4 Px | G0001 | G0001 |  |
| Outpatient CPT-4 Px | G0008 | G0008 |  |
| Outpatient CPT-4 Px | G0283 | G0283 |  |
| Outpatient CPT-4 Px | Q0092 | Q0092 |  |
| Outpatient CPT-4 Px | R0070 | R0070 |  |
| Outpatient CPT-4 Px | V2020 | V2020 |  |
| Outpatient CPT-4 Px | V2203 | V2203 |  |
| Outpatient CPT-4 Px | Z7502 | Z7502 |  |
| Outpatient CPT-4 Px | Z9525 | Z9525 |  |
| Other Setting ICD-9 Dx | 250 | diabetes mellitus |  |
| Other Setting ICD-9 Dx | 276 | fluid/electrolyte dis |  |
| Other Setting ICD-9 Dx | 280 | iron deficiency anemias | Yes |
| Other Setting ICD-9 Dx | 285 | anemia nec/nos |  |
| Other Setting ICD-9 Dx | 290 | senile/presenile psychos | Yes |
| Other Setting ICD-9 Dx | 294 | other organic psych cond |  |
| Other Setting ICD-9 Dx | 298 | oth nonorganic psychoses | Yes |
| Other Setting ICD-9 Dx | 311 | depressive disorder nec |  |
| Other Setting ICD-9 Dx | 331 | cerebral degeneration | Yes |
| Other Setting ICD-9 Dx | 362 | retinal disorders nec | Yes |
| Other Setting ICD-9 Dx | 401 | essential hypertension |  |
| Other Setting ICD-9 Dx | 410 | acute myocardial infarct | Yes |
| Other Setting ICD-9 Dx | 414 | oth chr ischemic hrt dis |  |
| Other Setting ICD-9 Dx | 424 | oth endocardial disease | Yes |
| Other Setting ICD-9 Dx | 427 | cardiac dysrhythmias | Yes |
| Other Setting ICD-9 Dx | 428 | heart failure |  |
| Other Setting ICD-9 Dx | 429 | ill-defined heart dis | Yes |
| Other Setting ICD-9 Dx | 436 | cva |  |
| Other Setting ICD-9 Dx | 438 | late eff cerebrovasc dis |  |
| Other Setting ICD-9 Dx | 486 | pneumonia | Yes |
| Other Setting ICD-9 Dx | 493 | asthma | Yes |
| Other Setting ICD-9 Dx | 496 | chr airway obstruct nec |  |
| Other Setting ICD-9 Dx | 530 | diseases of esophagus |  |
| Other Setting ICD-9 Dx | 562 | diverticula of intestine | Yes |
| Other Setting ICD-9 Dx | 585 | chronic renal failure | Yes |
| Other Setting ICD-9 Dx | 593 | oth renal & ureteral dis | Yes |
| Other Setting ICD-9 Dx | 599 | oth urinary tract disor |  |
| Other Setting ICD-9 Dx | 707 | chronic ulcer of skin | Yes |
| Other Setting ICD-9 Dx | 715 | osteoarthrosis et al |  |
| Other Setting ICD-9 Dx | 719 | joint disorder nec & nos |  |
| Other Setting ICD-9 Dx | 728 | dis of muscle/lig/fascia |  |
| Other Setting ICD-9 Dx | 733 | oth bone & cartilage dis |  |
| Other Setting ICD-9 Dx | 780 | general symptoms |  |
| Other Setting ICD-9 Dx | 781 | nerv/musculskel sys symp |  |
| Other Setting ICD-9 Dx | 787 | gi system symptoms |  |
| Other Setting ICD-9 Dx | 799 | oth ill-def morbid/mortl |  |
| Other Setting ICD-9 Dx | 820 | fracture neck of femur | Yes |
| Other Setting ICD-9 Dx | V57 | rehabilitation procedure |  |
| Other Setting ICD-9 Dx | V66 | convalescence | Yes |
| Other Setting ICD-9 Px | 93 | pt | Yes |

NDC: National Drug Code. ICD-9: International Classification of Diseases 9th Revision. CPT-4: Current Procedural Terminology. Dx: diagnosis. Px = procedure.
* Infrequently-occurring covariates excluded from the propensity score due to concerns of potential model instability.
