# Supplementary material for: Nonsteroidal anti-inflammatory drug choice and adverse outcomes in clopidogrel users: A retrospective cohort study
Source: PLoS One. 2018 Mar 14;13(3):e0193800. doi: 10.1371/journal.pone.0193800 (PMC5851628; doi:10.1371/journal.pone.0193800)
Supplement: S4 Table — (DOCX) [file pone.0193800.s009.docx]

**S4 Table. Characteristics of clopidogrel users by NSAID exposure group**

|  |  | **ibuprofen (N=69,779)** | **celecoxib (N=66,317)** | | | **diclofenac (N=18,593)** | | | **etodolac (N=2,807)** | | | **indomethacin (N=7,651)** | | | **meloxicam (N=25,459)** | | |
| --- | --- | --- | --- | --- | --- | --- | --- | --- | --- | --- | --- | --- | --- | --- | --- | --- | --- |
| **Characteristic** | **Group** | **%** | **%** | **S.Diff*** | **WCSD**^†^ | **%** | **S.Diff** | **WCSD** | **%** | **S.Diff** | **WCSD** | **%** | **S.Diff** | **WCSD** | **%** | **S.Diff** | **WCSD** |
| **ibuprofen vs. celecoxib, diclofenac, etodolac, indomethacin, and meloxicam** | | | | | | | | | | | | | | | | | |
| ***Demographic factors*** | | | | | | | | | | | | | | | | | |
| Age at cohort entry (continuous; years) | Median  (Q3-Q1) | Age=66.7  (56.1-75.4) | 73.4  (65.9-80.3) | 0.54 | 0.25 | 71.0  (62.2-78.2) | 0.31 | 0.17 | 66.2  (55.6-74.7) | 0.03 | 0.10 | 70.1  (60.1-77.9) | 0.23 | 0.19 | 72.3  (64.2-79.4) | 0.43 | 0.25 |
| Age group at cohort entry | 18 to <35 | 1.1 | 0.2 | 0.11 | 0.03 | 0.4 | 0.09 | 0.02 | 0.9 | 0.02 | 0.03 | 0.6 | 0.06 | 0.02 | 0.2 | 0.11 | 0.02 |
|  | 35 to <50 | 12.3 | 4.0 | 0.31 | 0.07 | 6.6 | 0.19 | 0.05 | 12.8 | 0.02 | 0.05 | 8.1 | 0.14 | 0.07 | 4.7 | 0.27 | 0.04 |
|  | 50 to <65 | 32.0 | 18.8 | 0.31 | 0.17 | 23.5 | 0.19 | 0.12 | 32.6 | 0.01 | 0.08 | 26.7 | 0.12 | 0.13 | 21.5 | 0.24 | 0.09 |
|  | 65 to <80 | 40.2 | 50.9 | 0.22 | 0.14 | 49.6 | 0.19 | 0.14 | 39.5 | 0.01 | 0.07 | 45.4 | 0.11 | 0.09 | 50.2 | 0.20 | 0.13 |
|  | 80 to ≤100 | 14.4 | 26.0 | 0.29 | 0.13 | 19.9 | 0.15 | 0.06 | 14.2 | 0.01 | 0.11 | 19.3 | 0.13 | 0.11 | 23.4 | 0.23 | 0.13 |
| Sex | female | 56.3 | 67.2 | 0.23 | 0.08 | 64.3 | 0.16 | 0.06 | 63.8 | 0.15 | 0.11 | 45.5 | 0.22 | 0.24 | 65.9 | 0.20 | 0.07 |
| Race | White | 40.4 | 42.6 | 0.05 | 0.10 | 42.2 | 0.04 | 0.09 | 57.5 | 0.35 | 0.10 | 46.4 | 0.12 | 0.14 | 41.7 | 0.03 | 0.12 |
|  | Black | 16.6 | 10.9 | 0.17 | 0.09 | 10.5 | 0.18 | 0.12 | 14.5 | 0.06 | 0.08 | 17.9 | 0.03 | 0.07 | 10.0 | 0.20 | 0.08 |
|  | Hispanic/  Latino | 21.2 | 14.6 | 0.17 | 0.15 | 25.8 | 0.11 | 0.29 | 11.6 | 0.26 | 0.14 | 10.1 | 0.31 | 0.25 | 15.0 | 0.16 | 0.14 |
|  | Other/  unknown | 21.7 | 31.8 | 0.23 | 0.13 | 21.6 | 0.00 | 0.17 | 16.3 | 0.14 | 0.12 | 25.6 | 0.09 | 0.14 | 33.3 | 0.26 | 0.11 |
| State of residence | CA | 37.9 | 42.7 | 0.10 | 0.17 | 32.7 | 0.11 | 0.08 | 15.2 | 0.53 | 0.39 | 35.5 | 0.05 | 0.08 | 38.6 | 0.01 | 0.13 |
|  | FL | 17.6 | 15.5 | 0.06 | 0.13 | 26.4 | 0.21 | 0.29 | 22.3 | 0.12 | 0.20 | 15.0 | 0.07 | 0.15 | 18.0 | 0.01 | 0.18 |
|  | NY | 28.3 | 27.5 | 0.02 | 0.13 | 27.4 | 0.02 | 0.21 | 21.1 | 0.17 | 0.32 | 27.9 | 0.01 | 0.14 | 33.0 | 0.10 | 0.17 |
|  | OH | 8.9 | 8.4 | 0.02 | 0.07 | 7.2 | 0.06 | 0.10 | 26.4 | 0.47 | 0.27 | 11.3 | 0.08 | 0.05 | 5.3 | 0.14 | 0.12 |
|  | PA | 7.2 | 5.9 | 0.05 | 0.05 | 6.2 | 0.04 | 0.04 | 15.0 | 0.25 | 0.26 | 10.2 | 0.11 | 0.08 | 5.1 | 0.09 | 0.06 |
| Calendar year of cohort entry | 2000 | 2.5 | 8.2 | 0.26 | 0.15 | 3.7 | 0.07 | 0.04 | 3.3 | 0.05 | 0.12 | 2.8 | 0.02 | 0.02 | 0.3 | 0.18 | 0.12 |
|  | 2001 | 3.6 | 11.6 | 0.30 | 0.16 | 4.7 | 0.05 | 0.03 | 4.1 | 0.02 | 0.07 | 4.8 | 0.06 | 0.04 | 2.2 | 0.09 | 0.07 |
|  | 2002 | 5.0 | 14.6 | 0.33 | 0.19 | 5.3 | 0.02 | 0.02 | 4.0 | 0.04 | 0.05 | 6.1 | 0.05 | 0.03 | 2.7 | 0.11 | 0.05 |
|  | 2003 | 6.6 | 14.7 | 0.27 | 0.17 | 5.5 | 0.04 | 0.07 | 5.3 | 0.05 | 0.08 | 7.6 | 0.04 | 0.03 | 3.6 | 0.13 | 0.07 |
|  | 2004 | 6.4 | 12.7 | 0.22 | 0.18 | 4.5 | 0.09 | 0.09 | 5.3 | 0.04 | 0.07 | 7.3 | 0.04 | 0.04 | 4.3 | 0.09 | 0.06 |
|  | 2005 | 10.8 | 6.4 | 0.16 | 0.15 | 12.1 | 0.04 | 0.04 | 12.1 | 0.04 | 0.06 | 10.8 | 0.00 | 0.06 | 15.2 | 0.13 | 0.19 |
|  | 2006 | 13.8 | 8.7 | 0.16 | 0.12 | 15.6 | 0.05 | 0.05 | 19.8 | 0.16 | 0.12 | 14.0 | 0.00 | 0.05 | 10.3 | 0.11 | 0.13 |
|  | 2007 | 13.0 | 6.8 | 0.21 | 0.10 | 13.1 | 0.00 | 0.04 | 14.3 | 0.04 | 0.07 | 11.9 | 0.03 | 0.06 | 13.9 | 0.03 | 0.06 |
|  | 2008 | 10.7 | 5.3 | 0.20 | 0.08 | 9.2 | 0.05 | 0.05 | 9.8 | 0.03 | 0.06 | 9.6 | 0.04 | 0.05 | 12.8 | 0.06 | 0.11 |
|  | 2009 | 13.1 | 5.3 | 0.27 | 0.11 | 13.0 | 0.00 | 0.03 | 10.7 | 0.07 | 0.08 | 12.6 | 0.01 | 0.04 | 16.7 | 0.10 | 0.14 |
|  | 2010 | 14.6 | 5.6 | 0.30 | 0.12 | 13.4 | 0.03 | 0.05 | 11.3 | 0.10 | 0.14 | 12.5 | 0.06 | 0.05 | 18.1 | 0.09 | 0.21 |
| Medicaid-Medicare dual-eligible | Yes | 68.3 | 82.7 | 0.34 | 0.20 | 77.8 | 0.22 | 0.10 | 74.7 | 0.14 | 0.15 | 77.6 | 0.21 | 0.16 | 80.3 | 0.28 | 0.08 |
| ***Healthcare utilization factors during the one-year baseline time*** | | | | | | | | | | | | | | | | | |
| Nursing home residence | Yes | 10.2 | 13.8 | 0.11 | 0.14 | 6.1 | 0.15 | 0.17 | 8.7 | 0.05 | 0.13 | 10.3 | 0.01 | 0.08 | 8.0 | 0.07 | 0.15 |
| Number of circulatory system hospitalizations | Median (Q3-Q1) | 0.0  (0.0-1.0) | 0.0 (0.0-1.0) | 0.02 | 0.05 | 0.0 (0.0-1.0) | 0.12 | 0.10 | 0.0 (0.0-1.0) | 0.11 | 0.14 | 0.0 (0.0-1.0) | 0.14 | 0.17 | 0.0 (0.0-1.0) | 0.14 | 0.12 |
| Number of non-circulatory system hospitalizations | Median (Q3-Q1) | 0.0  (0.0-1.0) | 0.0 (0.0-1.0) | 0.06 | 0.05 | 0.0 (0.0-0.0) | 0.14 | 0.10 | 0.0 (0.0-1.0) | 0.05 | 0.11 | 0.0 (0.0-1.0) | 0.00 | 0.08 | 0.0 (0.0-0.0) | 0.12 | 0.09 |
| Number of circulatory system ED^‡^ visits | Median (Q3-Q1) | 0.0  (0.0-1.0) | 0.0 (0.0-0.0) | 0.02 | 0.02 | 0.0 (0.0-0.0) | 0.02 | 0.03 | 0.0 (0.0-0.0) | 0.04 | 0.03 | 0.0 (0.0-1.0) | 0.04 | 0.04 | 0.0 (0.0-0.0) | 0.02 | 0.02 |
| Number of non-circulatory system ED visits | Median (Q3-Q1) | 1.0  (0.0-2.0) | 0.0 (0.0-2.0) | 0.04 | 0.03 | 0.0 (0.0-1.0) | 0.09 | 0.05 | 1.0 (0.0-2.0) | 0.04 | 0.06 | 1.0 (0.0-2.0) | 0.02 | 0.04 | 0.0 (0.0-1.0) | 0.11 | 0.07 |
| Number of non-circulatory system outpatient visits | Median (Q3-Q1) | 6.0  (2.0-11.0) | 7.0 (3.0-13.0) | 0.07 | 0.05 | 6.0 (2.0-11.0) | 0.04 | 0.03 | 5.0 (1.0-10.0) | 0.06 | 0.09 | 7.0 (3.0-14.0) | 0.11 | 0.08 | 6.0 (2.0-12.0) | 0.08 | 0.06 |
| Number of non-circulatory system outpatient visits | Median (Q3-Q1) | 21.0  (10.0-41.0) | 24.0 (13.0-44.0) | 0.04 | 0.06 | 22.0 (10.0-43.0) | 0.06 | 0.10 | 19.0 (8.0-37.0) | 0.03 | 0.08 | 20.0 (9.0-41.0) | 0.01 | 0.11 | 22.0 (11.0-44.0) | 0.08 | 0.08 |
| Number of prescription dispensings | Median (Q3-Q1) | 70.0  (41.0-109) | 71.0 (44.0-108) | 0.03 | 0.08 | 73.0 (44.0-111) | 0.05 | 0.08 | 74.0 (38.0-117) | 0.08 | 0.10 | 71.0 (42.0-111) | 0.03 | 0.08 | 74.0 (46.0-112) | 0.07 | 0.09 |
| Number of unique prescriptions | Median (Q3-Q1) | 17.0  (11.0-24.0) | 18.0 (13.0-25.0) | 0.13 | 0.10 | 18.0 (12.0-25.0) | 0.10 | 0.06 | 17.0 (11.0-24.0) | 0.02 | 0.10 | 17.0 (12.0-24.0) | 0.03 | 0.06 | 18.0 (13.0-25.0) | 0.12 | 0.08 |
| Number of inpatient diagnosis codes | Median (Q3-Q1) | 0.0  (0.0-9.0) | 0.0 (0.0-9.0) | 0.04 | 0.06 | 0.0 (0.0-8.0) | 0.14 | 0.11 | 0.0 (0.0-9.0) | 0.04 | 0.11 | 3.0 (0.0-9.0) | 0.11 | 0.14 | 0.0 (0.0-8.0) | 0.12 | 0.10 |
| Number of unique inpatient diagnosis codes | Median (Q3-Q1) | 0.0  (0.0-8.0) | 0.0 (0.0-8.0) | 0.01 | 0.07 | 0.0 (0.0-7.0) | 0.14 | 0.11 | 0.0 (0.0-8.0) | 0.00 | 0.10 | 3.0 (0.0-9.0) | 0.14 | 0.14 | 0.0 (0.0-7.0) | 0.12 | 0.10 |
| Number of inpatient ICD-9^§^ procedure codes | Median (Q3-Q1) | 0.0  (0.0-2.0) | 0.0 (0.0-2.0) | 0.03 | 0.04 | 0.0 (0.0-1.0) | 0.10 | 0.08 | 0.0 (0.0-2.0) | 0.02 | 0.07 | 0.0 (0.0-3.0) | 0.12 | 0.12 | 0.0 (0.0-1.0) | 0.09 | 0.09 |
| Number of unique inpatient ICD-9 procedure codes | Median (Q3-Q1) | 0.0  (0.0-1.0) | 0.0 (0.0-1.0) | 0.03 | 0.05 | 0.0 (0.0-1.0) | 0.09 | 0.08 | 0.0 (0.0-2.0) | 0.00 | 0.07 | 0.0 (0.0-2.0) | 0.13 | 0.12 | 0.0 (0.0-1.0) | 0.09 | 0.08 |
| Number of inpatient CPT-4^‖^/HCPCS^#^ procedure codes | Median (Q3-Q1) | 0.0  (0.0-0.0) | 0.0 (0.0-0.0) | 0.05 | 0.07 | 0.0 (0.0-0.0) | 0.01 | 0.02 | 0.0 (0.0-0.0) | 0.05 | 0.08 | 0.0 (0.0-0.0) | 0.01 | 0.03 | 0.0 (0.0-0.0) | 0.05 | 0.05 |
| Number of unique inpatient CPT-4/HCPCS procedure codes | Median (Q3-Q1) | 0.0  (0.0-0.0) | 0.0 (0.0-0.0) | 0.06 | 0.07 | 0.0 (0.0-0.0) | 0.01 | 0.02 | 0.0 (0.0-0.0) | 0.05 | 0.08 | 0.0 (0.0-0.0) | 0.01 | 0.03 | 0.0 (0.0-0.0) | 0.05 | 0.05 |
| Number of outpatient diagnosis codes | Median (Q3-Q1) | 63.0  (29.0-127) | 79.0 (43.0-146) | 0.11 | 0.08 | 68.0 (32.0-138) | 0.06 | 0.08 | 59.0 (24.0-113) | 0.03 | 0.10 | 69.0 (32.0-139) | 0.09 | 0.12 | 72.0 (34.0-148) | 0.11 | 0.10 |
| Number of unique outpatient diagnosis codes | Median (Q3-Q1) | 22.0  (12.0-33.0) | 26.0 (17.0-37.0) | 0.25 | 0.13 | 24.0 (14.0-35.0) | 0.10 | 0.08 | 21.0 (11.0-32.0) | 0.08 | 0.17 | 23.0 (13.0-36.0) | 0.09 | 0.10 | 24.0 (14.0-36.0) | 0.13 | 0.09 |
| Number of outpatient ICD-9 procedure codes | Median (Q3-Q1) | 0.0  (0.0-0.0) | 0.0 (0.0-0.0) | 0.19 | 0.05 | 0.0 (0.0-0.0) | 0.00 | 0.03 | 0.0 (0.0-0.0) | 0.02 | 0.04 | 0.0 (0.0-0.0) | 0.03 | 0.02 | 0.0 (0.0-0.0) | 0.04 | 0.06 |
| Number of unique outpatient ICD-9 procedure codes | Median (Q3-Q1) | 0.0  (0.0-0.0) | 0.0 (0.0-0.0) | 0.27 | 0.06 | 0.0 (0.0-0.0) | 0.00 | 0.04 | 0.0 (0.0-0.0) | 0.02 | 0.05 | 0.0 (0.0-0.0) | 0.08 | 0.07 | 0.0 (0.0-0.0) | 0.04 | 0.07 |
| Number of outpatient CPT-4/HCPCS procedure codes | Median (Q3-Q1) | 75.0  (35.0-145) | 90.0 (48.0-164) | 0.06 | 0.09 | 82.0 (39.0-158) | 0.02 | 0.06 | 66.0 (27.0-120) | 0.17 | 0.18 | 81.0 (37.0-152) | 0.00 | 0.09 | 84.0 (40.0-160) | 0.01 | 0.07 |
| Number of unique outpatient CPT-4/HCPCS procedure codes | Median (Q3-Q1) | 41.0  (22.0-63.0) | 45.0 (27.0-65.0) | 0.11 | 0.12 | 43.0 (23.0-66.0) | 0.05 | 0.07 | 38.0 (17.0-59.0) | 0.13 | 0.20 | 43.0 (22.0-66.0) | 0.04 | 0.09 | 42.0 (23.0-65.0) | 0.03 | 0.08 |
| Number of other diagnosis codes | Median (Q3-Q1) | 0.0  (0.0-0.0) | 0.0 (0.0-0.0) | 0.04 | 0.07 | 0.0 (0.0-0.0) | 0.03 | 0.10 | 0.0 (0.0-0.0) | 0.04 | 0.06 | 0.0 (0.0-0.0) | 0.01 | 0.08 | 0.0 (0.0-0.0) | 0.01 | 0.11 |
| Number of unique other diagnosis codes | Median (Q3-Q1) | 0.0  (0.0-0.0) | 0.0 (0.0-0.0) | 0.08 | 0.08 | 0.0 (0.0-0.0) | 0.08 | 0.11 | 0.0 (0.0-0.0) | 0.00 | 0.09 | 0.0 (0.0-0.0) | 0.03 | 0.05 | 0.0 (0.0-0.0) | 0.01 | 0.11 |
| Number of other ICD-9 procedure codes | Median (Q3-Q1) | 0.0  (0.0-0.0) | 0.0 (0.0-0.0) | 0.02 | 0.02 | 0.0 (0.0-0.0) | 0.02 | 0.03 | 0.0 (0.0-0.0) | 0.02 | 0.06 | 0.0 (0.0-0.0) | 0.02 | 0.03 | 0.0 (0.0-0.0) | 0.00 | 0.03 |
| Number of unique other ICD-9 procedure codes | Median (Q3-Q1) | 0.0  (0.0-0.0) | 0.0 (0.0-0.0) | 0.03 | 0.02 | 0.0 (0.0-0.0) | 0.01 | 0.02 | 0.0 (0.0-0.0) | 0.03 | 0.07 | 0.0 (0.0-0.0) | 0.03 | 0.03 | 0.0 (0.0-0.0) | 0.01 | 0.03 |
| ***Diseases during the one-year baseline time*** | | | | | | | | | | | | | | | | | |
| AMI*** | Yes | 8.8 | 7.5 | 0.05 | 0.02 | 6.9 | 0.07 | 0.04 | 7.8 | 0.03 | 0.06 | 10.8 | 0.07 | 0.08 | 6.1 | 0.10 | 0.05 |
| GIB*** | Yes | 1.5 | 2.1 | 0.05 | 0.03 | 1.3 | 0.02 | 0.02 | 1.8 | 0.03 | 0.05 | 2.4 | 0.07 | 0.05 | 1.3 | 0.01 | 0.03 |
| Ischemic stroke*** | Yes | 4.0 | 5.4 | 0.07 | 0.04 | 3.3 | 0.04 | 0.04 | 3.2 | 0.05 | 0.05 | 3.8 | 0.01 | 0.05 | 3.1 | 0.05 | 0.05 |
| Non-traumatic ICH*** | Yes | 0.3 | 0.3 | 0.01 | 0.02 | 0.2 | 0.01 | 0.02 | 🞵🞵 | 🞵🞵 | 🞵🞵 | 0.3 | 0.01 | 0.04 | 0.2 | 0.02 | 0.03 |
| Acute respiratory infection | Yes | 10.2 | 12.2 | 0.06 | 0.04 | 10.5 | 0.01 | 0.02 | 10.6 | 0.01 | 0.07 | 9.4 | 0.03 | 0.05 | 11.0 | 0.02 | 0.05 |
| Alcohol abuse | Yes | 3.7 | 2.5 | 0.07 | 0.05 | 2.0 | 0.10 | 0.08 | 3.8 | 0.00 | 0.08 | 3.6 | 0.01 | 0.03 | 2.0 | 0.10 | 0.06 |
| Angina pectoris | Yes | 22.2 | 25.7 | 0.08 | 0.02 | 22.8 | 0.01 | 0.03 | 19.0 | 0.08 | 0.12 | 24.9 | 0.06 | 0.07 | 23.3 | 0.02 | 0.04 |
| Artery disease | Yes | 34.7 | 39.1 | 0.09 | 0.10 | 38.9 | 0.09 | 0.08 | 31.4 | 0.07 | 0.09 | 36.6 | 0.04 | 0.07 | 37.7 | 0.06 | 0.09 |
| Asthma/COPD/emphysema | Yes | 32.1 | 33.7 | 0.03 | 0.05 | 32.9 | 0.02 | 0.04 | 36.7 | 0.10 | 0.11 | 32.4 | 0.01 | 0.06 | 31.5 | 0.01 | 0.05 |
| Atrial fibrillation | Yes | 9.2 | 12.2 | 0.10 | 0.07 | 9.1 | 0.00 | 0.03 | 7.8 | 0.05 | 0.06 | 13.2 | 0.13 | 0.12 | 10.1 | 0.03 | 0.06 |
| Cancer | Yes | 11.9 | 14.6 | 0.08 | 0.04 | 13.2 | 0.04 | 0.04 | 10.9 | 0.03 | 0.07 | 13.0 | 0.03 | 0.03 | 13.5 | 0.05 | 0.05 |
| Cardiac dysrhythmias | Yes | 22.8 | 27.2 | 0.10 | 0.04 | 23.2 | 0.01 | 0.04 | 19.6 | 0.08 | 0.09 | 27.7 | 0.11 | 0.11 | 23.8 | 0.02 | 0.05 |
| Cardiovascular system symptoms | Yes | 23.8 | 27.9 | 0.10 | 0.06 | 27.3 | 0.08 | 0.08 | 19.5 | 0.10 | 0.10 | 23.1 | 0.01 | 0.08 | 26.9 | 0.07 | 0.04 |
| Cerebrovascular disease: hemorrhage | Yes | 1.1 | 1.3 | 0.02 | 0.03 | 0.8 | 0.04 | 0.04 | 1.0 | 0.02 | 0.05 | 1.0 | 0.01 | 0.03 | 0.9 | 0.03 | 0.04 |
| Cerebrovascular disease: ischemic stroke | Yes | 18.7 | 23.1 | 0.11 | 0.08 | 15.7 | 0.08 | 0.08 | 15.7 | 0.08 | 0.08 | 18.1 | 0.01 | 0.06 | 15.9 | 0.07 | 0.09 |
| Cerebrovascular disease: other | Yes | 26.1 | 31.8 | 0.13 | 0.07 | 26.1 | 0.00 | 0.04 | 22.8 | 0.08 | 0.08 | 26.5 | 0.01 | 0.05 | 27.2 | 0.02 | 0.08 |
| Cerebrovascular disease: transient cerebral ischemia | Yes | 12.0 | 16.0 | 0.11 | 0.04 | 12.6 | 0.02 | 0.04 | 11.4 | 0.02 | 0.07 | 11.7 | 0.01 | 0.05 | 11.7 | 0.01 | 0.05 |
| Circulatory system disease: other | Yes | 89.5 | 93.7 | 0.15 | 0.06 | 89.1 | 0.01 | 0.06 | 85.4 | 0.12 | 0.11 | 91.0 | 0.05 | 0.05 | 90.8 | 0.05 | 0.04 |
| Conduction disorders | Yes | 6.0 | 7.0 | 0.04 | 0.04 | 5.7 | 0.01 | 0.03 | 4.5 | 0.07 | 0.09 | 8.0 | 0.08 | 0.07 | 6.2 | 0.01 | 0.04 |
| Congenital anomalies of heart | Yes | 4.4 | 5.4 | 0.05 | 0.04 | 4.1 | 0.02 | 0.03 | 2.7 | 0.09 | 0.06 | 4.9 | 0.02 | 0.04 | 4.8 | 0.02 | 0.04 |
| Diabetes mellitus | Yes | 52.3 | 51.8 | 0.01 | 0.07 | 51.1 | 0.02 | 0.03 | 47.3 | 0.10 | 0.08 | 53.4 | 0.02 | 0.05 | 52.6 | 0.01 | 0.05 |
| Fever | Yes | 2.6 | 2.5 | 0.00 | 0.03 | 1.7 | 0.06 | 0.05 | 1.6 | 0.07 | 0.08 | 3.2 | 0.04 | 0.05 | 2.0 | 0.04 | 0.04 |
| Gingival and periodontal disease | Yes | 0.3 | 0.2 | 0.03 | 0.02 | 0.1 | 0.04 | 0.04 | 🞵🞵 | 🞵🞵 | 🞵🞵 | 0.2 | 0.03 | 0.04 | 0.2 | 0.02 | 0.03 |
| Heart failure | Yes | 30.4 | 34.4 | 0.09 | 0.07 | 28.7 | 0.04 | 0.05 | 25.7 | 0.11 | 0.08 | 40.5 | 0.21 | 0.20 | 30.1 | 0.01 | 0.06 |
| HIV/AIDS | Yes | 2.0 | 0.7 | 0.11 | 0.05 | 0.8 | 0.11 | 0.05 | 0.9 | 0.10 | 0.08 | 1.3 | 0.05 | 0.03 | 0.6 | 0.13 | 0.04 |
| Hypertension | Yes | 81.0 | 86.9 | 0.16 | 0.07 | 83.0 | 0.05 | 0.06 | 77.0 | 0.10 | 0.10 | 84.4 | 0.09 | 0.06 | 85.1 | 0.11 | 0.03 |
| Hypothyroidism | Yes | 16.3 | 21.5 | 0.13 | 0.06 | 20.2 | 0.10 | 0.04 | 17.9 | 0.04 | 0.05 | 17.3 | 0.03 | 0.05 | 21.6 | 0.14 | 0.05 |
| Infection: other serious | Yes | 15.2 | 18.0 | 0.08 | 0.05 | 16.2 | 0.03 | 0.03 | 13.5 | 0.05 | 0.09 | 17.1 | 0.05 | 0.05 | 15.4 | 0.01 | 0.06 |
| Infectious and parasitic diseases: cytomegaloviral | Yes | 0.0 | 0.0 | 0.02 | 0.01 | 0.0 | 0.01 | 0.01 | 0.0 | 0.03 | 0.01 | 🞵🞵 | 🞵🞵 | 🞵🞵 | 0.0 | 0.02 | 0.01 |
| Infectious and parasitic diseases: Helicobacter pylori | Yes | 0.2 | 0.4 | 0.03 | 0.02 | 0.3 | 0.01 | 0.02 | 🞵🞵 | 🞵🞵 | 🞵🞵 | 0.3 | 0.01 | 0.04 | 0.3 | 0.02 | 0.02 |
| Infectious and parasitic diseases: herpes simplex | Yes | 0.2 | 0.3 | 0.02 | 0.02 | 0.3 | 0.02 | 0.03 | 🞵🞵 | 🞵🞵 | 🞵🞵 | 0.2 | 0.00 | 0.02 | 0.3 | 0.02 | 0.02 |
| Infectious and parasitic diseases: other | Yes | 19.9 | 22.4 | 0.06 | 0.08 | 19.8 | 0.00 | 0.07 | 17.9 | 0.05 | 0.08 | 20.4 | 0.01 | 0.05 | 19.4 | 0.01 | 0.09 |
| Ischemic heart disease | Yes | 60.4 | 60.7 | 0.01 | 0.07 | 58.2 | 0.05 | 0.07 | 56.4 | 0.08 | 0.10 | 67.6 | 0.15 | 0.14 | 60.2 | 0.01 | 0.04 |
| Lipoid metabolism disorder | Yes | 64.0 | 65.8 | 0.04 | 0.08 | 68.2 | 0.09 | 0.07 | 62.2 | 0.04 | 0.11 | 67.5 | 0.07 | 0.06 | 71.6 | 0.16 | 0.03 |
| Liver diseases | Yes | 12.4 | 13.6 | 0.04 | 0.05 | 12.7 | 0.01 | 0.04 | 10.1 | 0.07 | 0.07 | 12.7 | 0.01 | 0.05 | 12.8 | 0.01 | 0.03 |
| Mental disorder: depression | Yes | 24.1 | 24.0 | 0.00 | 0.07 | 24.8 | 0.02 | 0.06 | 24.1 | 0.00 | 0.08 | 18.6 | 0.13 | 0.08 | 21.5 | 0.06 | 0.08 |
| Mental disorder: other | Yes | 36.7 | 34.1 | 0.05 | 0.06 | 32.2 | 0.10 | 0.07 | 37.5 | 0.02 | 0.06 | 31.7 | 0.11 | 0.06 | 30.8 | 0.12 | 0.09 |
| Myocardial infarction: acute | Yes | 14.3 | 13.0 | 0.04 | 0.02 | 11.2 | 0.10 | 0.05 | 12.0 | 0.07 | 0.04 | 17.3 | 0.08 | 0.09 | 10.4 | 0.12 | 0.06 |
| Myocardial infarction: old | Yes | 11.3 | 9.1 | 0.07 | 0.06 | 8.7 | 0.09 | 0.04 | 11.3 | 0.00 | 0.06 | 13.8 | 0.07 | 0.09 | 8.4 | 0.10 | 0.04 |
| Nervous system disease: disorders of the eye and adnexa | Yes | 50.4 | 60.4 | 0.20 | 0.05 | 53.6 | 0.07 | 0.06 | 45.8 | 0.09 | 0.08 | 49.1 | 0.03 | 0.03 | 56.6 | 0.12 | 0.04 |
| Nervous system disease (central): hereditary and degenerative | Yes | 10.3 | 13.9 | 0.11 | 0.08 | 10.7 | 0.01 | 0.05 | 9.7 | 0.02 | 0.04 | 10.0 | 0.01 | 0.04 | 12.3 | 0.06 | 0.08 |
| Nervous system disease (central): inflammatory disease | Yes | 0.3 | 0.3 | 0.00 | 0.01 | 0.3 | 0.01 | 0.03 | 🞵🞵 | 🞵🞵 | 🞵🞵 | 0.3 | 0.00 | 0.03 | 0.3 | 0.01 | 0.02 |
| Nervous system disease (central): other | Yes | 13.9 | 15.0 | 0.03 | 0.04 | 12.0 | 0.05 | 0.04 | 13.9 | 0.00 | 0.06 | 12.1 | 0.05 | 0.05 | 11.6 | 0.07 | 0.06 |
| Nervous system disease (peripheral) | Yes | 16.5 | 19.5 | 0.08 | 0.05 | 19.8 | 0.09 | 0.05 | 17.0 | 0.01 | 0.10 | 16.7 | 0.01 | 0.07 | 18.6 | 0.06 | 0.03 |
| Obesity | Yes | 10.7 | 8.4 | 0.08 | 0.03 | 10.6 | 0.00 | 0.02 | 11.4 | 0.02 | 0.07 | 11.1 | 0.02 | 0.05 | 9.2 | 0.05 | 0.05 |
| Osteoarthritis | Yes | 31.4 | 52.1 | 0.43 | 0.13 | 48.0 | 0.35 | 0.15 | 35.1 | 0.08 | 0.14 | 34.0 | 0.06 | 0.12 | 47.3 | 0.33 | 0.08 |
| Pulmonary congestion and hypostasis | Yes | 4.6 | 4.8 | 0.01 | 0.04 | 3.2 | 0.07 | 0.07 | 3.9 | 0.03 | 0.06 | 6.2 | 0.07 | 0.08 | 3.7 | 0.05 | 0.05 |
| Pacemaker/ICD** | Yes | 1.1 | 1.2 | 0.00 | 0.03 | 1.0 | 0.02 | 0.03 | 1.0 | 0.01 | 0.05 | 1.8 | 0.06 | 0.08 | 1.1 | 0.01 | 0.03 |
| Pneumonia | Yes | 4.6 | 5.8 | 0.06 | 0.04 | 4.0 | 0.03 | 0.03 | 4.2 | 0.02 | 0.05 | 5.7 | 0.05 | 0.06 | 4.2 | 0.02 | 0.04 |
| Renal disease: chronic | Yes | 9.6 | 7.7 | 0.07 | 0.06 | 7.6 | 0.07 | 0.03 | 7.3 | 0.08 | 0.10 | 17.7 | 0.24 | 0.22 | 8.8 | 0.03 | 0.06 |
| Renal disease: other | Yes | 17.5 | 18.7 | 0.03 | 0.06 | 17.1 | 0.01 | 0.04 | 14.1 | 0.10 | 0.08 | 26.2 | 0.21 | 0.19 | 18.0 | 0.01 | 0.06 |
| Renal failure: acute | Yes | 5.3 | 4.5 | 0.03 | 0.04 | 4.2 | 0.05 | 0.03 | 4.1 | 0.06 | 0.09 | 9.6 | 0.16 | 0.17 | 4.6 | 0.03 | 0.05 |
| Rheumatoid arthritis | Yes | 5.0 | 8.7 | 0.14 | 0.06 | 9.1 | 0.16 | 0.10 | 5.0 | 0.00 | 0.10 | 5.2 | 0.01 | 0.08 | 7.9 | 0.11 | 0.07 |
| Stent placement | Yes | 12.4 | 11.7 | 0.02 | 0.04 | 11.6 | 0.02 | 0.04 | 13.0 | 0.02 | 0.07 | 15.3 | 0.08 | 0.08 | 11.7 | 0.02 | 0.05 |
| Substance abuse | Yes | 13.2 | 7.4 | 0.19 | 0.05 | 8.6 | 0.15 | 0.08 | 16.1 | 0.08 | 0.07 | 11.3 | 0.06 | 0.04 | 8.2 | 0.16 | 0.06 |
| Tobacco use | Yes | 16.4 | 10.1 | 0.19 | 0.04 | 11.7 | 0.13 | 0.07 | 19.6 | 0.08 | 0.07 | 15.4 | 0.03 | 0.03 | 11.3 | 0.15 | 0.05 |
| Urinary tract infection | Yes | 11.1 | 14.4 | 0.10 | 0.05 | 13.0 | 0.06 | 0.04 | 9.7 | 0.05 | 0.10 | 11.2 | 0.00 | 0.03 | 12.7 | 0.05 | 0.06 |
| ***Drugs during the one-year baseline time*** | | | | | | | | | | | | | | | | | |
| Agents for migraine | Yes | 1.7 | 1.8 | 0.00 | 0.03 | 2.0 | 0.02 | 0.02 | 2.1 | 0.03 | 0.04 | 1.3 | 0.03 | 0.04 | 1.8 | 0.01 | 0.03 |
| Antiadrenergic agents | Yes | 16.4 | 17.2 | 0.02 | 0.06 | 17.2 | 0.02 | 0.05 | 12.4 | 0.11 | 0.07 | 21.4 | 0.13 | 0.13 | 17.7 | 0.04 | 0.07 |
| Antialcohol agents | Yes | 0.1 | 0.0 | 0.02 | 0.01 | 0.0 | 0.01 | 0.01 | 🞵🞵 | 🞵🞵 | 🞵🞵 | 0.0 | 0.01 | 0.02 | 0.0 | 0.02 | 0.01 |
| Antiarrhythmic agents | Yes | 2.6 | 2.9 | 0.02 | 0.03 | 2.4 | 0.02 | 0.02 | 2.1 | 0.03 | 0.06 | 3.9 | 0.07 | 0.09 | 2.6 | 0.00 | 0.02 |
| Anticoagulants | Yes | 5.3 | 6.6 | 0.05 | 0.04 | 5.0 | 0.01 | 0.02 | 4.5 | 0.04 | 0.06 | 7.9 | 0.10 | 0.10 | 4.9 | 0.02 | 0.03 |
| Anticonvulsants | Yes | 8.8 | 6.9 | 0.07 | 0.03 | 7.0 | 0.07 | 0.07 | 9.7 | 0.03 | 0.07 | 6.8 | 0.07 | 0.04 | 6.6 | 0.08 | 0.05 |
| Antidepressants | Yes | 40.0 | 40.2 | 0.00 | 0.06 | 41.7 | 0.03 | 0.05 | 44.9 | 0.10 | 0.06 | 33.0 | 0.15 | 0.10 | 37.9 | 0.04 | 0.08 |
| Antidepressants: monoamine oxidase inhibitors | Yes | 0.0 | 0.0 | 0.00 | 0.01 | 0.0 | 0.00 | 0.01 | 0.0 | 0.02 | 0.04 | 0.0 | 0.01 | 0.01 | 0.0 | 0.00 | 0.01 |
| Antidepressants: other | Yes | 9.8 | 8.1 | 0.06 | 0.03 | 8.7 | 0.04 | 0.05 | 12.9 | 0.10 | 0.05 | 7.5 | 0.08 | 0.07 | 7.9 | 0.06 | 0.04 |
| Antidepressants: selective serotonin reuptake inhibitors | Yes | 27.0 | 26.9 | 0.00 | 0.07 | 28.7 | 0.04 | 0.05 | 29.8 | 0.06 | 0.07 | 20.7 | 0.15 | 0.10 | 26.0 | 0.02 | 0.06 |
| Antidepressants: serotonin and norepinephrine reuptake inhibitors | Yes | 5.4 | 4.6 | 0.04 | 0.02 | 6.5 | 0.05 | 0.02 | 7.1 | 0.07 | 0.06 | 4.8 | 0.03 | 0.02 | 6.1 | 0.03 | 0.03 |
| Antidepressants: tetracyclic | Yes | 3.3 | 3.5 | 0.01 | 0.04 | 3.2 | 0.01 | 0.03 | 3.5 | 0.01 | 0.07 | 3.0 | 0.02 | 0.03 | 2.9 | 0.02 | 0.04 |
| Antidepressants: tricyclic | Yes | 8.4 | 10.2 | 0.06 | 0.04 | 9.2 | 0.03 | 0.03 | 10.4 | 0.07 | 0.06 | 8.0 | 0.01 | 0.05 | 7.5 | 0.03 | 0.05 |
| Antidiabetic agents: insulin | Yes | 18.8 | 13.9 | 0.13 | 0.08 | 14.0 | 0.13 | 0.06 | 17.1 | 0.04 | 0.04 | 17.2 | 0.04 | 0.05 | 13.7 | 0.14 | 0.08 |
| Antidiabetic agents: non-insulin | Yes | 36.6 | 33.0 | 0.08 | 0.04 | 35.0 | 0.03 | 0.03 | 35.0 | 0.03 | 0.06 | 37.1 | 0.01 | 0.06 | 35.0 | 0.03 | 0.05 |
| Anti-infectives | Yes | 71.5 | 73.0 | 0.03 | 0.04 | 70.3 | 0.03 | 0.03 | 68.4 | 0.07 | 0.06 | 69.4 | 0.05 | 0.04 | 71.0 | 0.01 | 0.04 |
| Anti-infectives in 7 days prior to cohort entry | Yes | 7.0 | 4.8 | 0.09 | 0.04 | 4.3 | 0.12 | 0.04 | 6.3 | 0.03 | 0.10 | 6.4 | 0.02 | 0.05 | 4.1 | 0.13 | 0.06 |
| Antiobesity agents | Yes | 0.1 | 0.1 | 0.01 | 0.02 | 0.1 | 0.01 | 0.02 | 🞵🞵 | 🞵🞵 | 🞵🞵 | 0.0 | 0.02 | 0.02 | 0.1 | 0.01 | 0.02 |
| Antiplatelet agents excluding clopidogrel | Yes | 5.2 | 6.5 | 0.05 | 0.03 | 6.2 | 0.05 | 0.03 | 4.5 | 0.03 | 0.08 | 5.2 | 0.00 | 0.03 | 6.1 | 0.04 | 0.04 |
| Antipsychotics | Yes | 15.5 | 13.4 | 0.06 | 0.07 | 13.9 | 0.05 | 0.07 | 13.3 | 0.06 | 0.08 | 9.7 | 0.18 | 0.09 | 12.0 | 0.10 | 0.08 |
| Antiretroviral agents | Yes | 1.6 | 0.5 | 0.11 | 0.04 | 0.4 | 0.12 | 0.06 | 0.7 | 0.08 | 0.05 | 1.0 | 0.06 | 0.04 | 0.5 | 0.11 | 0.03 |
| Aspirin | Yes | 37.9 | 39.1 | 0.02 | 0.08 | 34.5 | 0.07 | 0.05 | 22.8 | 0.33 | 0.22 | 32.9 | 0.11 | 0.06 | 35.0 | 0.06 | 0.09 |
| Benzisoxazoles | Yes | 4.7 | 4.2 | 0.03 | 0.05 | 4.0 | 0.03 | 0.07 | 4.2 | 0.03 | 0.10 | 2.8 | 0.10 | 0.05 | 3.1 | 0.08 | 0.06 |
| Beta-adrenergic agents and alpha/beta-adrenergic blocking agents | Yes | 58.3 | 49.0 | 0.19 | 0.09 | 54.0 | 0.09 | 0.05 | 54.0 | 0.09 | 0.11 | 65.9 | 0.16 | 0.18 | 57.4 | 0.02 | 0.05 |
| Bronchodilators/inhaled corticosteroids | Yes | 30.7 | 28.6 | 0.05 | 0.03 | 29.7 | 0.02 | 0.04 | 34.6 | 0.08 | 0.07 | 30.3 | 0.01 | 0.04 | 29.4 | 0.03 | 0.05 |
| Calcium channel blockers | Yes | 38.9 | 45.0 | 0.12 | 0.02 | 41.1 | 0.04 | 0.03 | 35.6 | 0.07 | 0.09 | 45.9 | 0.14 | 0.12 | 42.7 | 0.08 | 0.03 |
| CYP1A2 inducers | Yes | 35.3 | 31.2 | 0.09 | 0.05 | 35.0 | 0.01 | 0.03 | 35.7 | 0.01 | 0.05 | 32.6 | 0.06 | 0.04 | 36.0 | 0.02 | 0.03 |
| CYP1A2 inhibitors | Yes | 11.5 | 11.7 | 0.01 | 0.03 | 11.4 | 0.00 | 0.02 | 12.5 | 0.03 | 0.05 | 18.4 | 0.19 | 0.12 | 9.9 | 0.05 | 0.03 |
| CYP2B6 inducers | Yes | 2.3 | 2.0 | 0.02 | 0.02 | 1.6 | 0.05 | 0.02 | 1.7 | 0.04 | 0.06 | 1.7 | 0.04 | 0.03 | 1.4 | 0.07 | 0.03 |
| CYP2B6 inhibitors | Yes | 0.1 | 0.2 | 0.03 | 0.02 | 0.1 | 0.01 | 0.03 | 🞵🞵 | 🞵🞵 | 🞵🞵 | 🞵🞵 | 🞵🞵 | 🞵🞵 | 0.1 | 0.00 | 0.02 |
| CYP2C19 inducers | Yes | 5.8 | 5.1 | 0.03 | 0.03 | 4.9 | 0.04 | 0.03 | 6.9 | 0.05 | 0.08 | 6.7 | 0.04 | 0.05 | 4.4 | 0.06 | 0.05 |
| CYP2C19 inhibitors | Yes | 28.9 | 30.8 | 0.04 | 0.05 | 31.6 | 0.06 | 0.03 | 31.8 | 0.06 | 0.06 | 42.1 | 0.28 | 0.14 | 31.8 | 0.06 | 0.04 |
| CYP2C9 inducers | Yes | 1.8 | 1.8 | 0.00 | 0.02 | 1.5 | 0.02 | 0.02 | 1.5 | 0.02 | 0.06 | 1.4 | 0.03 | 0.03 | 1.3 | 0.05 | 0.03 |
| CYP2C9 inhibitors | Yes | 15.8 | 13.9 | 0.05 | 0.02 | 14.6 | 0.03 | 0.03 | 17.1 | 0.04 | 0.05 | 14.4 | 0.04 | 0.03 | 13.3 | 0.07 | 0.04 |
| CYP3A4/5 inducers | Yes | 12.2 | 11.6 | 0.02 | 0.02 | 11.4 | 0.03 | 0.03 | 13.5 | 0.04 | 0.05 | 12.3 | 0.00 | 0.03 | 11.8 | 0.01 | 0.03 |
| CYP3A4/5 inhibitors | Yes | 42.7 | 42.0 | 0.01 | 0.04 | 41.5 | 0.02 | 0.03 | 42.9 | 0.00 | 0.04 | 43.0 | 0.01 | 0.06 | 40.2 | 0.05 | 0.04 |
| Dibenzazepines | Yes | 8.4 | 6.8 | 0.06 | 0.05 | 7.5 | 0.03 | 0.06 | 6.7 | 0.07 | 0.10 | 4.9 | 0.14 | 0.08 | 6.4 | 0.08 | 0.07 |
| Dipyridamole | Yes | 1.4 | 1.7 | 0.02 | 0.02 | 1.6 | 0.01 | 0.03 | 1.1 | 0.02 | 0.06 | 1.5 | 0.01 | 0.02 | 1.8 | 0.03 | 0.03 |
| Diuretics: loop | Yes | 25.7 | 27.0 | 0.03 | 0.07 | 25.1 | 0.01 | 0.04 | 28.4 | 0.06 | 0.07 | 38.6 | 0.28 | 0.25 | 24.4 | 0.03 | 0.07 |
| Diuretics: other | Yes | 33.0 | 33.6 | 0.01 | 0.04 | 37.5 | 0.09 | 0.03 | 32.8 | 0.00 | 0.06 | 38.8 | 0.12 | 0.08 | 38.3 | 0.11 | 0.02 |
| Estrogens | Yes | 5.2 | 8.8 | 0.14 | 0.05 | 5.8 | 0.03 | 0.04 | 8.1 | 0.12 | 0.08 | 4.1 | 0.05 | 0.06 | 5.5 | 0.01 | 0.03 |
| Fibrates | Yes | 7.8 | 6.2 | 0.07 | 0.04 | 7.7 | 0.01 | 0.01 | 8.1 | 0.01 | 0.04 | 8.0 | 0.00 | 0.05 | 7.7 | 0.01 | 0.03 |
| H2-receptor antagonists | Yes | 18.0 | 19.7 | 0.04 | 0.03 | 19.1 | 0.03 | 0.06 | 18.0 | 0.00 | 0.07 | 17.5 | 0.01 | 0.04 | 14.8 | 0.09 | 0.05 |
| Influenza vaccination | Yes | 23.0 | 32.4 | 0.21 | 0.11 | 24.4 | 0.03 | 0.10 | 26.0 | 0.07 | 0.10 | 27.0 | 0.09 | 0.07 | 32.0 | 0.20 | 0.05 |
| Inotropic agents | Yes | 6.2 | 8.8 | 0.10 | 0.03 | 6.1 | 0.01 | 0.02 | 5.5 | 0.03 | 0.07 | 9.7 | 0.13 | 0.12 | 5.7 | 0.02 | 0.02 |
| Leukotriene formation inhibitors and leukotriene receptor antagonists | Yes | 7.9 | 8.7 | 0.03 | 0.03 | 9.6 | 0.06 | 0.03 | 9.4 | 0.05 | 0.09 | 6.9 | 0.04 | 0.04 | 10.7 | 0.10 | 0.05 |
| Non-study NSAIDs | Yes | 1.4 | 2.0 | 0.04 | 0.03 | 2.6 | 0.09 | 0.06 | 2.0 | 0.04 | 0.02 | 1.6 | 0.01 | 0.04 | 1.8 | 0.03 | 0.03 |
| Oral contraceptives | Yes | 0.6 | 0.5 | 0.01 | 0.04 | 0.4 | 0.03 | 0.02 | 0.5 | 0.00 | 0.02 | 0.3 | 0.04 | 0.02 | 0.3 | 0.03 | 0.04 |
| Phenothiazines | Yes | 3.2 | 3.4 | 0.01 | 0.03 | 2.9 | 0.02 | 0.03 | 2.8 | 0.02 | 0.07 | 2.4 | 0.05 | 0.05 | 2.7 | 0.03 | 0.04 |
| Phenylbutylpiperidines | Yes | 1.1 | 0.9 | 0.02 | 0.02 | 0.7 | 0.04 | 0.02 | 0.9 | 0.02 | 0.04 | 0.5 | 0.06 | 0.03 | 0.6 | 0.05 | 0.02 |
| Potassium supplements | Yes | 15.8 | 17.2 | 0.04 | 0.05 | 15.6 | 0.01 | 0.04 | 20.0 | 0.11 | 0.07 | 21.9 | 0.16 | 0.13 | 15.0 | 0.02 | 0.06 |
| Proton pump inhibitors | Yes | 47.6 | 54.3 | 0.14 | 0.09 | 53.6 | 0.12 | 0.03 | 47.6 | 0.00 | 0.07 | 47.1 | 0.01 | 0.08 | 57.4 | 0.20 | 0.07 |
| Quinolinones | Yes | 2.1 | 0.9 | 0.10 | 0.03 | 2.0 | 0.01 | 0.02 | 1.4 | 0.06 | 0.06 | 0.8 | 0.10 | 0.04 | 1.5 | 0.04 | 0.03 |
| Ranolazine | Yes | 0.6 | 0.3 | 0.05 | 0.01 | 0.6 | 0.01 | 0.01 | 0.5 | 0.02 | 0.04 | 1.0 | 0.04 | 0.05 | 1.0 | 0.04 | 0.04 |
| Renin angiotensin system antagonists | Yes | 67.7 | 63.9 | 0.08 | 0.07 | 67.5 | 0.00 | 0.02 | 62.7 | 0.11 | 0.11 | 72.6 | 0.11 | 0.11 | 70.0 | 0.05 | 0.04 |
| Statins | Yes | 67.5 | 57.2 | 0.21 | 0.09 | 65.4 | 0.04 | 0.05 | 63.8 | 0.08 | 0.11 | 68.4 | 0.02 | 0.08 | 68.9 | 0.03 | 0.04 |
| Thiazides diuretics | Yes | 30.4 | 31.5 | 0.02 | 0.04 | 35.3 | 0.10 | 0.04 | 30.5 | 0.00 | 0.06 | 34.5 | 0.09 | 0.05 | 36.0 | 0.12 | 0.02 |
| Thiazolidinediones | Yes | 14.6 | 14.9 | 0.01 | 0.04 | 14.5 | 0.00 | 0.05 | 14.8 | 0.01 | 0.05 | 14.7 | 0.00 | 0.04 | 15.7 | 0.03 | 0.04 |
| Thioxanthines | Yes | 0.1 | 0.1 | 0.01 | 0.01 | 0.1 | 0.01 | 0.02 | 🞵🞵 | 🞵🞵 | 🞵🞵 | 🞵🞵 | 🞵🞵 | 🞵🞵 | 0.1 | 0.01 | 0.01 |
| Thyroid hormones | Yes | 10.8 | 11.8 | 0.03 | 0.05 | 12.0 | 0.04 | 0.02 | 14.9 | 0.12 | 0.06 | 11.9 | 0.03 | 0.05 | 12.4 | 0.05 | 0.04 |
| Varenicline | Yes | 1.2 | 0.4 | 0.09 | 0.03 | 0.9 | 0.04 | 0.03 | 1.4 | 0.01 | 0.06 | 1.0 | 0.03 | 0.02 | 1.0 | 0.02 | 0.03 |
| Vasodilators | Yes | 34.6 | 35.9 | 0.03 | 0.05 | 31.6 | 0.06 | 0.06 | 33.0 | 0.04 | 0.10 | 42.9 | 0.17 | 0.19 | 32.1 | 0.05 | 0.04 |
| Warfarin | Yes | 4.6 | 6.0 | 0.06 | 0.04 | 4.5 | 0.01 | 0.02 | 3.8 | 0.04 | 0.06 | 7.2 | 0.11 | 0.10 | 4.3 | 0.01 | 0.03 |

|  |  | **ibuprofen (N=69,779)** | **nabumetone**  **(N=7,060)** | | | **naproxen**  **(N=36,577)** | | | **rofecoxib**  **(N=26,247)** | | | **valdecoxib**  **(N=7,624)** | | |
| --- | --- | --- | --- | --- | --- | --- | --- | --- | --- | --- | --- | --- | --- | --- |
| **Characteristic** | **Group** | **%** | **%** | **S.Diff *** | **WCSD**^†^ | **%** | **S.Diff** | **WCSD** | **%** | **S.Diff** | **WCSD** | **%** | **S.Diff** | **WCSD** |
| **ibuprofen vs. nabumetone, naproxen, rofecoxib, and valdecoxib** | | | | | | | | | | | | | | |
| ***Demographic factors*** | | | | | | | | | | | | | | |
| Age at cohort entry  (continuous; years) | Median  (Q3-Q1) | Age=66.7  (56.1-75.4) | 69.3  (59.3-77.4) | 0.19 | 0.13 | 67.2 (56.9-75.9) | 0.05 | 0.03 | 72.7 (64.1-79.8) | 0.44 | 0.46 | 72.1 (64.1-79.2) | 0.41 | 0.52 |
| Age group at cohort entry | 18 to <35 | 1.1 | 0.5 | 0.07 | 0.03 | 0.9 | 0.02 | 0.01 | 0.4 | 0.09 | 0.07 | 0.3 | 0.10 | 0.08 |
|  | 35 to <50 | 12.3 | 8.9 | 0.11 | 0.05 | 10.9 | 0.04 | 0.01 | 5.3 | 0.25 | 0.22 | 5.0 | 0.26 | 0.22 |
|  | 50 to <65 | 32.0 | 27.5 | 0.10 | 0.07 | 31.8 | 0.01 | 0.02 | 21.2 | 0.25 | 0.44 | 21.9 | 0.23 | 0.46 |
|  | 65 to <80 | 40.2 | 44.9 | 0.10 | 0.09 | 41.2 | 0.02 | 0.03 | 48.6 | 0.17 | 0.20 | 50.4 | 0.21 | 0.22 |
|  | 80 to ≤100 | 14.4 | 18.2 | 0.10 | 0.06 | 15.2 | 0.02 | 0.02 | 24.5 | 0.26 | 0.20 | 22.4 | 0.21 | 0.25 |
| Sex | female | 56.3 | 67.5 | 0.23 | 0.08 | 60.6 | 0.09 | 0.05 | 69.0 | 0.26 | 0.12 | 70.8 | 0.31 | 0.13 |
| Race | White | 40.4 | 48.2 | 0.16 | 0.08 | 42.2 | 0.04 | 0.06 | 48.7 | 0.17 | 0.11 | 50.4 | 0.20 | 0.07 |
|  | Black | 16.6 | 12.6 | 0.11 | 0.07 | 16.3 | 0.01 | 0.04 | 11.8 | 0.14 | 0.13 | 10.8 | 0.17 | 0.12 |
|  | Hispanic/  Latino | 21.2 | 17.9 | 0.08 | 0.12 | 18.7 | 0.06 | 0.06 | 12.5 | 0.24 | 0.21 | 12.9 | 0.22 | 0.20 |
|  | Other/  unknown | 21.7 | 21.2 | 0.01 | 0.10 | 22.8 | 0.02 | 0.05 | 27.0 | 0.12 | 0.25 | 25.9 | 0.10 | 0.13 |
| State of residence | CA | 37.9 | 27.6 | 0.22 | 0.18 | 32.0 | 0.12 | 0.07 | 33.7 | 0.09 | 0.29 | 23.9 | 0.31 | 0.31 |
|  | FL | 17.6 | 21.8 | 0.11 | 0.16 | 18.6 | 0.03 | 0.08 | 16.4 | 0.03 | 0.16 | 20.6 | 0.07 | 0.23 |
|  | NY | 28.3 | 28.5 | 0.00 | 0.15 | 28.3 | 0.00 | 0.09 | 34.5 | 0.13 | 0.35 | 40.5 | 0.26 | 0.34 |
|  | OH | 8.9 | 14.3 | 0.17 | 0.08 | 12.0 | 0.10 | 0.03 | 10.1 | 0.04 | 0.15 | 10.9 | 0.07 | 0.13 |
|  | PA | 7.2 | 7.7 | 0.02 | 0.07 | 9.1 | 0.07 | 0.04 | 5.3 | 0.08 | 0.11 | 4.1 | 0.14 | 0.12 |
| Calendar year of cohort entry | 2000 | 2.5 | 4.9 | 0.13 | 0.11 | 2.3 | 0.01 | 0.01 | 14.9 | 0.45 | 0.47 | 0.0 | 0.23 | 0.14 |
|  | 2001 | 3.6 | 5.4 | 0.08 | 0.07 | 3.3 | 0.02 | 0.01 | 22.8 | 0.59 | 0.73 | 0.0 | 0.27 | 0.19 |
|  | 2002 | 5.0 | 5.6 | 0.03 | 0.05 | 4.5 | 0.02 | 0.02 | 23.1 | 0.54 | 0.72 | 17.2 | 0.40 | 0.30 |
|  | 2003 | 6.6 | 6.0 | 0.02 | 0.08 | 6.1 | 0.02 | 0.02 | 23.9 | 0.50 | 0.84 | 38.3 | 0.82 | 0.82 |
|  | 2004 | 6.4 | 5.4 | 0.04 | 0.08 | 6.5 | 0.00 | 0.03 | 15.4 | 0.29 | 0.96 | 39.6 | 0.86 | 1.46 |
|  | 2005 | 10.8 | 12.2 | 0.04 | 0.06 | 10.0 | 0.03 | 0.02 | 0.0 | 0.49 | 0.37 | 4.8 | 0.22 | 0.30 |
|  | 2006 | 13.8 | 17.7 | 0.11 | 0.09 | 14.9 | 0.03 | 0.04 | 0.0 | 0.57 | 0.38 | 0.0 | 0.57 | 0.38 |
|  | 2007 | 13.0 | 13.5 | 0.01 | 0.05 | 14.0 | 0.03 | 0.03 | 0.0 | 0.55 | 0.37 | 0.0 | 0.55 | 0.37 |
|  | 2008 | 10.7 | 9.9 | 0.03 | 0.03 | 10.4 | 0.01 | 0.03 | 0.0 | 0.49 | 0.31 | 0.0 | 0.49 | 0.31 |
|  | 2009 | 13.1 | 9.9 | 0.10 | 0.07 | 13.5 | 0.01 | 0.02 | 0.0 | 0.55 | 0.35 | 0.0 | 0.55 | 0.35 |
|  | 2010 | 14.6 | 9.5 | 0.16 | 0.10 | 14.6 | 0.00 | 0.04 | 0.0 | 0.58 | 0.37 | 0.0 | 0.58 | 0.37 |
| Medicaid-Medicare  dual-eligible | Yes | 68.3 | 75.9 | 0.17 | 0.12 | 70.1 | 0.04 | 0.03 | 79.4 | 0.26 | 0.82 | 78.9 | 0.24 | 0.77 |
| ***Healthcare utilization factors during the one-year baseline time*** | | | | | | | | | | | | | | |
| Nursing home residence | Yes | 10.2 | 8.0 | 0.08 | 0.07 | 8.4 | 0.06 | 0.04 | 15.0 | 0.15 | 0.24 | 10.6 | 0.01 | 0.24 |
| Number of circulatory system hospitalizations | Median (Q3-Q1) | 0.0  (0.0-1.0) | 0.0 (0.0-1.0) | 0.07 | 0.10 | 0.0 (0.0-1.0) | 0.03 | 0.05 | 0.0 (0.0-1.0) | 0.11 | 0.19 | 0.0 (0.0-1.0) | 0.07 | 0.16 |
| Number of non-circulatory system hospitalizations | Median (Q3-Q1) | 0.0  (0.0-1.0) | 0.0 (0.0-0.0) | 0.12 | 0.11 | 0.0 (0.0-1.0) | 0.05 | 0.05 | 0.0 (0.0-1.0) | 0.03 | 0.22 | 0.0 (0.0-1.0) | 0.06 | 0.15 |
| Number of circulatory system ED^‡^ visits | Median (Q3-Q1) | 0.0  (0.0-1.0) | 0.0 (0.0-0.0) | 0.01 | 0.03 | 0.0 (0.0-0.0) | 0.02 | 0.02 | 0.0 (0.0-1.0) | 0.06 | 0.03 | 0.0 (0.0-0.0) | 0.13 | 0.11 |
| Number of non-circulatory system ED visits | Median (Q3-Q1) | 1.0  (0.0-2.0) | 0.0 (0.0-2.0) | 0.06 | 0.06 | 1.0 (0.0-2.0) | 0.04 | 0.03 | 1.0 (0.0-2.0) | 0.00 | 0.06 | 0.0 (0.0-2.0) | 0.01 | 0.07 |
| Number of circulatory system outpatient visits | Median (Q3-Q1) | 6.0  (2.0-11.0) | 5.0 (2.0-10.0) | 0.02 | 0.05 | 5.0 (2.0-11.0) | 0.01 | 0.03 | 8.0 (4.0-14.0) | 0.08 | 0.13 | 7.0 (4.0-13.0) | 0.13 | 0.11 |
| Number of non-circulatory system outpatient visits | Median (Q3-Q1) | 21.0  (10.0-41.0) | 21.0 (9.5-40.0) | 0.00 | 0.04 | 20.0 (9.0-38.0) | 0.03 | 0.04 | 25.0 (14.0-45.0) | 0.02 | 0.28 | 28.0 (16.0-51.0) | 0.12 | 0.12 |
| Number of prescription dispensings | Median (Q3-Q1) | 70.0  (41.0-109) | 72.0 (41.0-112) | 0.04 | 0.06 | 68.0 (39.0-107) | 0.02 | 0.05 | 69.0 (43.0-104) | 0.03 | 0.43 | 76.0 (48.0-115) | 0.11 | 0.24 |
| Number of unique prescriptions | Median (Q3-Q1) | 17.0  (11.0-24.0) | 17.0 (12.0-24.0) | 0.02 | 0.07 | 17.0 (11.0-23.0) | 0.04 | 0.05 | 18.0 (12.0-25.0) | 0.11 | 0.41 | 19.0 (14.0-26.0) | 0.24 | 0.18 |
| Number of inpatient diagnosis codes | Median (Q3-Q1) | 0.0  (0.0-9.0) | 0.0 (0.0-9.0) | 0.09 | 0.11 | 0.0 (0.0-9.0) | 0.03 | 0.05 | 3.0 (0.0-9.0) | 0.06 | 0.30 | 0.0 (0.0-9.0) | 0.07 | 0.21 |
| Number of unique inpatient diagnosis codes | Median (Q3-Q1) | 0.0  (0.0-8.0) | 0.0 (0.0-8.0) | 0.07 | 0.10 | 0.0 (0.0-8.0) | 0.02 | 0.04 | 3.0 (0.0-9.0) | 0.10 | 0.37 | 0.0 (0.0-8.0) | 0.05 | 0.26 |
| Number of inpatient ICD-9^§^ procedure codes | Median (Q3-Q1) | 0.0  (0.0-2.0) | 0.0 (0.0-2.0) | 0.04 | 0.08 | 0.0 (0.0-2.0) | 0.02 | 0.05 | 0.0 (0.0-3.0) | 0.05 | 0.22 | 0.0 (0.0-2.0) | 0.07 | 0.15 |
| Number of unique inpatient ICD-9 procedure codes | Median (Q3-Q1) | 0.0  (0.0-1.0) | 0.0 (0.0-1.0) | 0.03 | 0.09 | 0.0 (0.0-1.0) | 0.01 | 0.04 | 0.0 (0.0-2.0) | 0.05 | 0.25 | 0.0 (0.0-1.0) | 0.06 | 0.17 |
| Number of inpatient CPT-4^‖^/HCPCS^#^ procedure codes | Median (Q3-Q1) | 0.0  (0.0-0.0) | 0.0 (0.0-0.0) | 0.03 | 0.05 | 0.0 (0.0-0.0) | 0.02 | 0.02 | 0.0 (0.0-0.0) | 0.12 | 0.22 | 0.0 (0.0-0.0) | 0.02 | 0.06 |
| Number of unique inpatient CPT-4/HCPCS procedure codes | Median (Q3-Q1) | 0.0  (0.0-0.0) | 0.0 (0.0-0.0) | 0.03 | 0.05 | 0.0 (0.0-0.0) | 0.02 | 0.02 | 0.0 (0.0-0.0) | 0.12 | 0.22 | 0.0 (0.0-0.0) | 0.02 | 0.06 |
| Number of outpatient diagnosis codes | Median (Q3-Q1) | 63.0  (29.0-127) | 62.0 (28.0-123) | 0.02 | 0.07 | 59.0 (27.0-117) | 0.05 | 0.06 | 78.0 (44.0-138) | 0.08 | 0.41 | 86.0 (48.0-157) | 0.18 | 0.23 |
| Number of unique outpatient diagnosis codes | Median (Q3-Q1) | 22.0  (12.0-33.0) | 22.0 (12.0-33.0) | 0.02 | 0.12 | 21.0 (12.0-32.0) | 0.05 | 0.07 | 26.0 (17.0-37.0) | 0.27 | 0.72 | 28.0 (18.0-38.0) | 0.32 | 0.42 |
| Number of outpatient ICD-9 procedure codes | Median (Q3-Q1) | 0.0  (0.0-0.0) | 0.0 (0.0-0.0) | 0.01 | 0.05 | 0.0 (0.0-0.0) | 0.02 | 0.03 | 0.0 (0.0-1.0) | 0.37 | 0.28 | 0.0 (0.0-1.0) | 0.48 | 0.27 |
| Number of unique outpatient ICD-9 procedure codes | Median (Q3-Q1) | 0.0  (0.0-0.0) | 0.0 (0.0-0.0) | 0.00 | 0.06 | 0.0 (0.0-0.0) | 0.03 | 0.03 | 0.0 (0.0-1.0) | 0.55 | 0.37 | 0.0 (0.0-1.0) | 0.63 | 0.28 |
| Number of outpatient CPT-4/HCPCS procedure codes | Median (Q3-Q1) | 75.0  (35.0-145) | 72.0 (32.0-134) | 0.10 | 0.11 | 70.0 (32.0-133) | 0.08 | 0.07 | 89.0 (48.0-155) | 0.02 | 0.50 | 95.0 (51.0-166) | 0.06 | 0.33 |
| Number of unique outpatient CPT-4/HCPCS procedure codes | Median (Q3-Q1) | 41.0  (22.0-63.0) | 39.5 (20.0-61.0) | 0.07 | 0.13 | 39.0 (20.0-61.0) | 0.07 | 0.07 | 45.0 (28.0-65.0) | 0.12 | 0.66 | 47.0 (30.0-67.0) | 0.17 | 0.37 |
| Number of other diagnosis codes | Median (Q3-Q1) | 0.0  (0.0-0.0) | 0.0 (0.0-0.0) | 0.06 | 0.07 | 0.0 (0.0-0.0) | 0.03 | 0.04 | 0.0 (0.0-0.0) | 0.09 | 0.20 | 0.0 (0.0-0.0) | 0.09 | 0.15 |
| Number of unique other diagnosis codes | Median (Q3-Q1) | 0.0  (0.0-0.0) | 0.0 (0.0-0.0) | 0.04 | 0.07 | 0.0 (0.0-0.0) | 0.03 | 0.03 | 0.0 (0.0-0.0) | 0.07 | 0.36 | 0.0 (0.0-0.0) | 0.00 | 0.27 |
| Number of other ICD-9 procedure codes | Median (Q3-Q1) | 0.0  (0.0-0.0) | 0.0 (0.0-0.0) | 0.01 | 0.05 | 0.0 (0.0-0.0) | 0.01 | 0.03 | 0.0 (0.0-0.0) | 0.02 | 0.06 | 0.0 (0.0-0.0) | 0.00 | 0.06 |
| Number of unique other ICD-9 procedure codes | Median (Q3-Q1) | 0.0  (0.0-0.0) | 0.0 (0.0-0.0) | 0.01 | 0.04 | 0.0 (0.0-0.0) | 0.01 | 0.03 | 0.0 (0.0-0.0) | 0.03 | 0.06 | 0.0 (0.0-0.0) | 0.01 | 0.06 |
| ***Diseases during the one-year baseline time*** | | | | | | | | | | | | | | |
| AMI*** | Yes | 8.8 | 7.8 | 0.04 | 0.03 | 7.8 | 0.03 | 0.02 | 8.3 | 0.02 | 0.06 | 6.6 | 0.08 | 0.07 |
| GIB*** | Yes | 1.5 | 1.5 | 0.00 | 0.03 | 1.5 | 0.00 | 0.04 | 2.7 | 0.09 | 0.07 | 2.1 | 0.05 | 0.06 |
| Ischemic stroke*** | Yes | 4.0 | 3.6 | 0.03 | 0.05 | 3.8 | 0.01 | 0.01 | 6.7 | 0.12 | 0.09 | 4.0 | 0.00 | 0.09 |
| Non-traumatic ICH*** | Yes | 0.3 | 0.2 | 0.01 | 0.03 | 0.2 | 0.01 | 0.02 | 0.3 | 0.01 | 0.02 | 0.2 | 0.01 | 0.03 |
| Acute respiratory infection | Yes | 10.2 | 10.4 | 0.01 | 0.04 | 10.0 | 0.01 | 0.02 | 12.0 | 0.06 | 0.07 | 13.2 | 0.09 | 0.05 |
| Alcohol abuse | Yes | 3.7 | 2.7 | 0.06 | 0.06 | 3.2 | 0.03 | 0.03 | 2.9 | 0.05 | 0.07 | 2.8 | 0.06 | 0.10 |
| Angina pectoris | Yes | 22.2 | 21.6 | 0.02 | 0.06 | 21.5 | 0.02 | 0.04 | 29.0 | 0.16 | 0.09 | 29.0 | 0.16 | 0.06 |
| Artery disease | Yes | 34.7 | 34.1 | 0.01 | 0.06 | 33.1 | 0.03 | 0.04 | 38.4 | 0.08 | 0.30 | 40.0 | 0.11 | 0.21 |
| Asthma/COPD/emphysema | Yes | 32.1 | 34.0 | 0.04 | 0.03 | 32.0 | 0.00 | 0.04 | 35.5 | 0.07 | 0.20 | 36.8 | 0.10 | 0.12 |
| Atrial fibrillation | Yes | 9.2 | 8.5 | 0.03 | 0.05 | 8.5 | 0.03 | 0.02 | 12.9 | 0.12 | 0.16 | 11.5 | 0.08 | 0.15 |
| Cancer | Yes | 11.9 | 11.6 | 0.01 | 0.04 | 11.5 | 0.01 | 0.02 | 15.8 | 0.11 | 0.15 | 16.2 | 0.12 | 0.11 |
| Cardiac dysrhythmias | Yes | 22.8 | 20.8 | 0.05 | 0.05 | 21.4 | 0.03 | 0.03 | 29.3 | 0.15 | 0.16 | 26.9 | 0.09 | 0.11 |
| Cardiovascular system symptoms | Yes | 23.8 | 23.2 | 0.01 | 0.05 | 22.0 | 0.04 | 0.03 | 27.2 | 0.08 | 0.19 | 28.6 | 0.11 | 0.14 |
| Cerebrovascular disease: hemorrhage | Yes | 1.1 | 0.8 | 0.03 | 0.06 | 0.9 | 0.02 | 0.02 | 1.6 | 0.04 | 0.04 | 1.0 | 0.01 | 0.06 |
| Cerebrovascular disease: ischemic stroke | Yes | 18.7 | 16.7 | 0.05 | 0.07 | 17.6 | 0.03 | 0.03 | 27.3 | 0.21 | 0.16 | 21.7 | 0.07 | 0.14 |
| Cerebrovascular disease: other | Yes | 26.1 | 25.0 | 0.03 | 0.05 | 24.8 | 0.03 | 0.03 | 32.9 | 0.15 | 0.21 | 29.5 | 0.08 | 0.15 |
| Cerebrovascular disease: transient cerebral ischemia | Yes | 12.0 | 12.8 | 0.03 | 0.05 | 12.1 | 0.00 | 0.03 | 19.3 | 0.20 | 0.08 | 15.8 | 0.11 | 0.11 |
| Circulatory system disease: other | Yes | 89.5 | 86.6 | 0.09 | 0.10 | 88.2 | 0.04 | 0.03 | 94.7 | 0.20 | 0.37 | 95.4 | 0.23 | 0.18 |
| Conduction disorders | Yes | 6.0 | 5.4 | 0.03 | 0.05 | 6.1 | 0.00 | 0.03 | 7.7 | 0.07 | 0.09 | 6.0 | 0.00 | 0.08 |
| Congenital anomalies of heart | Yes | 4.4 | 3.5 | 0.05 | 0.04 | 3.8 | 0.03 | 0.02 | 6.0 | 0.07 | 0.04 | 5.6 | 0.05 | 0.05 |
| Diabetes mellitus | Yes | 52.3 | 49.3 | 0.06 | 0.06 | 50.8 | 0.03 | 0.03 | 48.8 | 0.07 | 0.28 | 50.9 | 0.03 | 0.13 |
| Fever | Yes | 2.6 | 1.7 | 0.06 | 0.04 | 2.1 | 0.03 | 0.01 | 2.5 | 0.00 | 0.07 | 2.0 | 0.04 | 0.07 |
| Gingival and periodontal disease | Yes | 0.3 | 0.2 | 0.03 | 0.03 | 0.2 | 0.02 | 0.02 | 0.2 | 0.03 | 0.03 | 0.3 | 0.01 | 0.03 |
| Heart failure | Yes | 30.4 | 27.9 | 0.06 | 0.04 | 28.1 | 0.05 | 0.03 | 37.1 | 0.14 | 0.21 | 34.8 | 0.09 | 0.17 |
| HIV/AIDS | Yes | 2.0 | 0.9 | 0.10 | 0.05 | 1.5 | 0.04 | 0.02 | 0.7 | 0.11 | 0.05 | 0.6 | 0.12 | 0.04 |
| Hypertension | Yes | 81.0 | 78.9 | 0.05 | 0.09 | 80.2 | 0.02 | 0.04 | 86.7 | 0.15 | 0.28 | 88.7 | 0.21 | 0.14 |
| Hypothyroidism | Yes | 16.3 | 18.7 | 0.06 | 0.04 | 16.4 | 0.00 | 0.03 | 21.3 | 0.13 | 0.18 | 22.8 | 0.16 | 0.15 |
| Infection: other serious | Yes | 15.2 | 14.2 | 0.03 | 0.04 | 13.8 | 0.04 | 0.02 | 18.6 | 0.09 | 0.19 | 18.0 | 0.08 | 0.13 |
| Infectious and parasitic diseases: cytomegaloviral | Yes | 0.0 | 0.0 | 0.02 | 0.02 | 0.0 | 0.01 | 0.01 | 0.0 | 0.03 | 0.02 | 0.0 | 0.03 | 0.01 |
| Infectious and parasitic diseases: Helicobacter pylori | Yes | 0.2 | 0.3 | 0.02 | 0.04 | 0.2 | 0.01 | 0.01 | 0.3 | 0.02 | 0.02 | 0.3 | 0.01 | 0.03 |
| Infectious and parasitic diseases: herpes simplex | Yes | 0.2 | 0.2 | 0.01 | 0.03 | 0.2 | 0.00 | 0.01 | 0.3 | 0.02 | 0.03 | 0.2 | 0.00 | 0.02 |
| Infectious and parasitic diseases: other | Yes | 19.9 | 17.1 | 0.07 | 0.06 | 17.9 | 0.05 | 0.02 | 23.6 | 0.09 | 0.22 | 23.1 | 0.08 | 0.15 |
| Ischemic heart disease | Yes | 60.4 | 55.9 | 0.09 | 0.09 | 59.5 | 0.02 | 0.04 | 64.0 | 0.07 | 0.21 | 63.6 | 0.07 | 0.12 |
| Lipoid metabolism disorder | Yes | 64.0 | 65.0 | 0.02 | 0.09 | 64.3 | 0.01 | 0.04 | 61.2 | 0.06 | 0.24 | 70.4 | 0.14 | 0.09 |
| Liver diseases | Yes | 12.4 | 10.4 | 0.06 | 0.05 | 11.0 | 0.04 | 0.01 | 12.1 | 0.01 | 0.17 | 12.8 | 0.01 | 0.11 |
| Mental disorder: depression | Yes | 24.1 | 23.4 | 0.02 | 0.04 | 22.2 | 0.05 | 0.05 | 25.9 | 0.04 | 0.20 | 28.4 | 0.10 | 0.08 |
| Mental disorder: other | Yes | 36.7 | 34.0 | 0.06 | 0.04 | 34.1 | 0.05 | 0.03 | 36.0 | 0.02 | 0.22 | 35.2 | 0.03 | 0.14 |
| Myocardial infarction: acute | Yes | 14.3 | 11.8 | 0.08 | 0.05 | 12.9 | 0.04 | 0.02 | 14.8 | 0.01 | 0.08 | 12.1 | 0.07 | 0.07 |
| Myocardial infarction: old | Yes | 11.3 | 9.1 | 0.07 | 0.05 | 11.0 | 0.01 | 0.03 | 10.3 | 0.03 | 0.11 | 9.4 | 0.06 | 0.10 |
| Nervous system disease: disorders of the eye and adnexa | Yes | 50.4 | 51.8 | 0.03 | 0.07 | 49.2 | 0.02 | 0.02 | 60.8 | 0.21 | 0.24 | 63.1 | 0.26 | 0.13 |
| Nervous system disease (central): hereditary and degenerative | Yes | 10.3 | 10.2 | 0.00 | 0.03 | 9.5 | 0.03 | 0.02 | 13.8 | 0.11 | 0.18 | 13.6 | 0.10 | 0.16 |
| Nervous system disease (central): inflammatory disease | Yes | 0.3 | 0.3 | 0.01 | 0.02 | 0.3 | 0.01 | 0.02 | 0.5 | 0.03 | 0.02 | 0.4 | 0.01 | 0.03 |
| Nervous system disease (central): other | Yes | 13.9 | 12.4 | 0.05 | 0.07 | 12.8 | 0.03 | 0.03 | 17.1 | 0.09 | 0.14 | 14.6 | 0.02 | 0.09 |
| Nervous system disease (peripheral) | Yes | 16.5 | 17.6 | 0.03 | 0.05 | 16.4 | 0.00 | 0.03 | 18.3 | 0.05 | 0.19 | 20.4 | 0.10 | 0.14 |
| Obesity | Yes | 10.7 | 11.3 | 0.02 | 0.03 | 10.7 | 0.00 | 0.02 | 8.3 | 0.08 | 0.13 | 9.8 | 0.03 | 0.07 |
| Osteoarthritis | Yes | 31.4 | 40.1 | 0.18 | 0.13 | 34.5 | 0.07 | 0.07 | 49.5 | 0.38 | 0.36 | 54.8 | 0.49 | 0.29 |
| Pulmonary congestion and hypostasis | Yes | 4.6 | 3.7 | 0.04 | 0.06 | 4.2 | 0.02 | 0.02 | 5.1 | 0.02 | 0.11 | 4.2 | 0.02 | 0.10 |
| Pacemaker/ICD** | Yes | 1.1 | 1.0 | 0.01 | 0.03 | 1.0 | 0.01 | 0.02 | 1.3 | 0.01 | 0.05 | 1.0 | 0.01 | 0.05 |
| Pneumonia | Yes | 4.6 | 4.1 | 0.02 | 0.03 | 4.2 | 0.02 | 0.03 | 6.7 | 0.09 | 0.09 | 4.8 | 0.01 | 0.09 |
| Renal disease: chronic | Yes | 9.6 | 6.9 | 0.10 | 0.08 | 8.8 | 0.03 | 0.03 | 7.0 | 0.10 | 0.17 | 5.4 | 0.16 | 0.17 |
| Renal disease: other | Yes | 17.5 | 14.6 | 0.08 | 0.08 | 16.0 | 0.04 | 0.04 | 19.2 | 0.04 | 0.18 | 19.8 | 0.06 | 0.11 |
| Renal failure: acute | Yes | 5.3 | 4.0 | 0.06 | 0.04 | 4.7 | 0.03 | 0.02 | 4.3 | 0.05 | 0.12 | 3.4 | 0.09 | 0.11 |
| Rheumatoid arthritis | Yes | 5.0 | 7.2 | 0.09 | 0.05 | 5.3 | 0.01 | 0.04 | 7.5 | 0.10 | 0.15 | 8.2 | 0.13 | 0.13 |
| Stent placement | Yes | 12.4 | 13.5 | 0.03 | 0.05 | 13.0 | 0.02 | 0.03 | 11.5 | 0.03 | 0.10 | 10.6 | 0.06 | 0.09 |
| Substance abuse | Yes | 13.2 | 11.4 | 0.05 | 0.05 | 12.7 | 0.01 | 0.02 | 7.6 | 0.18 | 0.09 | 7.5 | 0.19 | 0.10 |
| Tobacco use | Yes | 16.4 | 15.0 | 0.04 | 0.04 | 16.3 | 0.00 | 0.02 | 10.4 | 0.18 | 0.11 | 10.4 | 0.18 | 0.11 |
| Urinary tract infection | Yes | 11.1 | 10.7 | 0.01 | 0.05 | 10.2 | 0.03 | 0.01 | 14.3 | 0.10 | 0.18 | 14.0 | 0.09 | 0.12 |
| ***Drugs during the one-year baseline time*** | | | | | | | | | | | | | | |
| Agents for migraine | Yes | 1.7 | 2.5 | 0.06 | 0.04 | 1.8 | 0.01 | 0.02 | 1.6 | 0.01 | 0.05 | 2.2 | 0.04 | 0.05 |
| Antiadrenergic agents | Yes | 16.4 | 14.2 | 0.06 | 0.07 | 15.3 | 0.03 | 0.03 | 16.2 | 0.01 | 0.10 | 17.0 | 0.02 | 0.09 |
| Antialcohol agents | Yes | 0.1 | 🞵🞵 | 🞵🞵 | 🞵🞵 | 0.1 | 0.01 | 0.02 | 0.0 | 0.03 | 0.03 | 0.0 | 0.02 | 0.03 |
| Antiarrhythmic agents | Yes | 2.6 | 2.2 | 0.03 | 0.05 | 2.2 | 0.03 | 0.02 | 3.3 | 0.04 | 0.05 | 2.8 | 0.01 | 0.07 |
| Anticoagulants | Yes | 5.3 | 4.9 | 0.02 | 0.03 | 4.8 | 0.02 | 0.02 | 7.2 | 0.08 | 0.12 | 6.3 | 0.04 | 0.10 |
| Anticonvulsants | Yes | 8.8 | 9.0 | 0.01 | 0.04 | 8.5 | 0.01 | 0.04 | 7.6 | 0.04 | 0.08 | 7.4 | 0.05 | 0.06 |
| Antidepressants | Yes | 40.0 | 43.9 | 0.08 | 0.04 | 39.6 | 0.01 | 0.03 | 42.5 | 0.05 | 0.21 | 45.8 | 0.12 | 0.09 |
| Antidepressants: monoamine oxidase inhibitors | Yes | 0.0 | 0.0 | 0.01 | 0.01 | 0.0 | 0.01 | 0.01 | 0.0 | 0.00 | 0.03 | 0.0 | 0.01 | 0.01 |
| Antidepressants: other | Yes | 9.8 | 11.0 | 0.04 | 0.03 | 9.8 | 0.00 | 0.02 | 9.1 | 0.02 | 0.05 | 9.8 | 0.00 | 0.06 |
| Antidepressants: selective serotonin reuptake inhibitors | Yes | 27.0 | 29.0 | 0.04 | 0.04 | 26.1 | 0.02 | 0.03 | 28.6 | 0.04 | 0.16 | 32.4 | 0.12 | 0.08 |
| Antidepressants: serotonin and norepinephrine reuptake inhibitors | Yes | 5.4 | 6.9 | 0.06 | 0.03 | 5.5 | 0.00 | 0.02 | 3.4 | 0.10 | 0.09 | 4.2 | 0.06 | 0.08 |
| Antidepressants: tetracyclic | Yes | 3.3 | 3.1 | 0.01 | 0.03 | 2.9 | 0.02 | 0.03 | 3.6 | 0.02 | 0.07 | 4.3 | 0.06 | 0.07 |
| Antidepressants: tricyclic | Yes | 8.4 | 10.0 | 0.05 | 0.04 | 9.1 | 0.02 | 0.03 | 12.3 | 0.13 | 0.09 | 11.1 | 0.09 | 0.05 |
| Antidiabetic agents: insulin | Yes | 18.8 | 15.7 | 0.08 | 0.04 | 17.3 | 0.04 | 0.03 | 13.5 | 0.14 | 0.21 | 12.6 | 0.17 | 0.15 |
| Antidiabetic agents: non-insulin | Yes | 36.6 | 35.6 | 0.02 | 0.04 | 36.8 | 0.00 | 0.02 | 30.4 | 0.13 | 0.15 | 32.1 | 0.09 | 0.08 |
| Anti-infectives | Yes | 71.5 | 69.6 | 0.04 | 0.06 | 68.9 | 0.06 | 0.04 | 73.3 | 0.04 | 0.19 | 73.7 | 0.05 | 0.12 |
| Anti-infectives in 7 days prior to cohort entry | Yes | 7.0 | 4.3 | 0.12 | 0.04 | 5.0 | 0.09 | 0.03 | 4.9 | 0.09 | 0.09 | 4.7 | 0.10 | 0.07 |
| Antiobesity agents | Yes | 0.1 | 🞵🞵 | 🞵🞵 | 🞵🞵 | 0.1 | 0.01 | 0.02 | 0.1 | 0.01 | 0.03 | 🞵🞵 | 🞵🞵 | 🞵🞵 |
| Antiplatelet agents excluding clopidogrel | Yes | 5.2 | 6.0 | 0.04 | 0.03 | 5.2 | 0.00 | 0.01 | 7.2 | 0.08 | 0.04 | 7.9 | 0.11 | 0.06 |
| Antipsychotics | Yes | 15.5 | 13.3 | 0.06 | 0.05 | 13.1 | 0.07 | 0.04 | 13.7 | 0.05 | 0.16 | 13.8 | 0.05 | 0.10 |
| Antiretroviral agents | Yes | 1.6 | 0.6 | 0.10 | 0.06 | 1.2 | 0.04 | 0.02 | 0.5 | 0.10 | 0.05 | 0.5 | 0.11 | 0.03 |
| Aspirin | Yes | 37.9 | 30.0 | 0.17 | 0.08 | 33.5 | 0.09 | 0.03 | 40.2 | 0.05 | 0.15 | 41.3 | 0.07 | 0.18 |
| Benzisoxazoles | Yes | 4.7 | 4.2 | 0.02 | 0.04 | 3.8 | 0.04 | 0.03 | 4.4 | 0.01 | 0.10 | 4.4 | 0.01 | 0.09 |
| Beta-adrenergic agents and alpha/beta-adrenergic blocking agents | Yes | 58.3 | 53.3 | 0.10 | 0.06 | 57.7 | 0.01 | 0.04 | 47.3 | 0.22 | 0.11 | 52.2 | 0.12 | 0.11 |
| Bronchodilators/inhaled corticosteroids | Yes | 30.7 | 32.1 | 0.03 | 0.03 | 30.5 | 0.00 | 0.03 | 29.1 | 0.04 | 0.14 | 30.4 | 0.01 | 0.07 |
| Calcium channel blockers | Yes | 38.9 | 39.9 | 0.02 | 0.05 | 38.9 | 0.00 | 0.01 | 46.1 | 0.15 | 0.06 | 43.5 | 0.09 | 0.05 |
| CYP1A2 inducers | Yes | 35.3 | 35.3 | 0.00 | 0.05 | 35.1 | 0.00 | 0.04 | 27.1 | 0.18 | 0.23 | 28.9 | 0.14 | 0.17 |
| CYP1A2 inhibitors | Yes | 11.5 | 11.9 | 0.01 | 0.04 | 11.5 | 0.00 | 0.02 | 13.1 | 0.05 | 0.08 | 11.3 | 0.01 | 0.09 |
| CYP2B6 inducers | Yes | 2.3 | 1.8 | 0.04 | 0.03 | 2.0 | 0.02 | 0.02 | 2.3 | 0.00 | 0.03 | 1.8 | 0.03 | 0.03 |
| CYP2B6 inhibitors | Yes | 0.1 | 🞵🞵 | 🞵🞵 | 🞵🞵 | 0.1 | 0.00 | 0.02 | 0.3 | 0.05 | 0.03 | 🞵🞵 | 🞵🞵 | 🞵🞵 |
| CYP2C19 inducers | Yes | 5.8 | 5.3 | 0.02 | 0.05 | 5.6 | 0.01 | 0.02 | 5.4 | 0.01 | 0.08 | 5.4 | 0.01 | 0.05 |
| CYP2C19 inhibitors | Yes | 28.9 | 31.8 | 0.06 | 0.05 | 28.7 | 0.00 | 0.04 | 28.3 | 0.01 | 0.20 | 33.1 | 0.09 | 0.12 |
| CYP2C9 inducers | Yes | 1.8 | 1.7 | 0.01 | 0.03 | 1.7 | 0.01 | 0.02 | 2.0 | 0.01 | 0.03 | 1.6 | 0.02 | 0.03 |
| CYP2C9 inhibitors | Yes | 15.8 | 15.5 | 0.01 | 0.03 | 15.3 | 0.01 | 0.03 | 14.2 | 0.04 | 0.11 | 15.8 | 0.00 | 0.07 |
| CYP3A4/5 inducers | Yes | 12.2 | 11.8 | 0.01 | 0.03 | 12.1 | 0.00 | 0.02 | 10.6 | 0.05 | 0.10 | 11.7 | 0.01 | 0.08 |
| CYP3A4/5 inhibitors | Yes | 42.7 | 41.8 | 0.02 | 0.02 | 41.9 | 0.02 | 0.02 | 42.2 | 0.01 | 0.11 | 43.3 | 0.01 | 0.08 |
| Dibenzazepines | Yes | 8.4 | 6.9 | 0.06 | 0.06 | 6.9 | 0.06 | 0.03 | 6.1 | 0.09 | 0.15 | 8.0 | 0.01 | 0.09 |
| Dipyridamole | Yes | 1.4 | 1.7 | 0.03 | 0.03 | 1.6 | 0.01 | 0.02 | 1.8 | 0.03 | 0.06 | 1.9 | 0.04 | 0.03 |
| Diuretics: loop | Yes | 25.7 | 26.4 | 0.01 | 0.02 | 25.6 | 0.00 | 0.02 | 29.3 | 0.08 | 0.16 | 28.9 | 0.07 | 0.13 |
| Diuretics: other | Yes | 33.0 | 34.8 | 0.04 | 0.02 | 33.9 | 0.02 | 0.01 | 31.9 | 0.02 | 0.07 | 37.6 | 0.10 | 0.09 |
| Estrogens | Yes | 5.2 | 7.7 | 0.10 | 0.03 | 5.8 | 0.02 | 0.02 | 11.4 | 0.23 | 0.09 | 9.4 | 0.16 | 0.08 |
| Fibrates | Yes | 7.8 | 8.2 | 0.01 | 0.04 | 7.9 | 0.00 | 0.02 | 4.9 | 0.12 | 0.07 | 5.7 | 0.08 | 0.05 |
| H2-receptor antagonists | Yes | 18.0 | 19.0 | 0.02 | 0.04 | 17.9 | 0.00 | 0.02 | 24.0 | 0.15 | 0.07 | 17.4 | 0.02 | 0.11 |
| Influenza vaccination | Yes | 23.0 | 24.9 | 0.05 | 0.09 | 24.0 | 0.02 | 0.03 | 28.9 | 0.14 | 0.34 | 30.7 | 0.18 | 0.32 |
| Inotropic agents | Yes | 6.2 | 6.3 | 0.00 | 0.04 | 5.7 | 0.02 | 0.02 | 10.8 | 0.16 | 0.05 | 8.2 | 0.08 | 0.07 |
| Leukotriene formation inhibitors and leukotriene receptor antagonists | Yes | 7.9 | 8.9 | 0.04 | 0.04 | 7.9 | 0.00 | 0.02 | 6.7 | 0.05 | 0.09 | 9.2 | 0.04 | 0.06 |
| Non-study NSAIDs | Yes | 1.4 | 2.6 | 0.08 | 0.04 | 1.7 | 0.02 | 0.02 | 2.1 | 0.05 | 0.04 | 2.0 | 0.05 | 0.03 |
| Oral contraceptives | Yes | 0.6 | 0.5 | 0.01 | 0.03 | 0.5 | 0.00 | 0.02 | 0.6 | 0.00 | 0.05 | 0.8 | 0.02 | 0.09 |
| Phenothiazines | Yes | 3.2 | 3.0 | 0.01 | 0.04 | 2.8 | 0.02 | 0.02 | 4.3 | 0.06 | 0.04 | 2.8 | 0.03 | 0.07 |
| Phenylbutylpiperidines | Yes | 1.1 | 0.8 | 0.03 | 0.04 | 0.7 | 0.04 | 0.01 | 1.2 | 0.01 | 0.03 | 0.6 | 0.05 | 0.05 |
| Potassium supplements | Yes | 15.8 | 17.0 | 0.03 | 0.03 | 16.0 | 0.00 | 0.02 | 18.9 | 0.08 | 0.13 | 18.0 | 0.06 | 0.13 |
| Proton pump inhibitors | Yes | 47.6 | 51.8 | 0.08 | 0.06 | 47.7 | 0.00 | 0.04 | 50.2 | 0.05 | 0.22 | 57.0 | 0.19 | 0.18 |
| Quinolinones | Yes | 2.1 | 1.6 | 0.04 | 0.03 | 1.7 | 0.03 | 0.02 | 0.1 | 0.19 | 0.08 | 0.4 | 0.15 | 0.06 |
| Ranolazine | Yes | 0.6 | 0.4 | 0.03 | 0.03 | 0.6 | 0.01 | 0.02 | 0.0 | 0.11 | 0.05 | 0.0 | 0.11 | 0.05 |
| Renin angiotensin system antagonists | Yes | 67.7 | 64.6 | 0.07 | 0.05 | 67.3 | 0.01 | 0.03 | 61.2 | 0.14 | 0.12 | 66.6 | 0.02 | 0.07 |
| Statins | Yes | 67.5 | 65.1 | 0.05 | 0.06 | 67.6 | 0.00 | 0.02 | 50.8 | 0.34 | 0.17 | 58.3 | 0.19 | 0.11 |
| Thiazides diuretics | Yes | 30.4 | 32.6 | 0.05 | 0.02 | 31.6 | 0.03 | 0.01 | 29.7 | 0.02 | 0.07 | 35.5 | 0.11 | 0.10 |
| Thiazolidinediones | Yes | 14.6 | 15.0 | 0.01 | 0.03 | 14.9 | 0.01 | 0.02 | 12.7 | 0.06 | 0.10 | 16.3 | 0.05 | 0.08 |
| Thioxanthines | Yes | 0.1 | 🞵🞵 | 🞵🞵 | 🞵🞵 | 0.1 | 0.00 | 0.02 | 0.1 | 0.01 | 0.03 | 🞵🞵 | 🞵🞵 | 🞵🞵 |
| Thyroid hormones | Yes | 10.8 | 13.4 | 0.08 | 0.04 | 11.5 | 0.02 | 0.02 | 12.2 | 0.04 | 0.10 | 12.0 | 0.04 | 0.10 |
| Varenicline | Yes | 1.2 | 1.5 | 0.02 | 0.03 | 1.3 | 0.01 | 0.02 | 0.0 | 0.16 | 0.09 | 0.0 | 0.16 | 0.09 |
| Vasodilators | Yes | 34.6 | 33.5 | 0.02 | 0.05 | 33.6 | 0.02 | 0.02 | 40.3 | 0.12 | 0.09 | 37.4 | 0.06 | 0.08 |
| Warfarin | Yes | 4.6 | 4.4 | 0.01 | 0.03 | 4.2 | 0.02 | 0.02 | 6.5 | 0.08 | 0.11 | 6.0 | 0.06 | 0.09 |

AMI: acute myocardial infarction. GIB: gastrointestinal bleeding. ICH: intracranial hemorrhage. CYP: cytochrome P450.

*S.Diff: Standardized Difference vs. Ibuprofen. ^†^WCSD: Weighted Conditional Standardized Difference vs. Ibuprofen. ^‡^ED: emergency department. ^§^ICD-9: International Classification of Diseases 9th Revision. ^‖^CPT-4: Current Procedural Terminology 4th Edition. ^#^HCPCS: Healthcare Common Procedure Coding System. **ICD: Implantable Cardioverter Defibrillator. ***Measured using the same definitions used for the outcome ascertainment. 🞵🞵Numbers are suppressed by the cell size suppression policy of the Centers for Medicare and Medicaid Services.
