# Supplementary material for: Nonsteroidal anti-inflammatory drug choice and adverse outcomes in clopidogrel users: A retrospective cohort study
Source: PLoS One. 2018 Mar 14;13(3):e0193800. doi: 10.1371/journal.pone.0193800 (PMC5851628; doi:10.1371/journal.pone.0193800)
Supplement: S5 Table — A. Whole cohort with the follow-up time up to 180 days after cohort entry date. B. Excluding potential incomplete-data patients, without a restriction on the follow-up time. (DOCX) [file pone.0193800.s010.docx]

**S5 Table. Sensitivity analysis: Unadjusted incidence rates of outcomes by NSAID exposure group**

1. **Whole cohort with the follow-up time up to 180 days after cohort entry date**

| **Outcome** | **NSAID** | **Number of users** | **Number of events** | **Person-years** | **Rate per**  **1,000 p-ys*** | **95% CI**^†^ |
| --- | --- | --- | --- | --- | --- | --- |
| **All-Cause Mortality** | Overall | 268,114 | 2,119 | 40,721 | 52.0 | 49.8 – 54.3 |
|  | celecoxib | 66,317 | 732 | 12,310 | 59.5 | 55.2 – 63.9 |
|  | diclofenac | 18,593 | 129 | 2,910 | 44.3 | 37.0 – 52.7 |
|  | etodolac | 2,807 | 15 | 434 | 34.6 | 19.4 – 57.0 |
|  | ibuprofen | 69,779 | 380 | 7,958 | 47.8 | 43.1 – 52.8 |
|  | indomethacin | 7,651 | 56 | 791 | 70.8 | 53.5 – 91.9 |
|  | meloxicam | 25,459 | 158 | 4,426 | 35.7 | 30.4 – 41.7 |
|  | nabumetone | 7,060 | 44 | 1,136 | 38.7 | 28.1 – 52.0 |
|  | naproxen | 36,577 | 214 | 5,008 | 42.7 | 37.2 – 48.9 |
|  | rofecoxib | 26,247 | 348 | 4,460 | 78.0 | 70.1 – 86.7 |
|  | valdecoxib | 7,624 | 43 | 1,288 | 33.4 | 24.2 – 45.0 |
| **AMI^‡^/**  **Ischemic stroke** | Overall | 268,114 | 2,558 | 40,526 | 63.1 | 60.7 – 65.6 |
|  | celecoxib | 66,317 | 767 | 12,243 | 62.7 | 58.3 – 67.3 |
|  | diclofenac | 18,593 | 170 | 2,898 | 58.7 | 50.2 – 68.2 |
|  | etodolac | 2,807 | 31 | 431 | 72.0 | 48.9 – 102.2 |
|  | ibuprofen | 69,779 | 512 | 7,925 | 64.6 | 59.1 – 70.5 |
|  | indomethacin | 7,651 | 91 | 787 | 115.7 | 93.2 – 142.1 |
|  | meloxicam | 25,459 | 203 | 4,409 | 46.0 | 39.9 – 52.8 |
|  | nabumetone | 7,060 | 51 | 1,132 | 45.1 | 33.6 – 59.3 |
|  | naproxen | 36,577 | 284 | 4,990 | 56.9 | 50.5 – 63.9 |
|  | rofecoxib | 26,247 | 379 | 4,430 | 85.5 | 77.2 – 94.6 |
|  | valdecoxib | 7,624 | 70 | 1,284 | 54.5 | 42.5 – 68.9 |
| **GIB/ICH**^§^ | Overall | 268,091 | 2,350 | 40,545 | 58.0 | 55.6 – 60.4 |
|  | celecoxib | 66,310 | 632 | 12,258 | 51.6 | 47.6 – 55.7 |
|  | diclofenac | 18,592 | 186 | 2,894 | 64.3 | 55.4 – 74.2 |
|  | etodolac | 2,806 | 24 | 432 | 55.6 | 35.6 – 82.7 |
|  | ibuprofen | 69,775 | 393 | 7,935 | 49.5 | 44.8 – 54.7 |
|  | indomethacin | 7,651 | 104 | 786 | 132.3 | 108.1 – 160.4 |
|  | meloxicam | 25,457 | 222 | 4,408 | 50.4 | 44.0 – 57.4 |
|  | nabumetone | 7,060 | 37 | 1,133 | 32.7 | 23.0 – 45.0 |
|  | naproxen | 36,576 | 336 | 4,985 | 67.4 | 60.4 – 75.0 |
|  | rofecoxib | 26,241 | 368 | 4,431 | 83.1 | 74.8 – 92.0 |
|  | valdecoxib | 7,623 | 48 | 1,284 | 37.4 | 27.6 – 49.6 |

*p-ys: person-years. ^†^CI: confidence interval. **^‡^**AMI: acute myocardial infarction. ^§^GIB/ICH: gastrointestinal bleeding/Intracranial hemorrhage.

1. **Excluding potential incomplete-data patients, without a restriction on the follow-up time**

| **Outcome** | **NSAID** | **Number of users** | **Number of events** | **Person-years** | **Rate per**  **1,000 p-ys^*^** | **95% CI**^†^ |
| --- | --- | --- | --- | --- | --- | --- |
| **All-Cause Mortality** | Overall | 181,041 | 1,678 | 32,872 | 51.0 | 48.6 – 53.6 |
|  | celecoxib | 50,501 | 623 | 11,609 | 53.7 | 49.5 – 58.1 |
|  | diclofenac | 11,930 | 95 | 2,122 | 44.8 | 36.2 – 54.7 |
|  | etodolac | 1,478 | 12 | 271 | 44.3 | 22.9 – 77.3 |
|  | ibuprofen | 42,114 | 263 | 5,252 | 50.1 | 44.2 – 56.5 |
|  | indomethacin | 4,547 | 28 | 510 | 54.9 | 36.5 – 79.4 |
|  | meloxicam | 16,875 | 129 | 3,486 | 37.0 | 30.9 – 44.0 |
|  | nabumetone | 4,265 | 28 | 825 | 34.0 | 22.6 – 49.1 |
|  | naproxen | 20,687 | 161 | 3,236 | 49.7 | 42.4 – 58.1 |
|  | rofecoxib | 22,123 | 306 | 4,327 | 70.7 | 63.0 – 79.1 |
|  | valdecoxib | 6,521 | 33 | 1,234 | 26.7 | 18.4 – 37.6 |
| **AMI^‡^/**  **Ischemic stroke** | Overall | 181,061 | 2,053 | 32,655 | 62.9 | 60.2 – 65.7 |
|  | celecoxib | 50,508 | 680 | 11,522 | 59.0 | 54.7 – 63.6 |
|  | diclofenac | 11,931 | 127 | 2,113 | 60.1 | 50.1 – 71.5 |
|  | etodolac | 1,480 | 20 | 269 | 74.5 | 45.5 – 115.0 |
|  | ibuprofen | 42,117 | 367 | 5,219 | 70.3 | 63.3 – 77.9 |
|  | indomethacin | 4,547 | 64 | 505 | 126.8 | 97.7 – 161.9 |
|  | meloxicam | 16,877 | 149 | 3,464 | 43.0 | 36.4 – 50.5 |
|  | nabumetone | 4,265 | 36 | 821 | 43.9 | 30.7 – 60.7 |
|  | naproxen | 20,688 | 200 | 3,222 | 62.1 | 53.8 – 71.3 |
|  | rofecoxib | 22,124 | 343 | 4,293 | 79.9 | 71.7 – 88.8 |
|  | valdecoxib | 6,524 | 67 | 1,228 | 54.6 | 42.3 – 69.3 |
| **GIB/ICH**^§^ | Overall | 181,050 | 1,944 | 32,698 | 59.5 | 56.8 – 62.2 |
|  | celecoxib | 50,503 | 578 | 11,550 | 50.0 | 46.1 – 54.3 |
|  | diclofenac | 11,936 | 155 | 2,108 | 73.5 | 62.4 – 86.1 |
|  | etodolac | 1,477 | 14 | 269 | 52.0 | 28.4 – 87.3 |
|  | ibuprofen | 42,118 | 284 | 5,235 | 54.2 | 48.1 – 60.9 |
|  | indomethacin | 4,548 | 80 | 506 | 158.0 | 125.3 – 196.6 |
|  | meloxicam | 16,871 | 158 | 3,470 | 45.5 | 38.7 – 53.2 |
|  | nabumetone | 4,265 | 32 | 819 | 39.1 | 26.7 – 55.1 |
|  | naproxen | 20,688 | 254 | 3,217 | 79.0 | 69.5 – 89.3 |
|  | rofecoxib | 22,123 | 341 | 4,294 | 79.4 | 71.2 – 88.3 |
|  | valdecoxib | 6,521 | 48 | 1,230 | 39.0 | 28.8 – 51.8 |

^*^p-ys: person-years. ^†^CI: confidence interval. **^‡^**AMI: acute myocardial infarction. ^§^GIB/ICH: gastrointestinal bleeding/intracranial hemorrhage.
