# Supplementary material for: Nonsteroidal anti-inflammatory drug choice and adverse outcomes in clopidogrel users: A retrospective cohort study
Source: PLoS One. 2018 Mar 14;13(3):e0193800. doi: 10.1371/journal.pone.0193800 (PMC5851628; doi:10.1371/journal.pone.0193800)
Supplement: S6 Table — (DOCX) [file pone.0193800.s011.docx]

**S6 Table. Secondary analysis: adjusted hazard ratios of outcomes by NSAID exposure group stratified by age, sex, and concomitancy triggering drug**

| **Category** | **NSAID** | **Mortality** | | **AMI/Ischemic stroke** | | **GI bleeding/Intracranial hemorrhage** | |
| --- | --- | --- | --- | --- | --- | --- | --- |
|  |  | **HR** | **95% CI** | **HR** | **95% CI** | **HR** | **95% CI** |
| **Age** | | | | | | | |
| 18 ≤ Age < 65 years | ibuprofen | *Reference drug* | | *Reference drug* | | *Reference drug* | |
|  | celecoxib | 0.87 | 0.62 – 1.20 | 0.88 | 0.70 – 1.12 | 0.89 | 0.66 – 1.19 |
|  | diclofenac | 0.84 | 0.49 – 1.44 | 1.10 | 0.78 – 1.56 | 1.60 | 1.09 – 2.34 |
|  | etodolac | 0.68 | 0.21 – 2.15 | 1.50 | 0.88 – 2.57 | 1.26 | 0.60 – 2.61 |
|  | indomethacin | 1.21 | 0.65 – 2.26 | 1.55 | 1.04 – 2.30 | 2.39 | 1.53 – 3.75 |
|  | meloxicam | 0.72 | 0.43 – 1.19 | 0.95 | 0.69 – 1.32 | 0.97 | 0.65 – 1.45 |
|  | nabumetone | 0.84 | 0.40 – 1.75 | 0.76 | 0.46 – 1.26 | 0.67 | 0.34 – 1.33 |
|  | naproxen | 0.88 | 0.62 – 1.24 | 0.87 | 0.68 – 1.11 | 1.28 | 0.98 – 1.69 |
|  | rofecoxib | 0.97 | 0.64 – 1.48 | 0.94 | 0.70 – 1.27 | 1.31 | 0.92 – 1.87 |
|  | valdecoxib | 0.34 | 0.11 – 1.10 | 0.66 | 0.37 – 1.18 | 0.34 | 0.12 – 0.94 |
| 65 ≤ Age ≤ 100 years | ibuprofen | *Reference drug* | | *Reference drug* | | *Reference drug* | |
|  | celecoxib | 1.05 | 0.91 – 1.22 | 0.96 | 0.84 – 1.11 | 0.91 | 0.78 – 1.05 |
|  | diclofenac | 1.17 | 0.94 – 1.46 | 1.13 | 0.92 – 1.39 | 1.69 | 1.39 – 2.06 |
|  | etodolac | 0.79 | 0.43 – 1.48 | 1.09 | 0.68 – 1.77 | 1.33 | 0.85 – 2.09 |
|  | indomethacin | 1.11 | 0.79 – 1.55 | 1.28 | 0.96 – 1.72 | 2.17 | 1.67 – 2.82 |
|  | meloxicam | 0.93 | 0.77 – 1.13 | 0.88 | 0.73 – 1.06 | 1.21 | 1.01 – 1.46 |
|  | nabumetone | 1.07 | 0.78 – 1.47 | 0.85 | 0.61 – 1.17 | 0.81 | 0.57 – 1.15 |
|  | naproxen | 1.13 | 0.94 – 1.36 | 1.06 | 0.89 – 1.26 | 1.55 | 1.31 – 1.84 |
|  | rofecoxib | 1.28 | 1.07 – 1.52 | 1.15 | 0.97 – 1.37 | 1.28 | 1.07 – 1.53 |
|  | valdecoxib | 0.74 | 0.52 – 1.04 | 0.91 | 0.68 – 1.22 | 0.82 | 0.60 – 1.11 |
| **Sex** | | | | | | | |
| Male | ibuprofen | *Reference drug* | | *Reference drug* | | *Reference drug* | |
|  | celecoxib | 1.17 | 0.94 – 1.44 | 1.00 | 0.83 – 1.22 | 0.86 | 0.69 – 1.07 |
|  | diclofenac | 1.26 | 0.92 – 1.73 | 1.31 | 1.00 – 1.73 | 1.57 | 1.18 – 2.11 |
|  | etodolac | 1.24 | 0.61 – 2.54 | 1.56 | 0.90 – 2.71 | 1.55 | 0.83 – 2.89 |
|  | indomethacin | 0.92 | 0.58 – 1.46 | 1.44 | 1.03 – 2.02 | 2.08 | 1.50 – 2.90 |
|  | meloxicam | 0.97 | 0.72 – 1.30 | 0.85 | 0.64 – 1.12 | 1.19 | 0.91 – 1.56 |
|  | nabumetone | 1.02 | 0.62 – 1.67 | 0.68 | 0.41 – 1.13 | 0.72 | 0.41 – 1.27 |
|  | naproxen | 1.14 | 0.89 – 1.47 | 1.15 | 0.92 – 1.43 | 1.30 | 1.03 – 1.64 |
|  | rofecoxib | 1.41 | 1.07 – 1.87 | 1.13 | 0.88 – 1.45 | 1.20 | 0.91 – 1.57 |
|  | valdecoxib | 0.89 | 0.52 – 1.53 | 0.72 | 0.44 – 1.17 | 0.62 | 0.35 – 1.10 |
| Female | ibuprofen | *Reference drug* | | *Reference drug* | | *Reference drug* | |
|  | celecoxib | 0.93 | 0.79 – 1.10 | 0.91 | 0.78 – 1.06 | 0.90 | 0.76– 1.06 |
|  | diclofenac | 1.01 | 0.78 – 1.31 | 1.00 | 0.79 – 1.26 | 1.71 | 1.38 – 2.13 |
|  | etodolac | 0.42 | 0.17 – 1.04 | 1.09 | 0.68 – 1.74 | 1.15 | 0.71 – 1.88 |
|  | indomethacin | 1.39 | 0.95 – 2.04 | 1.35 | 0.97 – 1.88 | 2.28 | 1.67 – 3.12 |
|  | meloxicam | 0.83 | 0.67 – 1.04 | 0.90 | 0.73 – 1.09 | 1.14 | 0.93 – 1.40 |
|  | nabumetone | 0.98 | 0.69 – 1.39 | 0.90 | 0.65 – 1.24 | 0.81 | 0.56 – 1.18 |
|  | naproxen | 1.02 | 0.83 – 1.26 | 0.90 | 0.74 – 1.08 | 1.60 | 1.33 – 1.91 |
|  | rofecoxib | 1.14 | 0.93 – 1.39 | 1.06 | 0.88 – 1.28 | 1.29 | 1.06 – 1.57 |
|  | valdecoxib | 0.58 | 0.38 – 0.86 | 0.89 | 0.66 – 1.21 | 0.77 | 0.55 – 1.09 |
| **Concomitancy triggering drug** | | | | | | | |
| NSAID-triggered group* | ibuprofen | *Reference drug* | | *Reference drug* | | *Reference drug* | |
|  | celecoxib | 0.93 | 0.79 – 1.10 | 0.91 | 0.78 – 1.06 | 0.90 | 0.76– 1.06 |
|  | diclofenac | 1.01 | 0.78 – 1.31 | 1.00 | 0.79 – 1.26 | 1.71 | 1.38 – 2.13 |
|  | etodolac | 0.42 | 0.17 – 1.04 | 1.09 | 0.68 – 1.74 | 1.15 | 0.71 – 1.88 |
|  | indomethacin | 1.39 | 0.95 – 2.04 | 1.35 | 0.97 – 1.88 | 2.28 | 1.67 – 3.12 |
|  | meloxicam | 0.83 | 0.67 – 1.04 | 0.90 | 0.73 – 1.09 | 1.14 | 0.93 – 1.40 |
|  | nabumetone | 0.98 | 0.69 – 1.39 | 0.90 | 0.65 – 1.24 | 0.81 | 0.56 – 1.18 |
|  | naproxen | 1.02 | 0.83 – 1.26 | 0.90 | 0.74 – 1.08 | 1.60 | 1.33 – 1.91 |
|  | rofecoxib | 1.14 | 0.93 – 1.39 | 1.06 | 0.88 – 1.28 | 1.29 | 1.06 – 1.57 |
|  | valdecoxib | 0.58 | 0.38 – 0.86 | 0.89 | 0.66 – 1.21 | 0.77 | 0.55 – 1.09 |
| Clopidogrel-triggered group^†^ and combination-triggered group^‡^ | ibuprofen | *Reference drug* | | *Reference drug* | | *Reference drug* | |
|  | celecoxib | 0.93 | 0.79 – 1.10 | 0.91 | 0.78 – 1.06 | 0.90 | 0.76– 1.06 |
|  | diclofenac | 1.01 | 0.78 – 1.31 | 1.00 | 0.79 – 1.26 | 1.71 | 1.38 – 2.13 |
|  | etodolac | 0.42 | 0.17 – 1.04 | 1.09 | 0.68 – 1.74 | 1.15 | 0.71 – 1.88 |
|  | indomethacin | 1.39 | 0.95 – 2.04 | 1.35 | 0.97 – 1.88 | 2.28 | 1.67 – 3.12 |
|  | meloxicam | 0.83 | 0.67 – 1.04 | 0.90 | 0.73 – 1.09 | 1.14 | 0.93 – 1.40 |
|  | nabumetone | 0.98 | 0.69 – 1.39 | 0.90 | 0.65 – 1.24 | 0.81 | 0.56 – 1.18 |
|  | naproxen | 1.02 | 0.83 – 1.26 | 0.90 | 0.74 – 1.08 | 1.60 | 1.33 – 1.91 |
|  | rofecoxib | 1.14 | 0.93 – 1.39 | 1.06 | 0.88 – 1.28 | 1.29 | 1.06 – 1.57 |
|  | valdecoxib | 0.58 | 0.38 – 0.86 | 0.89 | 0.66 – 1.21 | 0.77 | 0.55 – 1.09 |

AMI: acute myocardial infarction. GI: gastrointestinal. HR: hazard ratio. CI: confidence interval.

*NSAID-triggered group: subgroup in which the concomitant use of clopidogrel and an NSAID was initiated by an NSAID during the ongoing clopidogrel treatment. ^†^Clopidogrel-triggered group: subgroup in which the concomitant use of clopidogrel and an NSAID was initiated by clopidogrel during the ongoing NSAID treatment. ‡Combination-triggered group: subgroup in which the concomitant use of clopidogrel and an NSAID was initiated by both clopidogrel and an NSAID on the same day, i.e., prescriptions of clopidogrel and an NSAID dispensed on the same day. Number of events are not presented by the cell size suppression policy of the Centers for Medicare and Medicaid Services.
